# Zinc deficiency activates S100A8 inflammation in the absence of COX-2 and promotes murine oral-esophageal tumor progression

**DOI:** 10.1002/ijc.25688

**Published:** 2010-09-20

**Authors:** Shao-Gui Wan, Cristian Taccioli, Yubao Jiang, Hongping Chen, Karl J Smalley, Kun Huang, Xiu-Ping Liu, John L Farber, Carlo M Croce, Louise Y Y Fong

**Affiliations:** 1 Department of Pharmacology and Experimental Therapeutics, Thomas Jefferson UniversityPhiladelphia, PA; 2 Department of Molecular Virology, Immunology, and Medical Genetics, The Ohio State UniversityOH; 3 Kimmel Cancer Center, Thomas Jefferson UniversityPhiladelphia, Pennsylvania, PA; 4 Department of Biomedical Informatics, The Ohio State UniversityColumbus, OH; 5 Department of Pathology, Anatomy, and Cell Biology, Thomas Jefferson UniversityPhiladelphia, PA

**Keywords:** zinc deficiency, transcriptome profiling, Cox-2 null mice, S100A8 inflammation, tongue cancer prevention

## Abstract

Zinc (Zn)-deficiency (ZD) is implicated in the pathogenesis of human oral-esophageal cancers. Previously, we showed that in ZD mice genetic deletion of *cyclooxygenase-2* (*Cox-2*) enhances *N*-nitrosomethylbenzylamine-induced forestomach carcinogenesis. By contrast, *Cox-2* deletion offers protection in Zn-sufficient (ZS) mice. We hypothesize that ZD activates pathways insensitive to COX-2 inhibition, thereby promoting carcinogenesis. This hypothesis is tested in a *Cox-2*^−/−^ mouse tongue cancer model that mimics pharmacologic blockade of COX-2 by firstly examining transcriptome profiles of forestomach mucosa from *Cox-2*^−/−^ and wild-type mice on a ZD *vs*. ZS diet, and secondly investigating the roles of identified markers in mouse forestomach/tongue preneoplasia and carcinomas. In *Cox-2*^−/−^ mice exposed to the tongue carcinogen 4-nitroquinoline 1-oxide, dietary ZD elicited tongue/esophagus/forestomach carcinomas that were prevented by ZS. The precancerous ZD:*Cox-2*^−/−^*vs*. ZS:*Cox-2*^−/−^ forestomach had an inflammatory signature with upregulation of the proinflammation genes *S100a8* and *S100a9*. Bioinformatics analysis revealed overrepresentation of inflammation processes comprising *S100a8*/*a9* and an nuclear factor (NF)-κB network with connectivity to S100A8. Immunohistochemistry revealed co-overexpression of S100A8, its heterodimeric partner S100A9, the receptor for advanced glycation end-products (RAGE), NF-κB p65, and cyclin D1, in ZD:*Cox-2*^−/−^ forestomach/tongue preneoplasia and carcinomas, evidence for the activation of a RAGE-S100A8/A9 inflammatory pathway. Accumulation of p53 in these carcinomas indicated activation of additional inflammatory pathways. Zn-replenishment in ZD:*Cox-2*^−/−^mice reversed the inflammation and inhibited carcinogenesis. Thus, ZD activates alternative inflammation-associated cancer pathways that fuel tumor progression and bypass the antitumor effect of *Cox-2* ablation. These findings have important clinical implications, as combination cancer therapy that includes Zn may improve efficacy.

Oral-esophageal squamous cell carcinomas (SCCs) are a major cause of cancer deaths worldwide.^1^ Oral cancer, the major site being the tongue, causes a high mortality rate because of frequent development of a second primary esophageal cancer because of field cancerization effects.^2^ Risk factors include alcohol consumption, tobacco and human papillomavirus (HPV).^3^ The incidence of oral cancer is increasing, particularly in young adults without documented risk factors.^3^ Epidemiologic and clinical studies have long implicated zinc (Zn)-deficiency (ZD) in the pathogenesis of oral-esophageal cancers in many populations.^4^–^6^ ZD is associated with increased tumor size and poor disease prognosis.^4^,^7^

Zn is required for the activity of many enzymes, for proper immune function, and for the conformation of many transcription factors that control cell proliferation, apoptosis, and signaling pathways.^8^,^9^ Zn is known to undergo rapid ligand exchange reactions and is used as an information carrier in signal transduction pathways similar to calcium.^10^ Consequently, ZD predisposes to disease by adversely affecting immune system, by increasing oxidative stress, and by increasing the generation of inflammatory cytokines.^11^ Although the role of ZD as a causative factor of disease and as a determinant in disease progression is gaining attention,^12^ the mechanisms underlying its protumorigenic effect, however, remain unclear.

In the rat, a ZD diet creates a precancerous condition in the upper digestive tract, including tongue, esophagus and forestomach (an expanded lower esophagus), by inducing proliferation^13^ and gene expression changes, including overexpression of *cyclooxygenase-2* (*Cox-2*) and the proinflammation-genes *S100 calcium binding protein a8* (*S100a8*) and *a9* (*S100a9*).^13^,^14^ ZD rats rapidly develop esophageal tumors after a single exposure to the environmental carcinogen *N*-nitrosomethylbenzylamine (NMBA)^15^ and concurrent tongue, esophageal and forestomach tumors with exposure to the tongue carcinogen 4-nitroquinoline 1-oxide (NQO).^13^ Zn-replenishment (ZR) reverses cell proliferation, corrects gene expression and inhibits carcinogenesis.^14^–^16^

Targeted therapies that block molecules crucial to tumor growth are being explored in attempts to prevent or cure cancer.^17^ The rationale for targeting the COX-2 pathway is supported by numerous studies. COX-2 is overexpressed in many human cancers, including esophageal and tongue SCC.^18^,^19^ COX-2 catalyzes the formation of prostaglandins and is induced by factors implicated in carcinogenesis, including growth factors, inflammatory stimuli, oncogenes and tumor promoters.^20^ The report that deletion of the *Cox-2* gene in *Apc* knockout mice greatly reduces intestinal polyp formation provides genetic evidence that COX-2 plays a key role in tumorigenesis.^21^ COX-2 selective inhibitors, celecoxib in particular, are being tested in clinical trials for the prevention of several cancers,^22^ including esophageal cancer.^23^ Although such targeted therapies have shown promising results in several cancers, their efficacy in oral-esophageal cancer has been limited.^24^

Our previous work showed that in ZD rats pharmacologic COX-2 inhibition by the drug celecoxib did not prevent tongue carcinogenesis, and in ZD mice genetic *Cox-2* deletion actually enhanced NMBA-induced forestomach tumorigenesis.^16^ Aside from the result that ZD:*Cox-2*^−/−^ mouse forestomach overexpressed leukotriene A_4_ hydrolase protein, indicating a shift of arachidonic acid to the 5-lipoxygenase pathway, mechanisms underlying this effect of ZD were not elucidated. We hypothesized that ZD adversely affects treatment outcome by stimulating pathways not inhibited by the pharmacologic blockade of COX-2. We tested this hypothesis in a ZD:*Cox-2*^−/−^ mouse oral-esophageal cancer model that mimics pharmacologic COX-2 blockade, using techniques that included transcriptome profiling, bioinformatics analyses, and investigation of the pathobiological roles of identified markers in murine tongue/forestomach preneoplasia and neoplasia.

## Material and Methods

### Mice, diets and carcinogens

We bred heterozygous B6;129S7-*Ptgs2*^*tm1Jed*^/J males to females (Jackson Laboratory, Bar Harbor, ME) to generate *Cox-2*^−/−^, *Cox-2*^+/−^ and *Cox-2*^+/+^ (WT) littermates.^16^ Custom-formulated ZD and Zn-sufficient (ZS) diets (Harlan Teklad, Madison, WI) were identical except for the Zn content.^16^ NQO was from Wako Chemicals (Richmond, VA) and NMBA from Midwest Research Institute (Kansas City, MI).

### NQO-induced tongue carcinogenesis

The mouse studies were approved by The Ohio State University Animal Use Committee. Four-week old littermates were fed ZD or ZS diets to form six groups, namely, ZD:*Cox-2*^−/−^ (*n* = 14), ZD:*Cox-2*^+/−^ (*n* = 46), ZD:WT (*n* = 19), ZS:*Cox-2*^−/−^ (*n* = 16), ZS:*Cox-2*^+/−^ (*n* = 37) and ZS:WT (*n* = 25). After 4 weeks the mice were administered NQO in deionized water for tongue tumor induction (20 ppm for 19 weeks followed by 30 ppm for 7 weeks). At 26 weeks, the animals were sacrificed for tumor incidence analysis.

### Expression profiling and related studies

Weanling *Cox-2*^−/−^ and *Cox-2*^+/+^ mice were fed ZD or ZS diets to form four groups, namely, ZD:*Cox-2*^−/−^ (*n* = 20), ZS:*Cox-2*^−/−^ (*n* = 12), ZD:WT (*n* = 12) and ZS:WT (*n* = 12). After 9 weeks, 8 ZD:*Cox-2*^−/−^ mice were switched to a ZS diet to form the ZR:*Cox-2*^−/−^ group. After a week, all mice were sacrificed. This experimental regimen produced unbridled cell proliferation in ZD:*Cox-2*^−/−^ forestomach.^16^ Tongue and forestomach were isolated and cut into two portions. One portion was formalin-fixed and paraffin-embedded for immunohistochemical (IHC) studies. Forestomach epithelia for expression profiling studies were prepared from the remaining portion by using a blade to strip off the submucosal layers and snap-frozen in liquid nitrogen.

We performed expression profiling of forestomach mucosa from ZD:*Cox-2*^−/−^, ZS:*Cox-2*^−/−^, ZD:WT mice and ZS:WT mice after 10 weeks of ZD or ZS diets (*n* = 4 mice/group), using GeneChip® Mouse Genome 430 2.0 Array (Affymetrix, Santa Clara, CA). Total RNA was extracted from forestomach mucosa using TRIZOL reagent (Invitrogen, Carlsbad, CA). Five micrograms of total RNA was reverse transcribed into cDNA followed by *in vitro* transcription and labeling to produce biotin-labeled cRNA. The cRNA was hybridized to the arrays as described.^14^

### Expression data analysis

We used the Class Comparison analysis of BRB-Array Tools software version 3.7.0 (Biometric Research Branch, NCI) to identify differentially expressed mRNAs. The Robust Multichip Average method was performed. The array data were submitted to ArrayExpress (Accession number: E-TABM-778).

### Gene ontology and pathway analyses

We used DAVID (Database for Annotation, Visualization and Integrated Discovery)^25^ bioinformatics to identify relevant biological processes/functions from expression data captured by transcriptome analysis. Based on gene ontology, differentially expressed genes were grouped by scoring the statistical significance of predefined functional gene groups according to their functional similarity.

We used Ingenuity Pathway Analysis software (IPA, http://www.ingenuity.com) to analyze probable network/pathway and functional group enrichment. For each data set, the selected genes were uploaded into the IPA application. Networks were then algorithmically generated based on gene–gene connectivity.

### ZR and forestomach carcinogenesis in ZD:Cox-2^−/−^ mice

This mouse study was approved by the Thomas Jefferson University Animal Use Committee. Thirty-nine 4-week old *Cox-2*^−/−^ mice were fed a ZD diet to form the ZD:*Cox-2*^−/−^ group. After 4 weeks, the mice received three intragastric doses of NMBA (2 mg/kg body weight, twice weekly), a regimen that produced a high tumor outcome in ZD:*Cox-2*^−/−^ mice.^16^ A day after the 3rd dose, 18 mice were switched to a ZS diet to form the ZR:*Cox-2*^−/−^ group, which were given an intragastric dose of Zn gluconate weekly for 14 weeks (0.04 mg Zn). The remaining ZD:*Cox-2*^−/−^ mice continued on ZD diet. All mice were sacrificed for tumor outcome analysis at 14 weeks of Zn intervention.

### Tumor analysis

At autopsy, tongue, esophagus and forestomach were excised. Tumors greater than 0.5 mm were mapped. Tissues were formalin-fixed and paraffin-embedded for histopathologic/IHC studies.

### Quantitative reverse transcriptase-polymerase chain reaction

Quantitative reverse transcriptase-polymerase chain reaction (qRT-PCR) was performed using the comparative C_t_ method and predesigned probes on 7300 Real-time PCR System (Applied Biosystems, Foster City, CA). GAPDH was used to normalize RNA samples.^14^

### Immunoblotting

Proteins were separated by 10–14% Tris-HCl gel (Bio-Rad, Hercules, CA) as described.^13^ GAPDH (Calbiochem, San Diego, CA) was used as a loading control.

### IHC

IHC was performed as described.^13^–^16^ The following antisera were used: mouse anti-proliferating cell nuclear antigen (PCNA) monoclonal (Thermo Scientific); rat anti-S100A8 monoclonal, goat anti-S100A9 monoclonal, and rat anti-receptor for advanced glycation end-products (RAGE) monoclonal (R&D Systems, Minneapolis, MN); rabbit anti-nuclear factor (NF)-κB p65 polyclonal (Abcam, Cambridge, MA), rabbit anti-NF-κB phospho-p65 (serine 276) polyclonal and rabbit anti-cyclin D1 monoclonal antiserum (Cell Signaling, Danvers, MA) or rabbit anti-p53 polyclonal antiserum (detects both mutated and wild-type proteins) (Leica Microsystems, Bannockburn, IL). Protein was localized by incubation with 3-amino-9-ethylcarbazole substrate-chromogen (AEC) (Dako, Carpinteria, CA) or 3,3′-diaminobenzidine tetrahydrochloride (DAB; Sigma-Aldrich, St. Louis, MO).

Immunoreactive scores were calculated by multiplying the percentage of positive cells by the grade of staining intensity.^15^ The percentage of positive cells was evaluated as follows: 0 = 0–5%, 1 = 6–25%, 2 = 26–50%, 3 = 51–75% and 4 = 76–100%. The intensity of immunostaining was graded as follows: 0 = none, 1 = weak, 2 = moderate and 3 = intense. The PCNA labeling index (%) was calculated by dividing the number of PCNA-labeled nuclei by the total number of cells counted.

### Zn measurement

Hair Zn content was determined by atomic absorption spectrometry.^16^ Invariably, Zn levels were significantly lower in ZD than ZS samples. As examples, hair Zn levels were significantly lower in ZD:*Cox-2*^−/−^ than ZS:*Cox-2*^−/−^ mice at 10 weeks (array study) [130 μg/g (95% confidence interval [CI] = 124–135) *vs*. 172 μg/g (95% CI = 165–179), *p* = 0.002, *n* = 10/group) and at 26 weeks (NQO study); 111 μg/g (95% CI = 99–122) *vs*. 157 μg/g (95% CI = 146–168), *n* = 14 mice/group, *p* < 0.001].

### Statistical analysis

Tumor multiplicity was analyzed by two-way analysis of variance (ANOVA). Differences among the groups were assessed using the Tukey-HSD post hoc *t*-tests for multiple comparisons. Tumor and carcinoma incidence rates were assessed by Fisher's exact test. CIs for the differences in incidence rates were calculated using the Wilson Score Method.^26^ Statistical tests were two-sided and considered significant at *p* < 0.05.

## Results

### ZD enhances tongue carcinogenesis in *Cox-2* deficient mice

NQO is a DNA adduct-forming agent that serves as a surrogate of tobacco exposure.^27^ Nutritionally complete WT mice exposed to 10 ppm of NQO for 50 weeks did not develop tongue lesions.^28^ At a high concentration of 100 ppm, however, WT mice developed malignant tongue and esophageal tumors.^29^

**Figure 1:**
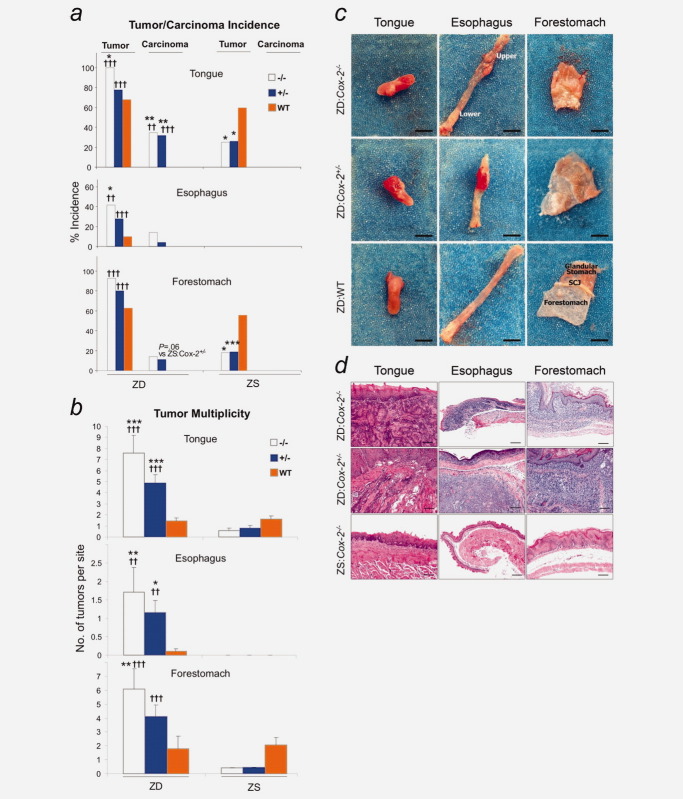
Zn-deficiency abolishes the antitumor effects of genetic *Cox-2* blockade in NQO-induced tongue carcinogenesis. *a*. Tumor incidence (%). ZS:*Cox-2*^−/−^ and ZS:*Cox-2*^+/−^ mice had significantly lower tongue/forestomach tumor incidence than ZS:WT mice (*p < 0.05, ***p < 0.001). By contrast, ZD:*Cox-2*^−/−^ had greater tongue/esophagus tumor incidence than ZD:WT mice (*p < 0.05). ZD:*Cox-2*^−/−^ and ZD:*Cox-2*^+/−^ mice had greater tongue/esophagus/forestomach tumor incidence than ZS:*Cox-2*^−/−^ and ZS:*Cox-2*^+/−^ mice (††*p* < 0.01, †††*p* < 0.001). Carcinoma incidence (%): ZD:*Cox-2*^−/−^ and ZD:*Cox-2*^+/−^ mice had higher tongue carcinoma incidence than ZD:WT mice (**p < 0.01). Also, ZD:*Cox-2*^−/−^ and ZD:*Cox-2*^+/−^ mice had greater tongue carcinoma incidence than respective ZS:*Cox-2*^−/−^ and ZS:*Cox-2*^+/−^ (††*p* < 0.01, †††*p* < 0.001). *b*. Tumor multiplicity (error bars = SEM). ZD:*Cox-2*^−/−^ and ZD:*Cox-2*^+/−^ mice had significantly more tumors/site (tongue, esophagus, forestomach) than ZD:WT counterpart (*p < 0.05, **p < 0.01, and ***p < 0.001). In addition, ZD:*Cox-2*^−/−^ and ZD:*Cox-2*^+/−^ mice had significantly more tumors/site than ZS:*Cox-2*^−/−^ and ZS:*Cox-2*^+/−^ counterpart (††*p* < 0.01, †††*p* < 0.001). *c*. Gross anatomy of tongue/esophagus/forestomach. Scale bars, 5 mm. *d*. H&E-stained sections showing tongue/esophageal/forestomach carcinomas from ZD:*Cox-2*^−/−^ and ZD:*Cox-2*^+/−^ and thickened mucosa from ZS:*Cox-2*^−/−^ mice. Scale bar = 100 μm. [Color figure can be viewed in the online issue, which is available at wileyonlinelibrary.com.]

To investigate whether a Zn-deficient condition eliminates the antitumor effect of genetic *Cox-2* disruption in NQO-induced tongue carcinogenesis as it does in NMBA-induced forestomach carcinogenesis,^16^*Cox-2*^−/−^, *Cox-2*^+/−^, and WT mice on ZD *vs*. ZS diets were exposed to drinking water containing 20 ppm of NQO for 19 weeks followed by 30 ppm for another 7 weeks. At week 26, ZS:*Cox-2*^−/−^ and ZS:*Cox-2*^+/−^ mice had significantly lower tongue/forestomach tumor incidence than ZS:WT littermates (Fig. 1*a*, statistical data in Supporting Information Table 1). This result is consistent with those reported in nutritionally complete mice showing that *Cox-2* absence protects against carcinogenesis.^16^,^21^,^30^ Conversely, in ZD mice, genetic *Cox-2* did not protect against carcinogenesis. ZD:*Cox-2*^−/−^ mice had significantly greater tongue/esophageal tumor incidence than ZD:WT littermates, and ZD:*Cox-2*^−/−^ and ZD:*Cox-2*^+/−^ mice showed significantly higher tumor multiplicity in all three sites (tongue, esophagus and forestomach) than ZD:WT controls (incidence, Fig. 1*a*; multiplicity, Fig. 1*b*, Supporting Information Table 1). In addition, ZD led to large tumor size (Fig. 1*c*) and malignant progression of tongue/esophageal/forestomach tumors in ZD:*Cox-2*^−/−^ and ZD:*Cox-2*^+/−^ mice compared with ZD:WT mice (Fig. 1*d*), with statistical significance achieved for tongue SCC (ZD:*Cox-2*^−/−^*vs*. ZD:WT, 35.7% [5 of 14] *vs*. 0% [0 of 19], *p* < 0.01; ZD:*Cox-2*^+/−^*vs*. ZD:WT, 32.6% [15 of 46] *vs*. 0% [0 of 19], *p* < 0.01) (Fig. 1*a*, Supporting Information Table 1). These data demonstrated that prolonged ZD abolished the antitumor effect of COX-2 blockade in tongue tumor prevention and elicited tumors in multiple sites with progression to malignancy.

Among mice of the same genetic background, tongue tumor incidence/multiplicity and carcinoma incidence were significantly higher in homozygous ZD:*Cox-2*^−/−^*vs*. ZS:*Cox-2*^−/−^ or heterozygous ZD:*Cox-2*^+/−^*vs*. ZS:*Cox-2*^+/−^ mice, but not in ZD:WT *vs*. ZS:WT mice (Fig. 1*a* and *b*, Supporting Information Table 1), demonstrating that combined ZD and *Cox-2* ablation led to a worse tumor outcome. These results are consistent with and extend our previous study in NMBA-induced forestomach carcinogenesis.^16^

### ZD *per se* induces an inflammatory gene signature in ZD:Cox-2^−/−^ forestomach

To test the hypothesis that ZD promotes carcinogenesis by activating cancer pathways not inhibited by genetic *Cox-2* ablation, we performed transcriptome profiling of forestomach mucosa from ZD:*Cox-2*^−/−^, ZS:*Cox-2*^−/−^, ZD:WT and ZS:WT mice (*n* = 4/group). We used forestomach rather than tongue because its epithelia can be readily separated from the muscularis layers without enzymatic digestion.

First, we examined the effect of ZD on gene expression changes in *Cox-2*^−/−^ forestomach and WT forestomach. By using a cutoff of *p* ≤ 0.05 and 2-fold difference in expression levels, we found 314 dysregulated probe sets in ZD:*Cox-2*^−/−^*vs*. ZS:*Cox-2*^−/−^ forestomach (Supporting Information Table 2) but only 67 in ZD:WT *vs*. ZS:WT forestomach (Supporting Information Table 3). Thus, dietary ZD causes more extensive changes in gene expression in *Cox-2*^−/−^ than WT forestomach. A cohort of 36 genes, including the proinflammation mediators *S100a8*/*a9*, small proline-rich protein 2 *Sprr2f*/*2h*, and keratins *Krt6a*/*8*/*19*, was common to both class comparisons, indicating that these genes were induced by ZD regardless of genotype.

Next, we compared the effect of *Cox-2* deletion on gene expression changes in ZD forestomach and in ZS forestomach. With a cutoff of 2-fold difference, we found 90 dysregulated genes in ZD:*Cox-2*^−/−^*vs*. ZD:WT forestomach (Supporting Information Table 4) but only 17 in ZS:*Cox-2*^−/−^*vs*. ZS:WT forestomach (Supporting Information Table 5). There are no common changes in gene expression between these two class comparisons, and *Cox-2* deletion causes fewer changes in ZS than ZD forestomach.

Our qRT-PCR data validated a total 12 selected genes for ZD:*Cox-2*^−/−^*vs*. ZS:*Cox-2*^−/−^ forestomach; six genes for ZD:WT *vs*. ZS:WT, 7 genes for ZD:*Cox-2*^−/−^*vs*. ZD:WT, and three genes for ZS:*Cox-2*^−/−^*vs*. ZS:WT forestomach (Supporting Information Table 6).

**Figure 2:**
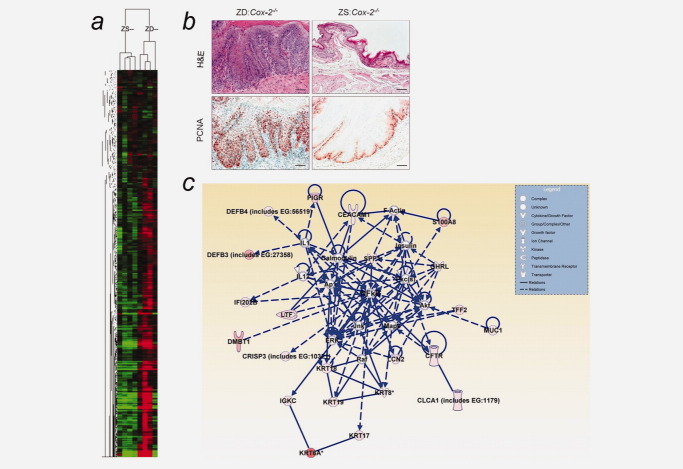
Expression profiling of forestomach mucosa. *a*. Dendrogram illustrating clustering of ∼45,000 transcripts in ZD:*Cox-2*^−/−^ and ZS:*Cox-2*^−/−^ forestomach (*n* = 4 mice/group). Red and green denote up- and downregulated genes. *b*. H&E- and PCNA-stained (*red*, AEC) hyperplastic ZD:*Cox-2*^−/−^ forestomach and ZS:*Cox-2*^−/−^ forestomach. Scale bar = 50 μm. *c*. IPA uncovers a NF-κB network from the upregulated genes (red) in ZD:*Cox-2*^−/−^*vs*. ZS:*Cox-2*^−/−^ forestomach. IPA inserts genes (white) to complete the network. Solid and dash lines reflect, respectively, direct and indirect relationships among the genes. [Color figure can be viewed in the online issue, which is available at wileyonlinelibrary.com.]

Among the four class comparisons (Supporting Information Tables 2–5), ZD:*Cox-2*^−/−^*vs*. ZS:*Cox-2*^−/−^ forestomach showed the most extensive changes in gene expression, a result consistent with their divergent tumorigenic potential^16^ (Fig. 1). Hierarchical clustering analysis of 45,000 transcripts revealed distinct expression patterns (Fig. 2*a*) between hyperplastic ZD:*Cox-2*^−/−^ and nonproliferative ZS:*Cox-2*^−/−^ forestomach (Fig. 2*b*). By further filtering the data using a cutoff of 4-fold difference, we identified a group of 63 genes (62 up- and 1 downregulated; Table 1). The most upregulated genes are implicated in the following processes: *Sprr2h*/*2f* and *Krt6a*/*16* in cytoskeleton metabolism and *S100a8* and *S100a9* (upregulated 24- and 2.2-fold) in inflammatory/defense/immune responses. Interestingly, *S100a8*/*a9* were also upregulated 4.2- and 2.4-fold in ZD:WT *vs*. ZS:WT forestomach (Supporting Information Table 3). Because *S100a8*/*a9* overexpression is associated with ZD-induced rat esophageal preneoplasia,^14^ the data that these same genes were upregulated by ZD in hyperplastic ZD:*Cox-2*^−/−^ forestomach indicate that they are relevant ZD-induced markers in early forestomach carcinogenesis.

**Table 1:** Gene expression signature in hyperplastic ZD:*Cox-2*^-/-^ vs non-proliferative ZS:*Cox-2*^-/-^ mouse forestomach

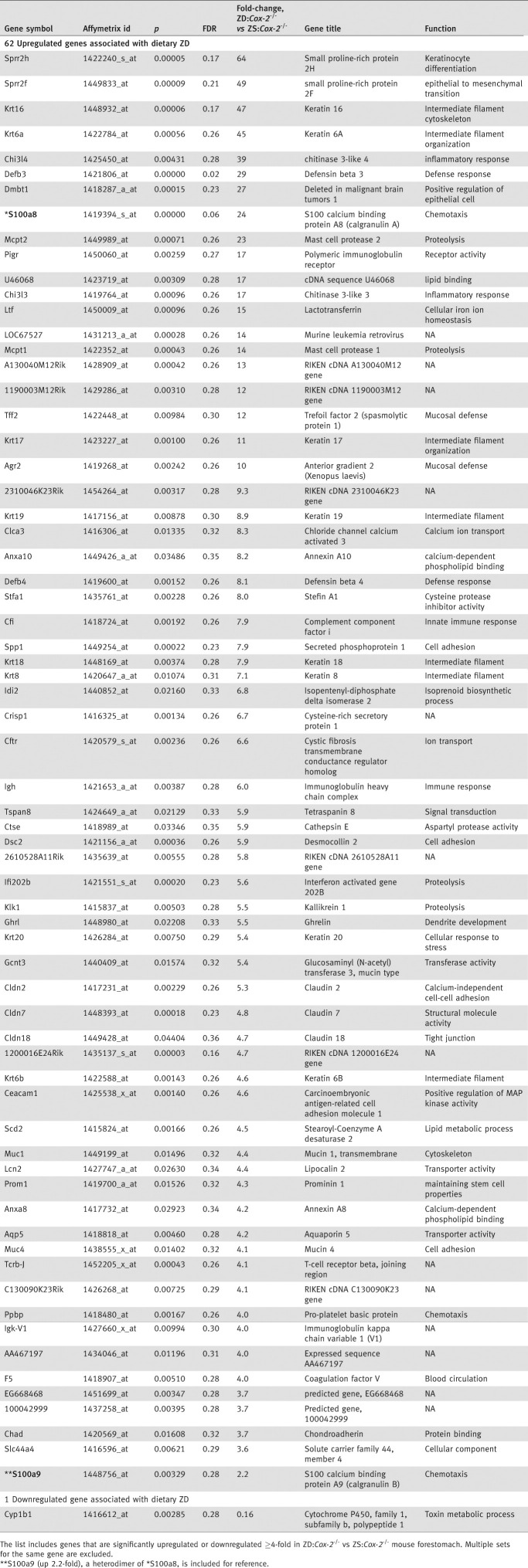

### DAVID bioinformatics reveals overrepresentation of inflammatory processes

To define the biological significance of the large lists of differentially expressed genes captured by our transcriptome profiling (Supporting Information Tables 2–5), we performed gene ontology functional group analyses for the four class comparisons using DAVID resources.^25^ In preneoplastic ZD:*Cox-2*^−/−^*vs*. ZS:*Cox-2*^−/−^ forestomach (Supporting Information Table 7*a*), we found significantly overrepresented biological processes only among the upregulated genes; including in particular, response to external stimulus comprising *S100a8*/a9 and 14 genes (*p* = 3.98E-004) and response to stimulus comprising *S100a8*/*a9* and 34 genes (*p* = 5.04E-004). Thus, DAVID supports the premise that *S100a8*/*a9* are relevant markers associated with ZD-induced hyperplasia in ZD:*Cox-2*^−/−^ forestomach. Similarly, in ZD:WT *vs*. ZS:WT (Supporting Information Table 7*b*) and ZD:*Cox-2*^−/−^*vs*. ZD:WT forestomach (Supporting Information Table 7*c*), significantly overrepresented processes were found only among the upregulated genes, including cytoskeleton and chemotaxis processes. By contrast, in ZS:*Cox-2*^−/−^*vs*. ZS:WT forestomach (Supporting Information Table 7*d*), significantly overrepresented processes were found only among the downregulated genes that negatively modulated cell cycle and cytoskeleton processes. Together, the data revealed that dietary ZD and sufficiency led to distinct regulated processes in proliferation in *Cox-2*^−/−^ forestomach, a finding consistent with the divergent tumorigenic potential of ZD:*Cox-2*^−/−^*vs*. ZS:*Cox-2*^−/−^ forestomach.

### IPA reveals a NF-κB—centric network

To understand gene expression interactions in ZD:*Cox-2*^−/−^*vs*. ZS:*Cox-2*^−/−^ forestomach (Table 1) in the context of signaling pathways, we performed pathway analysis using IPA. We identified a nuclear factor (NF)-κB centric network of 35 genes, with 60% of the genes (21 of 35) from the upregulated genes that included *S100a8* (Fig. 2*c*). Because NF-κB is a transcription factor that regulates immune responses/cell proliferation and it is a link between inflammation and cancer development/progression,^31^ our result that NF-κB showed connectivity to *S100a8* predicted activation of a S100A8-NF-κB inflammatory pathway in ZD:*Cox-2*^−/−^ forestomach.

### ZD activates S100A8 inflammatory signaling in preneoplastic ZD:Cox-2^−/−^ forestomach

We focused our study on S100A8 and its heterodimeric partner S100A9 because of their role in inflammation and cancer,^14^,^32^ and their prominence among ZD-induced proinflammation markers in ZD:*Cox-2*^−/−^ forestomach (Supporting Information Table 7*a*). *S100a8*/*a9* genes encode the S100 family member calcium binding proteins. Interaction of S100A8/A9 ligands with their receptor RAGE triggers an intracellular NF-κB signaling cascade.^14^,^32^

**Figure 3:**
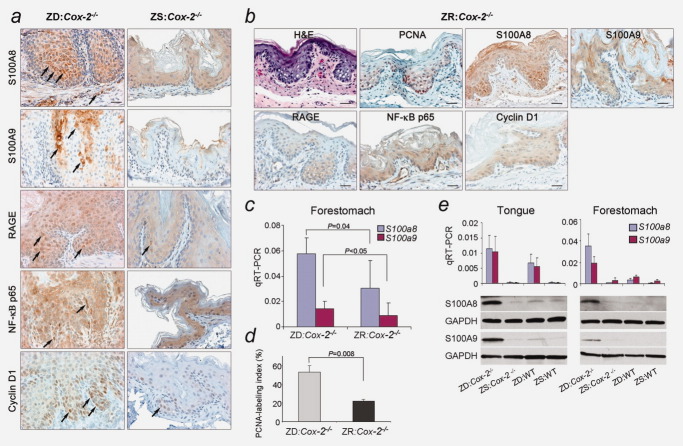
Zn modulates S100A8/A9 expression in *Cox-2*^−/−^ mouse forestomach and tongue. *a*. Zn-deficiency activates a RAGE-S100A8/9 inflammation pathway in ZD:*Cox-2*^−/−^ forestomach. Immunostaining of 5 markers (S100A8, S100A9, RAGE, NF-κB p65, and cyclin D1) in ZD:*Cox-2*^−/−^*vs*. ZS:*Cox-2*^−/−^ forestomach (*n* = 8 mice/group, DAB, brown). Scale bar = 25 μm. *b*. Zn-replenishment attenuates S100A8 inflammation in ZR:*Cox-2*^−/−^ forestomach (eight mice were analyzed). H&E, IHC of PCNA and the five markers (*red*, AEC). Scale bars = 50 μm. *c*. qRT-PCR analysis of *S100a8*/*a9* mRNA expression in ZR:*Cox-2*^−/−^*vs*. ZD:*Cox-2*^−/−^ forestomach (*n* = 6 mice/group). *d*. PCNA-labeling index (%) in ZR:*Cox-2*^−/−^*vs*. ZD:*Cox-2*^−/−^ forestomach (error bars = SEM; *n* = 6 mice/group). *e*. Upper panels: qRT-PCR analysis of *S100a8*/*a9* mRNA expression in tongue/forestomach from four mouse groups (*n* = 6 mice/group). Lower panels: immunoblotting of S100A8/A9 protein expression in tongue/forestomach (each band represents pooled samples from three mice). [Color figure can be viewed in the online issue, which is available at wileyonlinelibrary.com.]

To determine if there is a link between S100A8 overexpression and downstream NF-κB signaling in preneoplastic ZD:*Cox-2*^−/−^*vs*. ZS:*Cox-2*^−/−^ forestomach as predicted by IPA (Fig. 2*c*), we analyzed expression of five signaling markers, namely, S100A8, S100A9, the RAGE receptor, NF-κB p65, and cyclin D1, by IHC. The cyclin D1 gene is a target of NF-κB activation.^33^ IHC showed that all five markers were strongly expressed in hyperplastic ZD:*Cox-2*^−/−^ forestomach but weakly expressed in nonproliferative ZS:*Cox-2*^−/−^ forestomach (Fig. 3*a*). The semi-quantitative mean immunoreactive scores of S100A8 and S100A9 protein were significantly higher in ZD:*Cox-2*^−/−^ than ZS:*Cox-2*^−/−^ forestomach [S100A8, 7.3 (95% CI = 5.5–9.1) *vs*. 1.6 (95% CI = 0.9–2.3), *p* < 0.001; S100A9, 6.6 (95% CI = 3.7–9.5) *vs*. 1.5 (95% CI = 0.9–2.1), *p* < 0.001, *n* = 8 mice/group). In addition, ZD:*Cox-2*^−/−^ forestomach overexpressed phospho-NF-κB p65, indicating activation and nuclear translocation of NF-κB p65 (Supporting Information Fig. 1). These data show that at the earliest stages of forestomach carcinogenesis ZD activates an alternative S100A8 inflammatory pathway not affected by genetic *Cox-2* inhibition.

### ZR reverses S100A8 inflammatory signaling and hyperplasia in ZD:Cox-2^−/−^ forestomach

Because ZR attenuates inflammation and reverses hyperplasia in early rat esophageal carcinogenesis,^14^ we investigated this effect in ZD:*Cox-2*^−/−^ mice one week after switching to a ZS diet. In contrast to ZD:*Cox-2*^−/−^ forestomach that showed strong expression of all five S100A8 inflammatory signaling markers (Fig. 3*a* [left column]), ZR:*Cox-2*^−/−^ mouse forestomach had reduced or absent immunostaining of the same five markers (Fig. 3*b*). Additionally, qRT-PCR analysis shows that *S100a8* and *S100a9* mRNA expression was significantly reduced in ZR:*Cox-2*^−/−^*vs*. ZD:*Cox-2*^−/−^ forestomach (Fig. 3*c*).

In addition, we determined the rate of cell proliferation in ZR:*Cox-2*^−/−^*vs*. ZD:*Cox-2*^−/−^ forestomach by quantitative PCNA-lHC (PCNA: Fig. 3*b**vs*. 2*b*). PCNA is an endogenous cell proliferation marker. The PCNA-labeling index (%) was significantly lower in ZR:*Cox-2*^−/−^ than ZD:*Cox-2*^−/−^ forestomach (Fig. 3*d*). Together, these data (Fig. 3*b–d*) document that ZR effectively attenuated S100A8 inflammation and reversed the hyperplastic ZD:*Cox-2*^−/−^ phenotype.

### ZD upregulates S100a8/a9 expression in preneoplastic ZD:Cox-2^−/−^ tongue

To determine whether ZD:*Cox-2*^−/−^ tongue, which shows high tumorigenic potential as does ZD:*Cox-2*^−/−^ forestomach^16^ (Fig. 1), also overexpresses the proinflammation genes *S100a8*/*a9* discovered in forestomach, we determined *S100a8*/*a9* mRNA and protein expression levels in tongue and forestomach from the four mouse groups (profiling studies) by qRT-PCR and immunoblotting. As in forestomach, *S100a8*/*a9* mRNA expression was strongest in ZD:*Cox-2*^−/−^ tongue, followed by ZD:WT tongue, and negligible in ZS:*Cox-2*^−/−^ and ZS:WT tongue (Fig. 3*e*, top). In parallel, S100A8/A9 protein expression was strong in both ZD:*Cox-2*^−/−^ tongue and forestomach but weak or absent in similar tissues of other mouse groups (Fig. 3*e*, bottom). These data suggests that in tongue and forestomach ZD activates similar inflammatory pathways that are not affected by COX-2 inhibition.

### Activation of S100A8 and p53 inflammatory pathways accompanies malignant tumor progression in ZD:Cox-2^−/−^ and ZD:Cox-2^+/*−*^ mice

We then went on to investigate whether during malignant tongue/forestomach tumor progression (Fig. 1) S100A8 inflammatory signaling is in fact activated. In addition, we determined whether these carcinomas overexpress PCNA and p53 protein, two prognostic factors in human oral cancers.^34^,^35^ The *p53* tumor suppressor gene is mutated in approximately 50% of all human cancers, including oral-esophageal cancers^36^ and divergent carcinogenic pathways mediated separately by NF-κB and p53 were reported in oral cancer.^37^ Using IHC we examined expression of seven markers: PCNA, p53, and five S100A8—NF-κB signaling markers (S100A8, S100A9, RAGE, NF-κBp65 and cyclin D1). We analyzed a total of 15 ZD:Cox-*2*^−/−^ tongue SCC and 6 ZD:Cox-*2*^+/−^ forestomach SCC, as well as non-neoplastic ZS:*Cox-2*^−/−^ tongue and ZS:*Cox-2*^+/−^ forestomach (*n* = 10/group).

**Figure 4:**
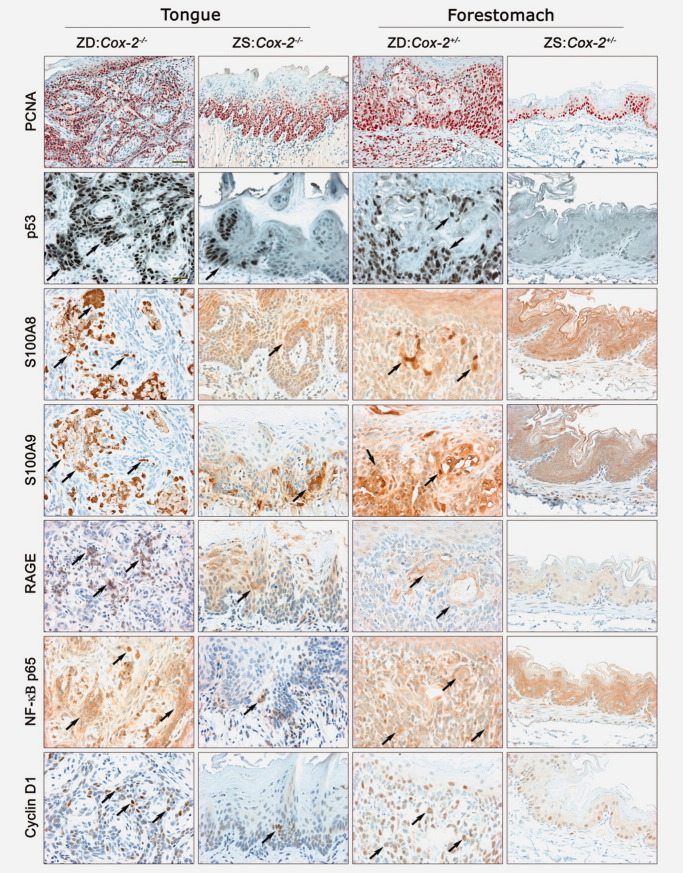
Zn-deficiency activates RAGE-S100A8/9 signaling and p53 accumulation in ZD:*Cox-2*^−/−^ and ZD:*Cox-2*^+/−^ tongue/forestomach carcinomas. IHC of 7 markers: PCNA, p53, S100A8, S100A9, RAGE, NF-κB p65 and cyclin D1 (*red*, AEC; *brown*, DAB; *black*, DAB with cobalt chloride). Scale bars, 50 μm (PCNA); 25 μm (all other markers). [Color figure can be viewed in the online issue, which is available at wileyonlinelibrary.com.]

ZD:*Cox-2*^−/−^ tongue SCC and ZD:*Cox-2*^+/−^ forestomach SCC showed high proliferative activity with abundant PCNA-positive nuclei in tumor areas and prominent accumulation of intensely stained p53-positive nuclei. Concurrently, these carcinomas displayed strong co-overexpression of all five S100A8—NF-κB signaling markers (Fig. 4). In addition, these carcinomas overexpressed phospho-NF-κB p65 (Supporting Information Fig. 1), indicating activation and nuclear translocation of NF-κB p65. Collectively, these data demonstrate that under complete or partial genetic *Cox-2* ablation, ZD stimulated RAGE-S100A8 inflammatory signaling cancer-and p53-associated response pathways, thereby driving malignant tumor progression and bypassing the antitumor effect of COX-2 blockade.

In sharp contrast, non-neoplastic ZS:*Cox-2*^−/−^ tongue and ZS:*Cox-2*^+/−^ forestomach showed basal cell proliferation with isolated occurrence of p53 protein, as well as low levels of expression of the same five S100A8 signaling markers (Fig. 4), providing evidence that inflammatory pathways were not activated under conditions of COX-2 pathway blockade and ZS that protected against carcinogenesis.

### ZR attenuates the inflammation and restores the antitumor effect of COX-2 blockade in cancer prevention

**Figure 5:**
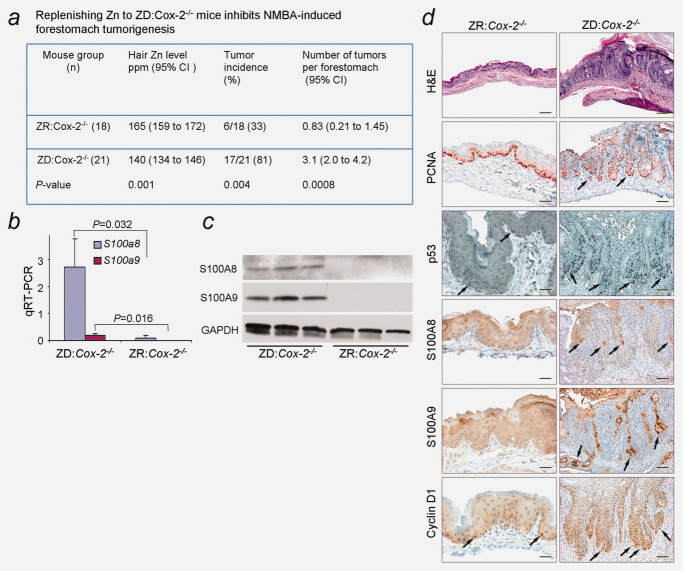
Zn-replenishment reverses inflammatory responses and inhibits NMBA-induced forestomach carcinogenesis. *a*. Tumor data. *b*. qRT-PCR analysis of *S100a8*/*a9* mRNA expression in ZR:*Cox-2*^−/−^ and ZD:*Cox-2*^−/−^ forestomach (error bars = SEM; *n* = 6 mice/group). *c*. Immunoblotting of S100A8/A9 protein expression in ZR:*Cox-2*^−/−^ and ZD:*Cox-2*^−/−^ forestomach. *d*. H&E and IHC of PCNA, p53, S100A8, S100A9 and cyclin D1, in ZR:*Cox-2*^−/−^*vs*. ZD:*Cox-2*^−/−^ forestomach (15 mice/group were analyzed). Scale bars, 100 μm (H&E); 50 μm (PCNA; ZD:*Cox-2*^−/−^: p53, S100A8, S100A9, cyclin D1); 25 μm (ZR:*Cox-2*^−/−^ : p53, S100A8, S100A9, cyclinD1). [Color figure can be viewed in the online issue, which is available at wileyonlinelibrary.com.]

Finally, we investigated whether replenishing Zn can restore the antitumor effect of COX-2 blockade in tumor prevention. In a NMBA-induced forestomach carcinogenesis study, we showed that 14 weeks after ZR, ZR:*Cox-2*^−/−^ mice had significantly lower forestomach tumor incidence and multiplicity than ZD:*Cox-2*^−/−^ mice (Fig. 5*a*). In addition, *S100a8*/*a9* mRNA expression was significantly lower in ZR:*Cox-2*^−/−^*vs*. ZD:*Cox-2*^−/−^ forestomach (Fig. 5*b*); S100A8/A9 protein expression was absent in ZR:*Cox-2*^−/−^ but strongly expressed in ZD:*Cox-2*^−/−^ forestomach (Fig. 5*c*). Histopathologic and IHC studies show that ZR:*Cox-2*^−/−^ forestomach mucosa was typically thin, with PCNA-positive nuclei mainly in basal cells and weak to negligible immunostaining of the 4 inflammation-associated markers S100A8, S100A9, p53, and cyclin D1 (Fig. 5*d*). In contrast, neoplastic ZD:*Cox-2*^−/−^ forestomach was highly proliferative, with PCNA-positive nuclei in many cell layers and strong overexpression of the same inflammation-associated markers (Fig. 5*d*). Thus, ZR reverses preneoplasia (Figs. 3*b*–3*d*), and effectively restores the antitumor effect of *Cox-2* ablation (Fig. 5) by attenuating the inflammation.

## Discussion

Increasingly cancers are treated with drugs that target specific pathways shown to be of pathogenetic significance. Our study shows that the antitumor effect of genetic disruption of *Cox-2* in tongue cancer prevention is bypassed by Zn depletion (Fig. 1), owing to activation of an alternative proneoplastic pathway that is not affected by COX-2 inhibition. Using a combination of techniques that included expression profiling, bioinformatics and investigation of identified markers in ZD:*Cox-2*^−/−^ mouse models of oral-esophageal cancers, our data document a mechanism for the inability of COX-2 blockade to prevent tumor growth under ZD conditions.

The hyperplastic ZD:*Cox-2*^−/−^*vs*. ZS:*Cox-2*^−/−^ forestomach has a distinct signature (Table 1). The pro-inflammation mediators *S100a8* and *S100a9* are upregulated 24-fold and 2.2-fold. In addition, the typical genes of the cornified envelope *Sprr2h*/*2f* and *Krt6A*/*16*/*17*/*8*/*20* are upregulated 64- to 5.5-fold. Because simultaneous upregulation of *S100A8*/*A9*, *SPRR2* and *KRT6A*/*16*/*17* is a common feature of human inflammatory skin diseases such as psoriasis^38^ and atopic dermatitis,^39^ this same signature in ZD:*Cox-2*^−/−^ forestomach indicates an association between inflammation and its highly hyperplastic phenotype. Our conclusion that *S100a8*/*a9* are relevant ZD-induced markers belonging to an inflammatory pathway that drives forestomach cell proliferation rather than an epiphenomenon of this process or of dietary Zn-deficit is supported by DAVID bioinformatics (Table 7*a*).

S100A8/A9 have emerged as important markers for inflammation-associated cancers.^32^,^40^ They are overexpressed in many human cancers,^32^ including lung, colorectal, prostate, skin cancer, as well as HPV18-infected oral SCC.^41^ The mechanistic role of S100A8/A9 in tumor biology is emerging. In a mouse skin cancer model, Gebhardt et al.^42^ provided genetic evidence that S100A8/A9 binds to RAGE, and RAGE signaling sustains skin inflammation and promotes tumorigenesis. In the lung, S100A8/A9 induces the activation of serum amyloid A that activates NF-κB inflammatory signaling and facilitates metastasis.^43^ In a colitis-induced mouse cancer model, S100A8/A9 and RAGE augment carcinogenesis^44^ and in an inflammation-associated liver cancer model, S100A8/A9 are identified as NF-κB target genes and their overexpression promotes malignant progression.^45^ Conversely, blockade of RAGE suppresses tumor growth and metastasis.^42^,^46^

Our IHC data in ZD:*Cox-2*^−/−^ tongue and ZD:*Cox-2*^+/−^ forestomach carcinomas (Fig. 4) demonstrate that under complete or partial blockade of COX-2 pathway dietary ZD activates an alternative cancer-associated RAGE-S100A8 inflammatory pathway. The finding that these same carcinomas showed high PCNA proliferative activity and prominent accumulation of p53 protein indicates that additional inflammation-associated cancer pathways are activated. The *p53* tumor suppressor gene is mutated in human oral and esophageal cancer.^36^ Mutated p53 protein has a prolonged half-life that leads to its accumulation in the nucleus. In this regard, human head and neck squamous cell cancer (HNSCC), which is a highly inflammatory, proliferative and aggressive cancer,^47^ exhibits high levels of p53 expression, abundant cell proliferative activity,^34^,^35^ as well as divergent carcinogenic pathways mediated separately by NF-κB and p53.^37^

Chronic inflammation contributes to the development of ∼20% of all human cancers. The causes of inflammation are often unknown.^48^ Our recent report in rat esophagus that dietary Zn regulates S100A8 expression and modulates the link between S100A8-RAGE and downstream NF-κB/COX-2 provides the first evidence that Zn has an inflammation-modulating role in essophageal cancer initiation/reversal.^14^ Here we demonstrate that with COX-2 pathway blockade prolonged dietary ZD causes chronic inflammation in the tongue/forestomach by activating alternative inflammatory RAGE-S100A8/A9 and p53 response pathways, thereby fueling tumor progression and bypassing the antitumor effect of *Cox-2* deletion. These new data provide a likely mechanism to explain the inefficacy of such targeted cancer therapy in oral-cancer patients, since many of these patients are frequently Zn-deficient.^4^–^7^

Recent studies reported that Zn supplementation improves clinical outcomes in patients receiving radiotherapy for HNSCC,^49^ as well as concomitant chemotherapy and radiotherapy for advanced nasopharyngeal carcinoma.^50^ The present finding that ZR attenuates the inflammatory response and restores the antitumor effect of COX-2 blockade has important clinical implications. Thus, stratification of patients by Zn status would be useful, and a personalized cancer therapeutic paradigm that includes Zn may improve efficacy.

## Abbreviations

COX-2: cyclooxygenase-2
DAVID: database for annotation, visualization and integrated discovery
GO: gene ontology
HNSCC: head and neck squamous cell cancer
IHC: immunohistochemistry
IPA: ingenuity pathway analysis
NMBA: *N*-nitrosomethylbenzylamine
NQO: 4-nitroquinoline 1-oxide
qRT-PCR: quantitative reverse transcriptase-polymerase chain reaction
RAGE: receptor for advanced glycation end products
S100a8: S100 calcium binding protein A8
S100a9: S100 calcium binding protein A9
SCC: squamous cell carcinoma
NF-κB: nuclear factor-κB
PCNA: proliferating cell nuclear antigen
Zn: zinc
ZD: Zn-deficiency
ZR: Zn-replenishment
ZS: Zn-sufficiency
