# Supplementary figures and images for: Zinc deficiency activates S100A8 inflammation in the absence of COX-2 and promotes murine oral-esophageal tumor progression
Source: Int J Cancer. 2010 Sep 20;129(2):331–45. doi: 10.1002/ijc.25688 (PMC3015018; doi:10.1002/ijc.25688)

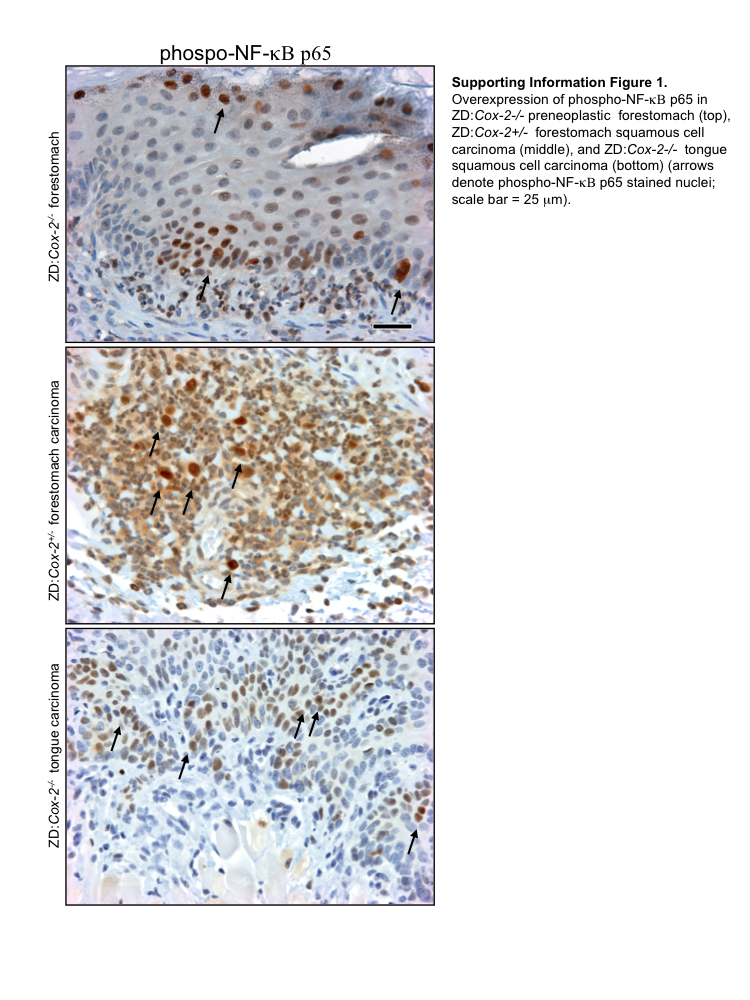

Supplement: Supplementary file 1 [file ijc0129-0331-SD1.tif]
