# Supplementary material for: Zinc deficiency activates S100A8 inflammation in the absence of COX-2 and promotes murine oral-esophageal tumor progression
Source: Int J Cancer. 2010 Sep 20;129(2):331–45. doi: 10.1002/ijc.25688 (PMC3015018; doi:10.1002/ijc.25688)
Supplement: Supplementary file 2 [file ijc0129-0331-SD2.doc]

**Supporting Information** **Table 1.** Incidence and tumor multiplicity of tongue, esophageal, and forestomach tumors in

*Cox-2* deficient mice after 26 weeks of treatment with low doses of 4-nitroquinoline 1-oxide (NQO)*

**a) Tumor/Carcinoma incidence**

| Mouse group  Diet : genotype | Incidence (%) | | | | | |
| --- | --- | --- | --- | --- | --- | --- |
|  | Tongue | | Esophagus | | Forestomach | |
|  | Tumor | Carcinoma | Tumor | Carcinoma | Tumor | Carcinoma |
| ZD:*Cox-2*^-/-^ | 14/14 (100.0) | 5/14 (35.7) | 6/14 (42.8) | 2/14 (14.3) | 13/14 (92.8) | 2/14 (14.3) |
| ZD:*Cox-2*^+/-^ | 36/46 (78.3) | 15/46 (32.6) | 13/46 (28.3) | 2/46 (4.4) | 37/46 (80.4) | 5/46 (10.9) |
| ZD:WT | 13/19 (68.4) | 0/19 (0.0) | 2/19 (10.5) | 0/19 (0.0) | 12/19 (63.1) | 1/19 (5.3) |
| ZS:*Cox-2*^-/-^ | 4/16 (25.0) | 0/16 (0.0) | 0/16 (0.0) | 0/16 (0.0) | 3/16 (18.8) | 0/16 (0.0) |
| ZS:*Cox-2*^+/-^ | 10/37 (27.0) | 0/37 (0.0) | 0/37 (0.0) | 0/37 (0.0) | 7/37 (18.9) | 0/37 (0.0) |
| ZS:WT | 15/25 (60.0) | 0/25 (0.0) | 2/25 (8.0) | 0/25 (0.0) | 14/25 (56.0) | 0/25 (0.0) |

| Mouse group comparison | Tongue | | | | Esophagus | | | | Forestomach | | | |
| --- | --- | --- | --- | --- | --- | --- | --- | --- | --- | --- | --- | --- |
|  | Tumor | | Carcinoma | | Tumor | | Carcinoma | | Tumor | | Carcinoma | |
|  | % Difference (95% CI) | *P*-value | % Difference (95% CI) | *P*-value | % Difference  (95% CI) | *P*-value | % Difference  (95% CI) | *P*-value | % Difference  (95% CI) | *P*-value | % Difference  (95% CI) | *P*-value |
| ZD:*Cox-2*^-/-^ *vs* ZD:WT | 31.6  (4.6 to 54.0) | 0.027 | 35.7  (10.1 to 61.2) | 0.008 | 32.3  (2.4 to 58.0) | 0.047 | 14.3  (-5.4 to 4.0) | NS | 29.7  (-0.4 to 52.6) | NS | 9.0  (-12.9 to 35.0) | NS |
| ZD:*Cox-2*^+/-^ *vs* ZD:WT | 9.8  (-11.5 to 34.2) | NS | 32.6  (12.1 to 4.07) | 0.003 | 17.7  (-5.8 to 33.9) | NS | 4.4  (-12.8 to 14.5) | NS | 17.3  (-5.0 to 41.1) | NS | 5.6  (-14.7 to 18.5) | NS |
| ZD:*Cox-2*^-/-^ *vs* ZD:*Cox-2*^+/-^ | 21.7  (-1.8 to 35.6) | NS | 3.1  (-21.0 to 31.2) | NS | -14.6  (-41.5 to 11.2) | NS | 9.9  (-4.5 to 35.8) | NS | 12.4  (-13.5 to 27.2) | NS | -3.4  (-29.8 to 12.5) | NS |
| ZS:*Cox-2*^-/-^ *vs* ZS:WT | -35.0  (-3.8 to -57.3) | 0.05 | 0.0  (-13.3 to 19.4) | NS | -8.0  (-25.0 to 1.2) | NS | 0.0  (-13.3 to 19.4) | NS | -37.3  (-58.4 to 6.5) | .025 | 0.0  (-13.3 to 19.4) | NS |
| ZS:COX-2^+/-^*vs* ZS:WT | -33.0  (8.0 to 53.2) | 0.03 | 0.0  (-13.4 to 9.4) | NS | -8.0  (-25.0 to 3.0) | NS | 0.0  (-13.3 to 9.4) | NS | -37.1  (-56.8 to 12.8) | .0005 | 0.0  (-13.3 to 9.4) | NS |
| ZS:*Cox-2*^-/-^ *vs* ZS:*Cox-2*^+/-^ | 2.0  (-25.0 to 23.8) | NS | 0.0  (-9.4 to 19.4) | NS | 0.0  (-9.4 to 19.4) | NS | 0.0  (-9.4 to 19.4) | NS | 0.2  (-25.6 to 19.7) | NS | 0.0  (-19.4 to 9.4) | NS |
| ZD:*Cox-2*^-/-^*vs* ZS:*Cox-2*^-/-^ | 75.0  (42.4 to 89.8) | .00002 | 35.7  (8.3 to 61.2) | 0.01 | 42.9  (13.9 to 67.4) | 0.005 | 14.3  (-7.6 to 39.9) | NS | 74.1  (39.8 to 87.7) | .000056 | 14.3  (-7.6 to 39.9) | NS |
| ZD:*Cox-2*^+/-^*vs* ZS:*Cox-2*^+/-^ | 51.2  (30.1 to 66.2) | .000006 | 32.6  (17.6 to 47.0) | 0.00007 | 28.3  (13.8 to 42.6) | 0.00036 | 4.4  (-5.6 to 14.5) | NS | 61.5  (41.1 to 74.5) | .0000002 | 10.9  (-0.4 to 20.3) | .06 |
| ZD:WT *vs* ZS:WT | 8.4  (-19.5 to 33.6) | NS | 0.0  (-13.3 to 16.8) | NS | 2.5  (-16.1 to 24.2) | NS | 0.0  (-13.3 to 16.8) | NS | 7.2  (-20.1 to 33.1) | NS | 5.3  (-8.7 to 24.6) | NS |

**b) Tumor multiplicity**

| Mouse group  Diet : genotype | Number of tumors/site (95% CI) | | |
| --- | --- | --- | --- |
|  | Tongue | Esophagus | Forestomach |
| ZD:*Cox-2*^-/-^ | 7.57 (4.69 to 10.45) | 1.71 (0.29 to 3.14) | 6.07 (3.23 to 8.91) |
| ZD:*Cox-2*^+/-^ | 4.97 (3.48 to 6.46) | 1.23 (0.56 to 1.89) | 4.13 (2.46 to 5.80) |
| ZD:WT | 1.42 (0.77 to 2.07) | 0.11 ( -0.05 to 0.26) | 1.74 (0.29 to 3.18) |
| ZS:*Cox-2*^-/-^ | 0.56 (0.01 to 1.11) | 0.00 (0.00 to 0.00) | 0.38 (-0.05 to 0.80) |
| ZS:*Cox-2*^+/-^ | 0.81 (0.35 to 1.28) | 0.00 (0.00 to 0.00) | 0.44 (0.11 to 0.78) |
| ZS:WT | 1.72 (1.08 to 2.36) | 0.00 (0.00 to 0.00) | 2.00 (1.06 to 2.93) |

| Mouse group  comparison | Tongue | | Esophagus | | Forestomach | |
| --- | --- | --- | --- | --- | --- | --- |
|  | Difference  (95% CI) | *P*-value | Difference  (95% CI) | *P*-value | Difference  (95% CI) | *P*-value |
| ZD:*Cox-2*^-/-^ *vs* ZD:WT | 6.15  (3.33 to 8.97) | <.00001 | 1.61  (0.40 to 2.82) | .0026 | 4.33  (1.13 to 7.54) | .00204 |
| ZD:*Cox-2*^+/-^ *vs* ZD:WT | 3.55  (1.22 to 5.88) | .0003 | 1.12  (0.12 to 2.12) | .0190 | 2.30  (-0.26 to 5.05) | NS |
| ZS:*Cox-2*^-/-^ *vs* ZS:WT | -1.15  (-3.72 to 1.32) | NS | 0.00  (-1.10 to 1.10) | NS | -1.63  (-4.54 to 1.29) | NS |
| ZS:COX-2^+/-^ *vs* ZS:WT | -0.91  (-3.12 to 1.31) | NS | 0.00  (-0.95 to 0.95) | NS | -1.55  (-4.08 to 0.97) | NS |
| ZD:*Cox-2*^-/-^ *vs* ZS:*Cox-2*^-/-^ | 7.01  (4.09 to 9.94) | <.00001 | 1.71  (0.46 to 2.97) | .0018 | 5.69  (2.36 to 9.03) | .00003 |
| ZD:*Cox-2*^+/-^ *vs* ZS:*Cox-2*^+/-^ | 4.15  (2.05 to 6.26) | <.00001 | 1.22  (0.32 to 2.13) | .0020 | 3.68  (1.29 to 6.08) | .00027 |
| ZD:WT *vs* ZS:WT | -0.30  (-2.73 to 2.13) | NS | 0.11  (-0.94 to 1.15) | NS | -0.26  (-3.04 to 2.51) | NS |

Tumor incidence (number of mice with tumors/total number of mice) was compared by two-tailed Fisher’s exact test.

Tumor multiplicity (number of tumors per site) was compared by two-way ANOVA and the TukeyHSD post hoc multiple

comparison test. CI = confidence intervals; NS = not significant. All statistical tests were two-sided.

ZD = zinc-deficient; ZS = zinc-sufficient; WT = wild-type;
