# Supplementary material for: Zinc deficiency activates S100A8 inflammation in the absence of COX-2 and promotes murine oral-esophageal tumor progression
Source: Int J Cancer. 2010 Sep 20;129(2):331–45. doi: 10.1002/ijc.25688 (PMC3015018; doi:10.1002/ijc.25688)
Supplement: Supplementary file 3 [file ijc0129-0331-SD3.doc]

**Supporting Information Table 2.** Gene expression profile analysis of ZD:*Cox-2^-/-^* *vs* ZS:*Cox-2^-/-^* mouse forestomach

**Description of the problem:**

Number of classes: 2

Number of genes used for random variance estimation: 45101

Number of genes that passed filtering criteria: 45101

Type of univariate test used: Two-sample T-test (with random variance model)

Column of the Experiment Descriptors sheet that defines class variable : **ZD-- vs ZS-- (ZD:*Cox-2^-/-^* vs ZS:*Cox-2^-/-^*)**

ZD = zinc-deficient; ZS = zinc-sufficient

Univariate test random variance model parameters: a= 1.74478 , b= 20.8714 , Kolmogorov-Smirnov statistic= 0.02329

Nominal significance level of each univariate test: 0.05

**Summary of Results:**

**Number of genes significant at 0.05 level of the univariate test:** **6007**

**Number of genes significant at 0.05 level and with a cut-off point of 2-fold or more difference: 314**

**Genes which discriminate among classes:**

Table- Sorted by p-value of the univariate test and a cut-off point of 2-fold or more difference

Class 1: *ZD--*; Class 2: *ZS--*.

**Up-regulated genes are in purple, down-regulated genes are in blue (242 up-regulated and 72 down-regulated)**

| **p-value** | **FDR** | **ZD--** | **ZS--** | **Fold-change** | **Probe set** | **Gene symbol** | **Description** |
| --- | --- | --- | --- | --- | --- | --- | --- |
| 5.11E-05 | 0.166874 | 1776.541548 | 27.7377094 | 64 | [1422240_s_at](https://www.affymetrix.com/LinkServlet?probeset=1422240_s_at) | [Sprr2h](http://www.ncbi.nlm.nih.gov/entrez/query.fcgi?cmd=search&db=gene&term=Sprr2h) | small proline-rich protein 2H |
| 9.29E-05 | 0.207465 | 1256.721397 | 25.4984332 | 49 | [1449833_at](https://www.affymetrix.com/LinkServlet?probeset=1449833_at) | [Sprr2f](http://www.ncbi.nlm.nih.gov/entrez/query.fcgi?cmd=search&db=gene&term=Sprr2f) | small proline-rich protein 2F |
| 6.14E-05 | 0.166874 | 2810.089093 | 59.2098351 | 47 | [1448932_at](https://www.affymetrix.com/LinkServlet?probeset=1448932_at) | [Krt16](http://www.ncbi.nlm.nih.gov/entrez/query.fcgi?cmd=search&db=gene&term=Krt16) | keratin 16 |
| 0.000556 | 0.26452 | 3729.645681 | 82.6872933 | 45 | [1422784_at](https://www.affymetrix.com/LinkServlet?probeset=1422784_at) | [Krt6a](http://www.ncbi.nlm.nih.gov/entrez/query.fcgi?cmd=search&db=gene&term=Krt6a) | keratin 6A |
| 0.00431 | 0.277913 | 9735.236495 | 249.1760508 | 39 | [1425450_at](https://www.affymetrix.com/LinkServlet?probeset=1425450_at) | [Chi3l4](http://www.ncbi.nlm.nih.gov/entrez/query.fcgi?cmd=search&db=gene&term=Chi3l4) | chitinase 3-like 4 |
| 0.00504 | 0.280839 | 13668.41459 | 408.0123188 | 34 | [1425451_s_at](https://www.affymetrix.com/LinkServlet?probeset=1425451_s_at) | [Chi3l4](http://www.ncbi.nlm.nih.gov/entrez/query.fcgi?cmd=search&db=gene&term=Chi3l4) | chitinase 3-like 4 |
| 0.000546 | 0.26452 | 3460.497951 | 112.3159686 | 31 | [1422783_a_at](https://www.affymetrix.com/LinkServlet?probeset=1422783_a_at) | [Krt6a](http://www.ncbi.nlm.nih.gov/entrez/query.fcgi?cmd=search&db=gene&term=Krt6a) | keratin 6A |
| 5.00E-07 | 0.022551 | 1113.176184 | 38.3596466 | 29 | [1421806_at](https://www.affymetrix.com/LinkServlet?probeset=1421806_at) | [Defb3](http://www.ncbi.nlm.nih.gov/entrez/query.fcgi?cmd=search&db=gene&term=Defb3) | defensin beta 3 |
| 0.00015 | 0.229386 | 814.0363262 | 29.773472 | 27 | [1418287_a_at](https://www.affymetrix.com/LinkServlet?probeset=1418287_a_at) | [Dmbt1](http://www.ncbi.nlm.nih.gov/entrez/query.fcgi?cmd=search&db=gene&term=Dmbt1) | deleted in malignant brain tumors 1 |
| 4.20E-06 | 0.063141 | 1377.6478 | 58.6347992 | 24 | [1419394_s_at](https://www.affymetrix.com/LinkServlet?probeset=1419394_s_at) | [S100a8](http://www.ncbi.nlm.nih.gov/entrez/query.fcgi?cmd=search&db=gene&term=S100a8) | S100 calcium binding protein A8 (calgranulin A) |
| 0.000714 | 0.26452 | 280.7755928 | 12.398424 | 23 | [1449989_at](https://www.affymetrix.com/LinkServlet?probeset=1449989_at) | [Mcpt2](http://www.ncbi.nlm.nih.gov/entrez/query.fcgi?cmd=search&db=gene&term=Mcpt2) | mast cell protease 2 |
| 0.002588 | 0.267906 | 459.7544245 | 26.7559633 | 17 | [1450060_at](https://www.affymetrix.com/LinkServlet?probeset=1450060_at) | [Pigr](http://www.ncbi.nlm.nih.gov/entrez/query.fcgi?cmd=search&db=gene&term=Pigr) | polymeric immunoglobulin receptor |
| 0.003087 | 0.276536 | 651.9076773 | 38.2098384 | 17 | [1423719_at](https://www.affymetrix.com/LinkServlet?probeset=1423719_at) | [U46068](http://www.ncbi.nlm.nih.gov/entrez/query.fcgi?cmd=search&db=gene&term=U46068) | cDNA sequence U46068 |
| 0.000963 | 0.26452 | 204.6108358 | 12.4346465 | 17 | [1419764_at](https://www.affymetrix.com/LinkServlet?probeset=1419764_at) | [Chi3l3](http://www.ncbi.nlm.nih.gov/entrez/query.fcgi?cmd=search&db=gene&term=Chi3l3) | chitinase 3-like 3 |
| 0.000956 | 0.26452 | 781.1210981 | 51.572087 | 15 | [1450009_at](https://www.affymetrix.com/LinkServlet?probeset=1450009_at) | [Ltf](http://www.ncbi.nlm.nih.gov/entrez/query.fcgi?cmd=search&db=gene&term=Ltf) | lactotransferrin |
| 0.000594 | 0.26452 | 1199.751909 | 81.9041024 | 15 | [1427700_x_at](https://www.affymetrix.com/LinkServlet?probeset=1427700_x_at) | [Krt6a](http://www.ncbi.nlm.nih.gov/entrez/query.fcgi?cmd=search&db=gene&term=Krt6a) | keratin 6A |
| 0.00028 | 0.257465 | 1148.191862 | 81.2823165 | 14 | [1431213_a_at](https://www.affymetrix.com/LinkServlet?probeset=1431213_a_at) | [LOC67527](http://www.ncbi.nlm.nih.gov/entrez/query.fcgi?cmd=search&db=gene&term=LOC67527) | murine leukemia retrovirus |
| 0.000433 | 0.26452 | 108.8368216 | 8.0315598 | 14 | [1422352_at](https://www.affymetrix.com/LinkServlet?probeset=1422352_at) | [Mcpt1](http://www.ncbi.nlm.nih.gov/entrez/query.fcgi?cmd=search&db=gene&term=Mcpt1) | mast cell protease 1 |
| 0.000419 | 0.26452 | 578.6935083 | 45.0962707 | 13 | [1428909_at](https://www.affymetrix.com/LinkServlet?probeset=1428909_at) | [A130040M12Rik](http://www.ncbi.nlm.nih.gov/entrez/query.fcgi?cmd=search&db=gene&term=A130040M12Rik) | RIKEN cDNA A130040M12 gene |
| 0.004026 | 0.276588 | 694.7817162 | 57.672391 | 12 | [1439423_x_at](https://www.affymetrix.com/LinkServlet?probeset=1439423_x_at) | [U46068](http://www.ncbi.nlm.nih.gov/entrez/query.fcgi?cmd=search&db=gene&term=U46068) | cDNA sequence U46068 |
| 0.003096 | 0.276536 | 557.9724733 | 46.3535794 | 12 | [1429286_at](https://www.affymetrix.com/LinkServlet?probeset=1429286_at) | [1190003M12Rik](http://www.ncbi.nlm.nih.gov/entrez/query.fcgi?cmd=search&db=gene&term=1190003M12Rik) | RIKEN cDNA 1190003M12 gene |
| 0.009845 | 0.304874 | 1112.112285 | 93.5081338 | 12 | [1422448_at](https://www.affymetrix.com/LinkServlet?probeset=1422448_at) | [Tff2](http://www.ncbi.nlm.nih.gov/entrez/query.fcgi?cmd=search&db=gene&term=Tff2) | trefoil factor 2 (spasmolytic protein 1) |
| 0.001004 | 0.26452 | 703.6904371 | 65.7386454 | 11 | [1423227_at](https://www.affymetrix.com/LinkServlet?probeset=1423227_at) | [Krt17](http://www.ncbi.nlm.nih.gov/entrez/query.fcgi?cmd=search&db=gene&term=Krt17) | keratin 17 |
| 0.003596 | 0.276588 | 350.5637891 | 34.363791 | 10 | [1455490_at](https://www.affymetrix.com/LinkServlet?probeset=1455490_at) | [Pigr](http://www.ncbi.nlm.nih.gov/entrez/query.fcgi?cmd=search&db=gene&term=Pigr) | polymeric immunoglobulin receptor |
| 0.002416 | 0.26452 | 386.6587841 | 38.815427 | 10 | [1419268_at](https://www.affymetrix.com/LinkServlet?probeset=1419268_at) | [Agr2](http://www.ncbi.nlm.nih.gov/entrez/query.fcgi?cmd=search&db=gene&term=Agr2) | anterior gradient 2 (Xenopus laevis) |
| 0.003167 | 0.276588 | 175.6168838 | 18.9223757 | 9.3 | [1454264_at](https://www.affymetrix.com/LinkServlet?probeset=1454264_at) | [2310046K23Rik](http://www.ncbi.nlm.nih.gov/entrez/query.fcgi?cmd=search&db=gene&term=2310046K23Rik) | RIKEN cDNA 2310046K23 gene |
| 0.008784 | 0.302035 | 920.0290698 | 103.8427877 | 8.9 | [1417156_at](https://www.affymetrix.com/LinkServlet?probeset=1417156_at) | [Krt19](http://www.ncbi.nlm.nih.gov/entrez/query.fcgi?cmd=search&db=gene&term=Krt19) | keratin 19 |
| 0.013349 | 0.315296 | 69.1219067 | 8.3666951 | 8.3 | [1416306_at](https://www.affymetrix.com/LinkServlet?probeset=1416306_at) | [Clca3](http://www.ncbi.nlm.nih.gov/entrez/query.fcgi?cmd=search&db=gene&term=Clca3) | chloride channel calcium activated 3 |
| 0.03486 | 0.349697 | 760.8975862 | 92.6096172 | 8.2 | [1449426_a_at](https://www.affymetrix.com/LinkServlet?probeset=1449426_a_at) | [Anxa10](http://www.ncbi.nlm.nih.gov/entrez/query.fcgi?cmd=search&db=gene&term=Anxa10) | annexin A10 |
| 0.001523 | 0.26452 | 265.6155126 | 32.8401755 | 8.1 | [1419600_at](https://www.affymetrix.com/LinkServlet?probeset=1419600_at) | [Defb4](http://www.ncbi.nlm.nih.gov/entrez/query.fcgi?cmd=search&db=gene&term=Defb4) | defensin beta 4 |
| 0.003544 | 0.276588 | 617.4611447 | 77.095581 | 8.0 | [1438648_x_at](https://www.affymetrix.com/LinkServlet?probeset=1438648_x_at) | [1190003M12Rik](http://www.ncbi.nlm.nih.gov/entrez/query.fcgi?cmd=search&db=gene&term=1190003M12Rik) | RIKEN cDNA 1190003M12 gene |
| 0.00228 | 0.26452 | 2069.546301 | 259.6776049 | 8.0 | [1435761_at](https://www.affymetrix.com/LinkServlet?probeset=1435761_at) | [Stfa1](http://www.ncbi.nlm.nih.gov/entrez/query.fcgi?cmd=search&db=gene&term=Stfa1) | stefin A1 |
| 0.001924 | 0.26452 | 308.7244244 | 38.9372194 | 7.9 | [1418724_at](https://www.affymetrix.com/LinkServlet?probeset=1418724_at) | [Cfi](http://www.ncbi.nlm.nih.gov/entrez/query.fcgi?cmd=search&db=gene&term=Cfi) | complement component factor i |
| 0.000219 | 0.229386 | 76.9805797 | 9.7419853 | 7.9 | [1449254_at](https://www.affymetrix.com/LinkServlet?probeset=1449254_at) | [Spp1](http://www.ncbi.nlm.nih.gov/entrez/query.fcgi?cmd=search&db=gene&term=Spp1) | secreted phosphoprotein 1 |
| 0.00374 | 0.276588 | 529.3296391 | 67.0047817 | 7.9 | [1448169_at](https://www.affymetrix.com/LinkServlet?probeset=1448169_at) | [Krt18](http://www.ncbi.nlm.nih.gov/entrez/query.fcgi?cmd=search&db=gene&term=Krt18) | keratin 18 |
| 0.010737 | 0.305162 | 931.0900381 | 131.3498429 | 7.1 | [1420647_a_at](https://www.affymetrix.com/LinkServlet?probeset=1420647_a_at) | [Krt8](http://www.ncbi.nlm.nih.gov/entrez/query.fcgi?cmd=search&db=gene&term=Krt8) | keratin 8 |
| 0.010963 | 0.305831 | 994.1158283 | 144.2899736 | 7.0 | [1423691_x_at](https://www.affymetrix.com/LinkServlet?probeset=1423691_x_at) | [Krt8](http://www.ncbi.nlm.nih.gov/entrez/query.fcgi?cmd=search&db=gene&term=Krt8) | keratin 8 |
| 0.021596 | 0.327657 | 47.373869 | 6.9216798 | 6.8 | [1440852_at](https://www.affymetrix.com/LinkServlet?probeset=1440852_at) | [Idi2](http://www.ncbi.nlm.nih.gov/entrez/query.fcgi?cmd=search&db=gene&term=Idi2) | isopentenyl-diphosphate delta isomerase 2 |
| 0.000694 | 0.26452 | 428.7723033 | 62.7929516 | 6.8 | [1431214_at](https://www.affymetrix.com/LinkServlet?probeset=1431214_at) | [LOC67527](http://www.ncbi.nlm.nih.gov/entrez/query.fcgi?cmd=search&db=gene&term=LOC67527) | murine leukemia retrovirus |
| 0.008342 | 0.300016 | 903.1535695 | 134.1433496 | 6.7 | [1435989_x_at](https://www.affymetrix.com/LinkServlet?probeset=1435989_x_at) | [Krt8](http://www.ncbi.nlm.nih.gov/entrez/query.fcgi?cmd=search&db=gene&term=Krt8) | keratin 8 |
| 0.001335 | 0.26452 | 243.9629926 | 36.4281553 | 6.7 | [1416325_at](https://www.affymetrix.com/LinkServlet?probeset=1416325_at) | [Crisp1](http://www.ncbi.nlm.nih.gov/entrez/query.fcgi?cmd=search&db=gene&term=Crisp1) | cysteine-rich secretory protein 1 |
| 0.002358 | 0.26452 | 73.1812388 | 11.1235764 | 6.6 | [1420579_s_at](https://www.affymetrix.com/LinkServlet?probeset=1420579_s_at) | [Cftr](http://www.ncbi.nlm.nih.gov/entrez/query.fcgi?cmd=search&db=gene&term=Cftr) | cystic fibrosis transmembrane conductance regulator homolog |
| 0.003868 | 0.276588 | 197.7796959 | 33.0419758 | 6.0 | [1421653_a_at](https://www.affymetrix.com/LinkServlet?probeset=1421653_a_at) | [Igh](http://www.ncbi.nlm.nih.gov/entrez/query.fcgi?cmd=search&db=gene&term=Igh) | immunoglobulin heavy chain complex |
| 0.021292 | 0.327626 | 226.1992527 | 38.0802949 | 5.9 | [1424649_a_at](https://www.affymetrix.com/LinkServlet?probeset=1424649_a_at) | [Tspan8](http://www.ncbi.nlm.nih.gov/entrez/query.fcgi?cmd=search&db=gene&term=Tspan8) | tetraspanin 8 |
| 0.03346 | 0.346799 | 1061.461443 | 179.9926108 | 5.9 | [1418989_at](https://www.affymetrix.com/LinkServlet?probeset=1418989_at) | [Ctse](http://www.ncbi.nlm.nih.gov/entrez/query.fcgi?cmd=search&db=gene&term=Ctse) | cathepsin E |
| 0.000359 | 0.264306 | 1181.397348 | 201.0363191 | 5. 9 | [1421156_a_at](https://www.affymetrix.com/LinkServlet?probeset=1421156_a_at) | [Dsc2](http://www.ncbi.nlm.nih.gov/entrez/query.fcgi?cmd=search&db=gene&term=Dsc2) | desmocollin 2 |
| 0.005554 | 0.282385 | 219.0604861 | 37.9633035 | 5.8 | [1435639_at](https://www.affymetrix.com/LinkServlet?probeset=1435639_at) | [2610528A11Rik](http://www.ncbi.nlm.nih.gov/entrez/query.fcgi?cmd=search&db=gene&term=2610528A11Rik) | RIKEN cDNA 2610528A11 gene |
| 0.005244 | 0.280839 | 220.2838159 | 38.5866343 | 5.7 | [1425763_x_at](https://www.affymetrix.com/LinkServlet?probeset=1425763_x_at) | [Igh](http://www.ncbi.nlm.nih.gov/entrez/query.fcgi?cmd=search&db=gene&term=Igh) | immunoglobulin heavy chain complex |
| 0.0002 | 0.229386 | 365.0298774 | 65.3526854 | 5.6 | [1421551_s_at](https://www.affymetrix.com/LinkServlet?probeset=1421551_s_at) | [Ifi202b](http://www.ncbi.nlm.nih.gov/entrez/query.fcgi?cmd=search&db=gene&term=Ifi202b) | interferon activated gene 202B |
| 0.005029 | 0.280839 | 34.3969648 | 6.2757371 | 5.5 | [1415837_at](https://www.affymetrix.com/LinkServlet?probeset=1415837_at) | [Klk1](http://www.ncbi.nlm.nih.gov/entrez/query.fcgi?cmd=search&db=gene&term=Klk1) | kallikrein 1 |
| 0.022077 | 0.32853 | 187.1025466 | 34.2664474 | 5.5 | [1448980_at](https://www.affymetrix.com/LinkServlet?probeset=1448980_at) | [Ghrl](http://www.ncbi.nlm.nih.gov/entrez/query.fcgi?cmd=search&db=gene&term=Ghrl) | ghrelin |
| 0.007501 | 0.294206 | 161.7277644 | 29.6854692 | 5.4 | [1426284_at](https://www.affymetrix.com/LinkServlet?probeset=1426284_at) | [Krt20](http://www.ncbi.nlm.nih.gov/entrez/query.fcgi?cmd=search&db=gene&term=Krt20) | keratin 20 |
| 0.01574 | 0.320151 | 100.4983688 | 18.6529814 | 5.4 | [1440409_at](https://www.affymetrix.com/LinkServlet?probeset=1440409_at) | [Gcnt3](http://www.ncbi.nlm.nih.gov/entrez/query.fcgi?cmd=search&db=gene&term=Gcnt3) | glucosaminyl (N-acetyl) transferase 3, mucin type |
| 0.002291 | 0.26452 | 131.0310541 | 24.4955408 | 5.3 | [1417231_at](https://www.affymetrix.com/LinkServlet?probeset=1417231_at) | [Cldn2](http://www.ncbi.nlm.nih.gov/entrez/query.fcgi?cmd=search&db=gene&term=Cldn2) | claudin 2 |
| 0.010376 | 0.305162 | 38.8177925 | 7.7992719 | 5.0 | [1430443_at](https://www.affymetrix.com/LinkServlet?probeset=1430443_at) | [Anxa10](http://www.ncbi.nlm.nih.gov/entrez/query.fcgi?cmd=search&db=gene&term=Anxa10) | annexin A10 |
| 0.00018 | 0.229386 | 86.3456397 | 17.8604929 | 4.8 | [1448393_at](https://www.affymetrix.com/LinkServlet?probeset=1448393_at) | [Cldn7](http://www.ncbi.nlm.nih.gov/entrez/query.fcgi?cmd=search&db=gene&term=Cldn7) | claudin 7 |
| 0.000369 | 0.264306 | 362.9550225 | 76.0757996 | 4.8 | [1457666_s_at](https://www.affymetrix.com/LinkServlet?probeset=1457666_s_at) | [Ifi202b](http://www.ncbi.nlm.nih.gov/entrez/query.fcgi?cmd=search&db=gene&term=Ifi202b) | interferon activated gene 202B |
| 0.000522 | 0.26452 | 464.4125221 | 98.2105075 | 4.7 | [1426911_at](https://www.affymetrix.com/LinkServlet?probeset=1426911_at) | [Dsc2](http://www.ncbi.nlm.nih.gov/entrez/query.fcgi?cmd=search&db=gene&term=Dsc2) | desmocollin 2 |
| 0.044036 | 0.364609 | 241.1393809 | 51.2372795 | 4.7 | [1449428_at](https://www.affymetrix.com/LinkServlet?probeset=1449428_at) | [Cldn18](http://www.ncbi.nlm.nih.gov/entrez/query.fcgi?cmd=search&db=gene&term=Cldn18) | claudin 18 |
| 3.11E-05 | 0.161134 | 811.3554576 | 173.5654164 | 4.7 | [1435137_s_at](https://www.affymetrix.com/LinkServlet?probeset=1435137_s_at) | [1200016E24Rik](http://www.ncbi.nlm.nih.gov/entrez/query.fcgi?cmd=search&db=gene&term=1200016E24Rik) | RIKEN cDNA 1200016E24 gene |
| 0.001431 | 0.26452 | 6515.745102 | 1410.891544 | 4.6 | [1422588_at](https://www.affymetrix.com/LinkServlet?probeset=1422588_at) | [Krt6b](http://www.ncbi.nlm.nih.gov/entrez/query.fcgi?cmd=search&db=gene&term=Krt6b) | keratin 6B |
| 0.001403 | 0.26452 | 232.7958222 | 50.7843384 | 4.6 | [1425538_x_at](https://www.affymetrix.com/LinkServlet?probeset=1425538_x_at) | [Ceacam1](http://www.ncbi.nlm.nih.gov/entrez/query.fcgi?cmd=search&db=gene&term=Ceacam1) | carcinoembryonic antigen-related cell adhesion molecule 1 |
| 0.010526 | 0.305162 | 38.5329966 | 8.4908393 | 4.5 | [1448107_x_at](https://www.affymetrix.com/LinkServlet?probeset=1448107_x_at) | [Klk1](http://www.ncbi.nlm.nih.gov/entrez/query.fcgi?cmd=search&db=gene&term=Klk1) | kallikrein 1 |
| 1.21E-05 | 0.136431 | 1740.46619 | 384.4439936 | 4.5 | [1427932_s_at](https://www.affymetrix.com/LinkServlet?probeset=1427932_s_at) | [1200016E24Rik](http://www.ncbi.nlm.nih.gov/entrez/query.fcgi?cmd=search&db=gene&term=1200016E24Rik) | RIKEN cDNA 1200016E24 gene |
| 0.00166 | 0.26452 | 127.0322072 | 28.0621598 | 4.5 | [1415824_at](https://www.affymetrix.com/LinkServlet?probeset=1415824_at) | [Scd2](http://www.ncbi.nlm.nih.gov/entrez/query.fcgi?cmd=search&db=gene&term=Scd2) | stearoyl-Coenzyme A desaturase 2 |
| 0.014959 | 0.316889 | 418.8080476 | 94.5015203 | 4.4 | [1449199_at](https://www.affymetrix.com/LinkServlet?probeset=1449199_at) | [Muc1](http://www.ncbi.nlm.nih.gov/entrez/query.fcgi?cmd=search&db=gene&term=Muc1) | mucin 1, transmembrane |
| 0.0263 | 0.339666 | 41.1882372 | 9.4184987 | 4.4 | [1427747_a_at](https://www.affymetrix.com/LinkServlet?probeset=1427747_a_at) | [Lcn2](http://www.ncbi.nlm.nih.gov/entrez/query.fcgi?cmd=search&db=gene&term=Lcn2) | lipocalin 2 |
| 0.01526 | 0.318295 | 413.8812364 | 96.5104315 | 4.3 | [1419700_a_at](https://www.affymetrix.com/LinkServlet?probeset=1419700_a_at) | [Prom1](http://www.ncbi.nlm.nih.gov/entrez/query.fcgi?cmd=search&db=gene&term=Prom1) | prominin 1 |
| 0.004534 | 0.277913 | 90.2798309 | 21.1912768 | 4.3 | [1425445_a_at](https://www.affymetrix.com/LinkServlet?probeset=1425445_a_at) | [Cldn18](http://www.ncbi.nlm.nih.gov/entrez/query.fcgi?cmd=search&db=gene&term=Cldn18) | claudin 18 |
| 0.029235 | 0.341697 | 536.4859731 | 126.8977642 | 4.2 | [1417732_at](https://www.affymetrix.com/LinkServlet?probeset=1417732_at) | [Anxa8](http://www.ncbi.nlm.nih.gov/entrez/query.fcgi?cmd=search&db=gene&term=Anxa8) | annexin A8 |
| 0.004601 | 0.278162 | 123.7175736 | 29.4043064 | 4.2 | [1418818_at](https://www.affymetrix.com/LinkServlet?probeset=1418818_at) | [Aqp5](http://www.ncbi.nlm.nih.gov/entrez/query.fcgi?cmd=search&db=gene&term=Aqp5) | aquaporin 5 |
| 0.014024 | 0.316828 | 30.6819683 | 7.4874072 | 4.1 | [1438555_x_at](https://www.affymetrix.com/LinkServlet?probeset=1438555_x_at) | [Muc4](http://www.ncbi.nlm.nih.gov/entrez/query.fcgi?cmd=search&db=gene&term=Muc4) | mucin 4 |
| 0.000433 | 0.26452 | 52.9073639 | 13.0282977 | 4.1 | [1452205_x_at](https://www.affymetrix.com/LinkServlet?probeset=1452205_x_at) | [Tcrb-J](http://www.ncbi.nlm.nih.gov/entrez/query.fcgi?cmd=search&db=gene&term=Tcrb-J) | T-cell receptor beta, joining region |
| 0.016312 | 0.320151 | 144.9973063 | 35.7769478 | 4.1 | [1452417_x_at](https://www.affymetrix.com/LinkServlet?probeset=1452417_x_at) | [NA](http://www.ncbi.nlm.nih.gov/entrez/query.fcgi?cmd=search&db=gene&term=NA) | NA |
| 0.007255 | 0.293015 | 185.5149405 | 45.8111086 | 4.1 | [1426268_at](https://www.affymetrix.com/LinkServlet?probeset=1426268_at) | [C130090K23Rik](http://www.ncbi.nlm.nih.gov/entrez/query.fcgi?cmd=search&db=gene&term=C130090K23Rik) | RIKEN cDNA C130090K23 gene |
| 0.00167 | 0.26452 | 33.3029668 | 8.2299132 | 4.0 | [1418480_at](https://www.affymetrix.com/LinkServlet?probeset=1418480_at) | [Ppbp](http://www.ncbi.nlm.nih.gov/entrez/query.fcgi?cmd=search&db=gene&term=Ppbp) | pro-platelet basic protein |
| 0.009939 | 0.304874 | 339.3367828 | 85.1739952 | 4.0 | [1427660_x_at](https://www.affymetrix.com/LinkServlet?probeset=1427660_x_at) | [Igk-V1](http://www.ncbi.nlm.nih.gov/entrez/query.fcgi?cmd=search&db=gene&term=Igk-V1) | immunoglobulin kappa chain variable 1 (V1) |
| 0.011959 | 0.311583 | 168.3152162 | 42.431814 | 4.0 | [1434046_at](https://www.affymetrix.com/LinkServlet?probeset=1434046_at) | [AA467197](http://www.ncbi.nlm.nih.gov/entrez/query.fcgi?cmd=search&db=gene&term=AA467197) | expressed sequence AA467197 |
| 0.001919 | 0.26452 | 378.4852407 | 95.4412285 | 4.0 | [1422123_s_at](https://www.affymetrix.com/LinkServlet?probeset=1422123_s_at) | [Ceacam1](http://www.ncbi.nlm.nih.gov/entrez/query.fcgi?cmd=search&db=gene&term=Ceacam1) | carcinoembryonic antigen-related cell adhesion molecule 1 |
| 0.005102 | 0.280839 | 48.6555462 | 12.2990745 | 4.0 | [1418907_at](https://www.affymetrix.com/LinkServlet?probeset=1418907_at) | [F5](http://www.ncbi.nlm.nih.gov/entrez/query.fcgi?cmd=search&db=gene&term=F5) | coagulation factor V |
| 0.021543 | 0.327626 | 420.8108989 | 108.779847 | 3.9 | [1425789_s_at](https://www.affymetrix.com/LinkServlet?probeset=1425789_s_at) | [Anxa8](http://www.ncbi.nlm.nih.gov/entrez/query.fcgi?cmd=search&db=gene&term=Anxa8) | annexin A8 |
| 0.002394 | 0.26452 | 266.1664437 | 70.0688206 | 3.8 | [1450494_x_at](https://www.affymetrix.com/LinkServlet?probeset=1450494_x_at) | [Ceacam1](http://www.ncbi.nlm.nih.gov/entrez/query.fcgi?cmd=search&db=gene&term=Ceacam1) | carcinoembryonic antigen-related cell adhesion molecule 1 |
| 0.004775 | 0.279106 | 161.0008252 | 42.544807 | 3.8 | [1452426_x_at](https://www.affymetrix.com/LinkServlet?probeset=1452426_x_at) | [NA](http://www.ncbi.nlm.nih.gov/entrez/query.fcgi?cmd=search&db=gene&term=NA) | NA |
| 0.003467 | 0.276588 | 108.9079986 | 29.4509285 | 3.7 | [1451699_at](https://www.affymetrix.com/LinkServlet?probeset=1451699_at) | [EG668468](http://www.ncbi.nlm.nih.gov/entrez/query.fcgi?cmd=search&db=gene&term=EG668468) | predicted gene, EG668468 |
| 0.003947 | 0.276588 | 235.6984886 | 63.998752 | 3.7 | [1437258_at](https://www.affymetrix.com/LinkServlet?probeset=1437258_at) | [100042999](http://www.ncbi.nlm.nih.gov/entrez/query.fcgi?cmd=search&db=gene&term=100042999) | predicted gene, 100042999 |
| 0.016082 | 0.320151 | 48.1345355 | 13.0731954 | 3.7 | [1420569_at](https://www.affymetrix.com/LinkServlet?probeset=1420569_at) | [Chad](http://www.ncbi.nlm.nih.gov/entrez/query.fcgi?cmd=search&db=gene&term=Chad) | chondroadherin |
| 0.005896 | 0.286924 | 277.3723594 | 76.5880873 | 3.6 | [1427455_x_at](https://www.affymetrix.com/LinkServlet?probeset=1427455_x_at) | [Igk-V1](http://www.ncbi.nlm.nih.gov/entrez/query.fcgi?cmd=search&db=gene&term=Igk-V1) | immunoglobulin kappa chain variable 1 (V1) |
| 0.006211 | 0.287694 | 166.0884845 | 46.52562 | 3.6 | [1416596_at](https://www.affymetrix.com/LinkServlet?probeset=1416596_at) | [Slc44a4](http://www.ncbi.nlm.nih.gov/entrez/query.fcgi?cmd=search&db=gene&term=Slc44a4) | solute carrier family 44, member 4 |
| 0.005045 | 0.280839 | 30.7599409 | 8.8340835 | 3.5 | [1418203_at](https://www.affymetrix.com/LinkServlet?probeset=1418203_at) | [Pmaip1](http://www.ncbi.nlm.nih.gov/entrez/query.fcgi?cmd=search&db=gene&term=Pmaip1) | phorbol-12-myristate-13-acetate-induced protein 1 |
| 0.002441 | 0.264903 | 128.63227 | 37.0103315 | 3.5 | [1427630_x_at](https://www.affymetrix.com/LinkServlet?probeset=1427630_x_at) | [Ceacam1](http://www.ncbi.nlm.nih.gov/entrez/query.fcgi?cmd=search&db=gene&term=Ceacam1) | carcinoembryonic antigen-related cell adhesion molecule 1 |
| 0.009142 | 0.302035 | 153.0124441 | 44.2034803 | 3.5 | [1417957_a_at](https://www.affymetrix.com/LinkServlet?probeset=1417957_a_at) | [Tspan1](http://www.ncbi.nlm.nih.gov/entrez/query.fcgi?cmd=search&db=gene&term=Tspan1) | tetraspanin 1 |
| 0.034686 | 0.349318 | 85.6002949 | 24.8716491 | 3.4 | [1449896_at](https://www.affymetrix.com/LinkServlet?probeset=1449896_at) | [Mlph](http://www.ncbi.nlm.nih.gov/entrez/query.fcgi?cmd=search&db=gene&term=Mlph) | melanophilin |
| 0.019005 | 0.324899 | 227.2611103 | 66.3592954 | 3.4 | [1428572_at](https://www.affymetrix.com/LinkServlet?probeset=1428572_at) | [Basp1](http://www.ncbi.nlm.nih.gov/entrez/query.fcgi?cmd=search&db=gene&term=Basp1) | brain abundant, membrane attached signal protein 1 |
| 0.00033 | 0.257465 | 1358.106422 | 405.0140875 | 3.4 | [1453238_s_at](https://www.affymetrix.com/LinkServlet?probeset=1453238_s_at) | [1200016E24Rik](http://www.ncbi.nlm.nih.gov/entrez/query.fcgi?cmd=search&db=gene&term=1200016E24Rik) | RIKEN cDNA 1200016E24 gene |
| 0.015579 | 0.319801 | 519.7290832 | 155.1831619 | 3.3 | [1415698_at](https://www.affymetrix.com/LinkServlet?probeset=1415698_at) | [Golm1](http://www.ncbi.nlm.nih.gov/entrez/query.fcgi?cmd=search&db=gene&term=Golm1) | golgi membrane protein 1 |
| 0.006648 | 0.289426 | 80.659459 | 24.2029838 | 3.3 | [1451498_at](https://www.affymetrix.com/LinkServlet?probeset=1451498_at) | [Lrrc26](http://www.ncbi.nlm.nih.gov/entrez/query.fcgi?cmd=search&db=gene&term=Lrrc26) | leucine rich repeat containing 26 |
| 0.006822 | 0.290571 | 21.3714812 | 6.4824727 | 3.3 | [1448290_at](https://www.affymetrix.com/LinkServlet?probeset=1448290_at) | [Reg3b](http://www.ncbi.nlm.nih.gov/entrez/query.fcgi?cmd=search&db=gene&term=Reg3b) | regenerating islet-derived 3 beta |
| 0.005432 | 0.281037 | 108.4493062 | 33.2145285 | 3.3 | [1455531_at](https://www.affymetrix.com/LinkServlet?probeset=1455531_at) | [Mfsd4](http://www.ncbi.nlm.nih.gov/entrez/query.fcgi?cmd=search&db=gene&term=Mfsd4) | major facilitator superfamily domain containing 4 |
| 0.000947 | 0.26452 | 112.0619395 | 34.4129034 | 3.3 | [1425854_x_at](https://www.affymetrix.com/LinkServlet?probeset=1425854_x_at) | [Tcrb-J](http://www.ncbi.nlm.nih.gov/entrez/query.fcgi?cmd=search&db=gene&term=Tcrb-J) | T-cell receptor beta, joining region |
| 0.015355 | 0.318692 | 67.631741 | 21.0078636 | 3.2 | [1455452_x_at](https://www.affymetrix.com/LinkServlet?probeset=1455452_x_at) | [AI449310](http://www.ncbi.nlm.nih.gov/entrez/query.fcgi?cmd=search&db=gene&term=AI449310) | expressed sequence AI449310 |
| 0.015962 | 0.320151 | 60.0535842 | 18.7076754 | 3.2 | [1424305_at](https://www.affymetrix.com/LinkServlet?probeset=1424305_at) | [Igj](http://www.ncbi.nlm.nih.gov/entrez/query.fcgi?cmd=search&db=gene&term=Igj) | immunoglobulin joining chain |
| 0.006436 | 0.287694 | 63.9114084 | 20.0936253 | 3.2 | [1448789_at](https://www.affymetrix.com/LinkServlet?probeset=1448789_at) | [Aldh1a3](http://www.ncbi.nlm.nih.gov/entrez/query.fcgi?cmd=search&db=gene&term=Aldh1a3) | aldehyde dehydrogenase family 1, subfamily A3 |
| 0.001542 | 0.26452 | 92.9474455 | 29.4042651 | 3.2 | [1420378_at](https://www.affymetrix.com/LinkServlet?probeset=1420378_at) | [Sftpd](http://www.ncbi.nlm.nih.gov/entrez/query.fcgi?cmd=search&db=gene&term=Sftpd) | surfactant associated protein D |
| 0.004719 | 0.278162 | 55.6761715 | 18.190147 | 3.1 | [1444088_at](https://www.affymetrix.com/LinkServlet?probeset=1444088_at) | [NA](http://www.ncbi.nlm.nih.gov/entrez/query.fcgi?cmd=search&db=gene&term=NA) | NA |
| 0.00423 | 0.277913 | 370.0105854 | 121.408599 | 3.0 | [1460682_s_at](https://www.affymetrix.com/LinkServlet?probeset=1460682_s_at) | [Ceacam2](http://www.ncbi.nlm.nih.gov/entrez/query.fcgi?cmd=search&db=gene&term=Ceacam2) | carcinoembryonic antigen-related cell adhesion molecule 2 |
| 0.014551 | 0.316828 | 20.7984753 | 6.8701493 | 3.0 | [1430234_at](https://www.affymetrix.com/LinkServlet?probeset=1430234_at) | [Arl14](http://www.ncbi.nlm.nih.gov/entrez/query.fcgi?cmd=search&db=gene&term=Arl14) | ADP-ribosylation factor-like 14 |
| 0.014755 | 0.316828 | 96.9511391 | 32.338184 | 3.0 | [1418405_at](https://www.affymetrix.com/LinkServlet?probeset=1418405_at) | [Hgfac](http://www.ncbi.nlm.nih.gov/entrez/query.fcgi?cmd=search&db=gene&term=Hgfac) | hepatocyte growth factor activator |
| 0.009678 | 0.304035 | 1390.231725 | 470.3419164 | 3.0 | [1429334_at](https://www.affymetrix.com/LinkServlet?probeset=1429334_at) | [Lyg1](http://www.ncbi.nlm.nih.gov/entrez/query.fcgi?cmd=search&db=gene&term=Lyg1) | lysozyme G-like 1 |
| 0.044224 | 0.364609 | 84.2668483 | 28.6837743 | 2.9 | [1449409_at](https://www.affymetrix.com/LinkServlet?probeset=1449409_at) | [Sult1c2](http://www.ncbi.nlm.nih.gov/entrez/query.fcgi?cmd=search&db=gene&term=Sult1c2) | sulfotransferase family, cytosolic, 1C, member 2 |
| 0.025711 | 0.339208 | 47.9708983 | 16.4451953 | 2.9 | [1447289_at](https://www.affymetrix.com/LinkServlet?probeset=1447289_at) | [AA763521](http://www.ncbi.nlm.nih.gov/entrez/query.fcgi?cmd=search&db=gene&term=AA763521) | expressed sequence AA763521 |
| 0.022826 | 0.329914 | 25.239961 | 8.6833769 | 2.9 | [1430963_at](https://www.affymetrix.com/LinkServlet?probeset=1430963_at) | [Gcnt3](http://www.ncbi.nlm.nih.gov/entrez/query.fcgi?cmd=search&db=gene&term=Gcnt3) | glucosaminyl (N-acetyl) transferase 3, mucin type |
| 0.003103 | 0.276542 | 78.488105 | 27.2837593 | 2.9 | [1419099_x_at](https://www.affymetrix.com/LinkServlet?probeset=1419099_x_at) | [Stom](http://www.ncbi.nlm.nih.gov/entrez/query.fcgi?cmd=search&db=gene&term=Stom) | stomatin |
| 0.020059 | 0.326003 | 44.4535807 | 15.5426909 | 2.9 | [1446748_at](https://www.affymetrix.com/LinkServlet?probeset=1446748_at) | [2010007H06Rik](http://www.ncbi.nlm.nih.gov/entrez/query.fcgi?cmd=search&db=gene&term=2010007H06Rik) | RIKEN cDNA 2010007H06 gene |
| 0.019134 | 0.324916 | 90.7307859 | 31.7905055 | 2.9 | [1421113_at](https://www.affymetrix.com/LinkServlet?probeset=1421113_at) | [Pga5](http://www.ncbi.nlm.nih.gov/entrez/query.fcgi?cmd=search&db=gene&term=Pga5) | pepsinogen 5, group I |
| 0.014944 | 0.316863 | 937.4029037 | 328.5508704 | 2.9 | [1423952_a_at](https://www.affymetrix.com/LinkServlet?probeset=1423952_a_at) | [Krt7](http://www.ncbi.nlm.nih.gov/entrez/query.fcgi?cmd=search&db=gene&term=Krt7) | keratin 7 |
| 0.018096 | 0.323469 | 581.4370961 | 204.0984552 | 2.8 | [1422672_at](https://www.affymetrix.com/LinkServlet?probeset=1422672_at) | [Sprr1b](http://www.ncbi.nlm.nih.gov/entrez/query.fcgi?cmd=search&db=gene&term=Sprr1b) | small proline-rich protein 1B |
| 0.024787 | 0.334589 | 68.9928386 | 24.4188824 | 2.8 | [1417745_at](https://www.affymetrix.com/LinkServlet?probeset=1417745_at) | [Cpn1](http://www.ncbi.nlm.nih.gov/entrez/query.fcgi?cmd=search&db=gene&term=Cpn1) | carboxypeptidase N, polypeptide 1 |
| 0.001113 | 0.26452 | 125.522878 | 44.6148331 | 2.8 | [1425226_x_at](https://www.affymetrix.com/LinkServlet?probeset=1425226_x_at) | [Tcrb-J](http://www.ncbi.nlm.nih.gov/entrez/query.fcgi?cmd=search&db=gene&term=Tcrb-J) | T-cell receptor beta, joining region |
| 0.016597 | 0.321101 | 104.3017567 | 37.1077131 | 2.8 | [1443337_at](https://www.affymetrix.com/LinkServlet?probeset=1443337_at) | [NA](http://www.ncbi.nlm.nih.gov/entrez/query.fcgi?cmd=search&db=gene&term=NA) | NA |
| 0.005337 | 0.281031 | 65.1586149 | 23.2153101 | 2.8 | [1452532_x_at](https://www.affymetrix.com/LinkServlet?probeset=1452532_x_at) | [Ceacam1](http://www.ncbi.nlm.nih.gov/entrez/query.fcgi?cmd=search&db=gene&term=Ceacam1) | carcinoembryonic antigen-related cell adhesion molecule 1 |
| 0.000948 | 0.26452 | 74.6366767 | 26.6041235 | 2.8 | [1419098_at](https://www.affymetrix.com/LinkServlet?probeset=1419098_at) | [Stom](http://www.ncbi.nlm.nih.gov/entrez/query.fcgi?cmd=search&db=gene&term=Stom) | stomatin |
| 0.011445 | 0.3094 | 71.3224783 | 25.4889559 | 2.8 | [1451139_at](https://www.affymetrix.com/LinkServlet?probeset=1451139_at) | [Slc39a4](http://www.ncbi.nlm.nih.gov/entrez/query.fcgi?cmd=search&db=gene&term=Slc39a4) | solute carrier family 39 (zinc transporter), member 4 |
| 0.031137 | 0.343746 | 727.3803024 | 260.3542964 | 2.8 | [1427878_at](https://www.affymetrix.com/LinkServlet?probeset=1427878_at) | [0610010O12Rik](http://www.ncbi.nlm.nih.gov/entrez/query.fcgi?cmd=search&db=gene&term=0610010O12Rik) | RIKEN cDNA 0610010O12 gene |
| 0.004596 | 0.278162 | 139.4063295 | 50.0378579 | 2.8 | [1419476_at](https://www.affymetrix.com/LinkServlet?probeset=1419476_at) | [Adamdec1](http://www.ncbi.nlm.nih.gov/entrez/query.fcgi?cmd=search&db=gene&term=Adamdec1) | ADAM-like, decysin 1 |
| 0.022826 | 0.329914 | 34.2177589 | 12.3619041 | 2.8 | [1452227_at](https://www.affymetrix.com/LinkServlet?probeset=1452227_at) | [2310045A20Rik](http://www.ncbi.nlm.nih.gov/entrez/query.fcgi?cmd=search&db=gene&term=2310045A20Rik) | RIKEN cDNA 2310045A20 gene |
| 0.000719 | 0.26452 | 72.6495557 | 26.4855141 | 2.7 | [1417408_at](https://www.affymetrix.com/LinkServlet?probeset=1417408_at) | [F3](http://www.ncbi.nlm.nih.gov/entrez/query.fcgi?cmd=search&db=gene&term=F3) | coagulation factor III |
| 0.020782 | 0.327066 | 117.0218176 | 42.7389557 | 2.7 | [1429381_x_at](https://www.affymetrix.com/LinkServlet?probeset=1429381_x_at) | [Igh](http://www.ncbi.nlm.nih.gov/entrez/query.fcgi?cmd=search&db=gene&term=Igh) | immunoglobulin heavy chain complex |
| 0.020084 | 0.326003 | 33.1412365 | 12.1098323 | 2.7 | [1449269_at](https://www.affymetrix.com/LinkServlet?probeset=1449269_at) | [F5](http://www.ncbi.nlm.nih.gov/entrez/query.fcgi?cmd=search&db=gene&term=F5) | coagulation factor V |
| 0.001302 | 0.26452 | 204.4683771 | 74.8983831 | 2.7 | [1445561_at](https://www.affymetrix.com/LinkServlet?probeset=1445561_at) | [NA](http://www.ncbi.nlm.nih.gov/entrez/query.fcgi?cmd=search&db=gene&term=NA) | NA |
| 0.002252 | 0.26452 | 28.9138575 | 10.6223168 | 2.7 | [1422865_at](https://www.affymetrix.com/LinkServlet?probeset=1422865_at) | [Runx1](http://www.ncbi.nlm.nih.gov/entrez/query.fcgi?cmd=search&db=gene&term=Runx1) | runt related transcription factor 1 |
| 0.008631 | 0.302035 | 117.745329 | 43.400835 | 2.7 | [1420017_at](https://www.affymetrix.com/LinkServlet?probeset=1420017_at) | [Tspan8](http://www.ncbi.nlm.nih.gov/entrez/query.fcgi?cmd=search&db=gene&term=Tspan8) | tetraspanin 8 |
| 0.021424 | 0.327626 | 24.0439619 | 8.9190514 | 2.7 | [1439489_at](https://www.affymetrix.com/LinkServlet?probeset=1439489_at) | [Gpr120](http://www.ncbi.nlm.nih.gov/entrez/query.fcgi?cmd=search&db=gene&term=Gpr120) | G protein-coupled receptor 120 |
| 0.00922 | 0.302745 | 129.4530638 | 48.0351095 | 2.7 | [1425675_s_at](https://www.affymetrix.com/LinkServlet?probeset=1425675_s_at) | [Ceacam1](http://www.ncbi.nlm.nih.gov/entrez/query.fcgi?cmd=search&db=gene&term=Ceacam1) | carcinoembryonic antigen-related cell adhesion molecule 1 |
| 0.029025 | 0.341697 | 36.0136274 | 13.3814537 | 2.7 | [1448837_at](https://www.affymetrix.com/LinkServlet?probeset=1448837_at) | [Vil1](http://www.ncbi.nlm.nih.gov/entrez/query.fcgi?cmd=search&db=gene&term=Vil1) | villin 1 |
| 0.003133 | 0.276588 | 35.2937449 | 13.1981331 | 2.7 | [1427711_a_at](https://www.affymetrix.com/LinkServlet?probeset=1427711_a_at) | [Ceacam1](http://www.ncbi.nlm.nih.gov/entrez/query.fcgi?cmd=search&db=gene&term=Ceacam1) | carcinoembryonic antigen-related cell adhesion molecule 1 |
| 0.035427 | 0.35093 | 97.3734525 | 36.4704017 | 2.7 | [1442447_at](https://www.affymetrix.com/LinkServlet?probeset=1442447_at) | [NA](http://www.ncbi.nlm.nih.gov/entrez/query.fcgi?cmd=search&db=gene&term=NA) | NA |
| 0.044797 | 0.364984 | 21.860538 | 8.290146 | 2.7 | [1455451_at](https://www.affymetrix.com/LinkServlet?probeset=1455451_at) | [AI449310](http://www.ncbi.nlm.nih.gov/entrez/query.fcgi?cmd=search&db=gene&term=AI449310) | expressed sequence AI449310 |
| 0.005217 | 0.280839 | 191.1815018 | 72.7094664 | 2.6 | [1451610_at](https://www.affymetrix.com/LinkServlet?probeset=1451610_at) | [Cxcl17](http://www.ncbi.nlm.nih.gov/entrez/query.fcgi?cmd=search&db=gene&term=Cxcl17) | chemokine (C-X-C motif) ligand 17 |
| 0.043568 | 0.364468 | 135.3946713 | 51.586241 | 2.6 | [1427013_at](https://www.affymetrix.com/LinkServlet?probeset=1427013_at) | [Car9](http://www.ncbi.nlm.nih.gov/entrez/query.fcgi?cmd=search&db=gene&term=Car9) | carbonic anhydrase 9 |
| 0.004222 | 0.277913 | 35.0446597 | 13.5061681 | 2.6 | [1422864_at](https://www.affymetrix.com/LinkServlet?probeset=1422864_at) | [Runx1](http://www.ncbi.nlm.nih.gov/entrez/query.fcgi?cmd=search&db=gene&term=Runx1) | runt related transcription factor 1 |
| 0.01181 | 0.311244 | 99.9663574 | 38.6153884 | 2.6 | [1427102_at](https://www.affymetrix.com/LinkServlet?probeset=1427102_at) | [Slfn4](http://www.ncbi.nlm.nih.gov/entrez/query.fcgi?cmd=search&db=gene&term=Slfn4) | schlafen 4 |
| 0.016584 | 0.321101 | 15.3493629 | 5.9376304 | 2.6 | [1444980_at](https://www.affymetrix.com/LinkServlet?probeset=1444980_at) | [Onecut2](http://www.ncbi.nlm.nih.gov/entrez/query.fcgi?cmd=search&db=gene&term=Onecut2) | one cut domain, family member 2 |
| 0.022276 | 0.328881 | 144.0658476 | 56.259648 | 2.6 | [1423693_at](https://www.affymetrix.com/LinkServlet?probeset=1423693_at) | [Ela1](http://www.ncbi.nlm.nih.gov/entrez/query.fcgi?cmd=search&db=gene&term=Ela1) | elastase 1, pancreatic |
| 0.000583 | 0.26452 | 197.5036522 | 77.3561099 | 2.6 | [1417821_at](https://www.affymetrix.com/LinkServlet?probeset=1417821_at) | [D17H6S56E-5](http://www.ncbi.nlm.nih.gov/entrez/query.fcgi?cmd=search&db=gene&term=D17H6S56E-5) | DNA segment, Chr 17, human D6S56E 5 |
| 0.049188 | 0.373599 | 27.4852229 | 10.8279703 | 2.5 | [1425324_x_at](https://www.affymetrix.com/LinkServlet?probeset=1425324_x_at) | [Igh-6](http://www.ncbi.nlm.nih.gov/entrez/query.fcgi?cmd=search&db=gene&term=Igh-6) | immunoglobulin heavy chain 6 (heavy chain of IgM) |
| 0.007916 | 0.298818 | 114.4122857 | 45.2968468 | 2.5 | [1457040_at](https://www.affymetrix.com/LinkServlet?probeset=1457040_at) | [Lgi2](http://www.ncbi.nlm.nih.gov/entrez/query.fcgi?cmd=search&db=gene&term=Lgi2) | leucine-rich repeat LGI family, member 2 |
| 0.001606 | 0.26452 | 26.830549 | 10.6361851 | 2.5 | [1436100_at](https://www.affymetrix.com/LinkServlet?probeset=1436100_at) | [Kif17](http://www.ncbi.nlm.nih.gov/entrez/query.fcgi?cmd=search&db=gene&term=Kif17) | kinesin family member 17 |
| 0.004979 | 0.280674 | 21.0259305 | 8.3412709 | 2.5 | [1421775_at](https://www.affymetrix.com/LinkServlet?probeset=1421775_at) | [Fcer1a](http://www.ncbi.nlm.nih.gov/entrez/query.fcgi?cmd=search&db=gene&term=Fcer1a) | Fc receptor, IgE, high affinity I, alpha polypeptide |
| 0.017037 | 0.322189 | 530.9789059 | 210.6743173 | 2.5 | [1451336_at](https://www.affymetrix.com/LinkServlet?probeset=1451336_at) | [Lgals4](http://www.ncbi.nlm.nih.gov/entrez/query.fcgi?cmd=search&db=gene&term=Lgals4) | lectin, galactose binding, soluble 4 |
| 0.011249 | 0.307827 | 204.9007577 | 81.3924245 | 2.5 | [1449994_at](https://www.affymetrix.com/LinkServlet?probeset=1449994_at) | [Epgn](http://www.ncbi.nlm.nih.gov/entrez/query.fcgi?cmd=search&db=gene&term=Epgn) | epithelial mitogen |
| 0.02002 | 0.326003 | 23.0349887 | 9.1730509 | 2.5 | [1460468_s_at](https://www.affymetrix.com/LinkServlet?probeset=1460468_s_at) | [Dnajc22](http://www.ncbi.nlm.nih.gov/entrez/query.fcgi?cmd=search&db=gene&term=Dnajc22) | DnaJ (Hsp40) homolog, subfamily C, member 22 |
| 0.021396 | 0.327626 | 246.2328179 | 98.438709 | 2.5 | [1432558_a_at](https://www.affymetrix.com/LinkServlet?probeset=1432558_a_at) | [Mal](http://www.ncbi.nlm.nih.gov/entrez/query.fcgi?cmd=search&db=gene&term=Mal) | myelin and lymphocyte protein, T-cell differentiation protein |
| 0.002229 | 0.26452 | 21.6000058 | 8.6773047 | 2.5 | [1448872_at](https://www.affymetrix.com/LinkServlet?probeset=1448872_at) | [Reg3g](http://www.ncbi.nlm.nih.gov/entrez/query.fcgi?cmd=search&db=gene&term=Reg3g) | regenerating islet-derived 3 gamma |
| 0.03903 | 0.357763 | 50.9983034 | 20.52916 | 2.5 | [1429805_at](https://www.affymetrix.com/LinkServlet?probeset=1429805_at) | [Myo1a](http://www.ncbi.nlm.nih.gov/entrez/query.fcgi?cmd=search&db=gene&term=Myo1a) | myosin IA |
| 0.00047 | 0.26452 | 185.8109038 | 74.8371574 | 2.5 | [1426159_x_at](https://www.affymetrix.com/LinkServlet?probeset=1426159_x_at) | [Tcrb-J](http://www.ncbi.nlm.nih.gov/entrez/query.fcgi?cmd=search&db=gene&term=Tcrb-J) | T-cell receptor beta, joining region |
| 0.003241 | 0.276588 | 112.6051532 | 45.3835169 | 2.5 | [1426772_x_at](https://www.affymetrix.com/LinkServlet?probeset=1426772_x_at) | [Tcrb-J](http://www.ncbi.nlm.nih.gov/entrez/query.fcgi?cmd=search&db=gene&term=Tcrb-J) | T-cell receptor beta, joining region |
| 0.016753 | 0.321318 | 101.3976878 | 40.9909501 | 2.5 | [1427063_at](https://www.affymetrix.com/LinkServlet?probeset=1427063_at) | [5330417C22Rik](http://www.ncbi.nlm.nih.gov/entrez/query.fcgi?cmd=search&db=gene&term=5330417C22Rik) | RIKEN cDNA 5330417C22 gene |
| 0.006544 | 0.288348 | 359.9769822 | 145.8960001 | 2.5 | [1434399_at](https://www.affymetrix.com/LinkServlet?probeset=1434399_at) | [Galnt6](http://www.ncbi.nlm.nih.gov/entrez/query.fcgi?cmd=search&db=gene&term=Galnt6) | UDP-N-acetyl-alpha-D-galactosamine:polypeptide N-acetylgalactosaminyltransferase 6 |
| 0.022461 | 0.328881 | 582.8249125 | 236.5458168 | 2.5 | [1416191_at](https://www.affymetrix.com/LinkServlet?probeset=1416191_at) | [Sec61a1](http://www.ncbi.nlm.nih.gov/entrez/query.fcgi?cmd=search&db=gene&term=Sec61a1) | Sec61 alpha 1 subunit (S. cerevisiae) |
| 5.81E-05 | 0.166874 | 850.6167004 | 346.3382736 | 2.5 | [1415823_at](https://www.affymetrix.com/LinkServlet?probeset=1415823_at) | [Scd2](http://www.ncbi.nlm.nih.gov/entrez/query.fcgi?cmd=search&db=gene&term=Scd2) | stearoyl-Coenzyme A desaturase 2 |
| 0.002471 | 0.266279 | 5723.558882 | 2361.837058 | 2.4 | [1455892_x_at](https://www.affymetrix.com/LinkServlet?probeset=1455892_x_at) | [NA](http://www.ncbi.nlm.nih.gov/entrez/query.fcgi?cmd=search&db=gene&term=NA) | NA |
| 0.041854 | 0.361895 | 47.518206 | 19.6116191 | 2.4 | [1450455_s_at](https://www.affymetrix.com/LinkServlet?probeset=1450455_s_at) | [Akr1c12](http://www.ncbi.nlm.nih.gov/entrez/query.fcgi?cmd=search&db=gene&term=Akr1c12) | aldo-keto reductase family 1, member C12 |
| 0.032568 | 0.344316 | 99.1079002 | 41.1589124 | 2.4 | [1450747_at](https://www.affymetrix.com/LinkServlet?probeset=1450747_at) | [Keap1](http://www.ncbi.nlm.nih.gov/entrez/query.fcgi?cmd=search&db=gene&term=Keap1) | kelch-like ECH-associated protein 1 |
| 0.046083 | 0.367325 | 13.9091432 | 5.8068947 | 2.4 | [1427482_a_at](https://www.affymetrix.com/LinkServlet?probeset=1427482_a_at) | [Car8](http://www.ncbi.nlm.nih.gov/entrez/query.fcgi?cmd=search&db=gene&term=Car8) | carbonic anhydrase 8 |
| 0.010911 | 0.30565 | 88.3274664 | 37.0737383 | 2.4 | [1420503_at](https://www.affymetrix.com/LinkServlet?probeset=1420503_at) | [Slc6a14](http://www.ncbi.nlm.nih.gov/entrez/query.fcgi?cmd=search&db=gene&term=Slc6a14) | solute carrier family 6 (neurotransmitter transporter), member 14 |
| 0.042795 | 0.363753 | 26.3005997 | 11.0697972 | 2.4 | [1418304_at](https://www.affymetrix.com/LinkServlet?probeset=1418304_at) | [Pcdh21](http://www.ncbi.nlm.nih.gov/entrez/query.fcgi?cmd=search&db=gene&term=Pcdh21) | protocadherin 21 |
| 0.004803 | 0.279106 | 24.8860947 | 10.6547202 | 2.3 | [1434531_at](https://www.affymetrix.com/LinkServlet?probeset=1434531_at) | [Mgat5b](http://www.ncbi.nlm.nih.gov/entrez/query.fcgi?cmd=search&db=gene&term=Mgat5b) | mannoside acetylglucosaminyltransferase 5, isoenzyme B |
| 0.003407 | 0.276588 | 66.5580089 | 28.5706915 | 2.3 | [1417160_s_at](https://www.affymetrix.com/LinkServlet?probeset=1417160_s_at) | [Expi](http://www.ncbi.nlm.nih.gov/entrez/query.fcgi?cmd=search&db=gene&term=Expi) | extracellular proteinase inhibitor |
| 0.043863 | 0.364609 | 63.1877655 | 27.1295643 | 2.3 | [1425247_a_at](https://www.affymetrix.com/LinkServlet?probeset=1425247_a_at) | [Igh-6](http://www.ncbi.nlm.nih.gov/entrez/query.fcgi?cmd=search&db=gene&term=Igh-6) | immunoglobulin heavy chain 6 (heavy chain of IgM) |
| 0.004234 | 0.277913 | 81.3871654 | 35.1315028 | 2.3 | [1450220_a_at](https://www.affymetrix.com/LinkServlet?probeset=1450220_a_at) | [Spdef](http://www.ncbi.nlm.nih.gov/entrez/query.fcgi?cmd=search&db=gene&term=Spdef) | SAM pointed domain containing ets transcription factor |
| 0.031997 | 0.344316 | 44.5226883 | 19.2605126 | 2.3 | [1450139_at](https://www.affymetrix.com/LinkServlet?probeset=1450139_at) | [Ern2](http://www.ncbi.nlm.nih.gov/entrez/query.fcgi?cmd=search&db=gene&term=Ern2) | endoplasmic reticulum (ER) to nucleus signalling 2 |
| 0.017955 | 0.323469 | 73.9624099 | 32.2181313 | 2.3 | [1439661_at](https://www.affymetrix.com/LinkServlet?probeset=1439661_at) | [Slc16a14](http://www.ncbi.nlm.nih.gov/entrez/query.fcgi?cmd=search&db=gene&term=Slc16a14) | solute carrier family 16 (monocarboxylic acid transporters), member 14 |
| 0.023801 | 0.332147 | 62.9280614 | 27.461422 | 2.3 | [1425464_at](https://www.affymetrix.com/LinkServlet?probeset=1425464_at) | [Gata6](http://www.ncbi.nlm.nih.gov/entrez/query.fcgi?cmd=search&db=gene&term=Gata6) | GATA binding protein 6 |
| 0.011602 | 0.310763 | 54.7506699 | 23.966162 | 2.3 | [1451424_at](https://www.affymetrix.com/LinkServlet?probeset=1451424_at) | [Gabrp](http://www.ncbi.nlm.nih.gov/entrez/query.fcgi?cmd=search&db=gene&term=Gabrp) | gamma-aminobutyric acid (GABA-A) receptor, pi |
| 0.018936 | 0.324897 | 45.3599879 | 19.8663743 | 2.3 | [1425809_at](https://www.affymetrix.com/LinkServlet?probeset=1425809_at) | [Fabp4](http://www.ncbi.nlm.nih.gov/entrez/query.fcgi?cmd=search&db=gene&term=Fabp4) | fatty acid binding protein 4, adipocyte |
| 0.021036 | 0.327626 | 238.4337818 | 104.7659053 | 2.3 | [1426597_s_at](https://www.affymetrix.com/LinkServlet?probeset=1426597_s_at) | [C79267](http://www.ncbi.nlm.nih.gov/entrez/query.fcgi?cmd=search&db=gene&term=C79267) | expressed sequence C79267 |
| 0.017322 | 0.323469 | 131.7033131 | 58.1456006 | 2.3 | [1427870_x_at](https://www.affymetrix.com/LinkServlet?probeset=1427870_x_at) | [Igh-6](http://www.ncbi.nlm.nih.gov/entrez/query.fcgi?cmd=search&db=gene&term=Igh-6) | immunoglobulin heavy chain 6 (heavy chain of IgM) |
| 0.002148 | 0.26452 | 459.051628 | 202.7556154 | 2.3 | [1424351_at](https://www.affymetrix.com/LinkServlet?probeset=1424351_at) | [Wfdc2](http://www.ncbi.nlm.nih.gov/entrez/query.fcgi?cmd=search&db=gene&term=Wfdc2) | WAP four-disulfide core domain 2 |
| 0.015059 | 0.317372 | 93.351 | 41.3258759 | 2.3 | [1439506_at](https://www.affymetrix.com/LinkServlet?probeset=1439506_at) | [Gm98](http://www.ncbi.nlm.nih.gov/entrez/query.fcgi?cmd=search&db=gene&term=Gm98) | gene model 98, (NCBI) |
| 0.030174 | 0.342719 | 47.3765172 | 21.0789199 | 2.2 | [1417618_at](https://www.affymetrix.com/LinkServlet?probeset=1417618_at) | [Itih2](http://www.ncbi.nlm.nih.gov/entrez/query.fcgi?cmd=search&db=gene&term=Itih2) | inter-alpha trypsin inhibitor, heavy chain 2 |
| 0.014179 | 0.316828 | 21.9467048 | 9.7656053 | 2.2 | [1451597_at](https://www.affymetrix.com/LinkServlet?probeset=1451597_at) | [Tmprss11d](http://www.ncbi.nlm.nih.gov/entrez/query.fcgi?cmd=search&db=gene&term=Tmprss11d) | transmembrane protease, serine 11d |
| 0.028207 | 0.341697 | 152.0375365 | 67.7024598 | 2.2 | [1429496_x_at](https://www.affymetrix.com/LinkServlet?probeset=1429496_x_at) | [2300002M23Rik](http://www.ncbi.nlm.nih.gov/entrez/query.fcgi?cmd=search&db=gene&term=2300002M23Rik) | RIKEN cDNA 2300002M23 gene |
| 0.030969 | 0.343746 | 67.7288193 | 30.2385505 | 2.2 | [1448409_at](https://www.affymetrix.com/LinkServlet?probeset=1448409_at) | [Lrmp](http://www.ncbi.nlm.nih.gov/entrez/query.fcgi?cmd=search&db=gene&term=Lrmp) | lymphoid-restricted membrane protein |
| 0.007378 | 0.293537 | 67.8368712 | 30.410612 | 2.2 | [1424127_at](https://www.affymetrix.com/LinkServlet?probeset=1424127_at) | [Eya2](http://www.ncbi.nlm.nih.gov/entrez/query.fcgi?cmd=search&db=gene&term=Eya2) | eyes absent 2 homolog (Drosophila) |
| 0.002268 | 0.26452 | 508.3581686 | 228.1067918 | 2.2 | [1450616_at](https://www.affymetrix.com/LinkServlet?probeset=1450616_at) | [Ear5](http://www.ncbi.nlm.nih.gov/entrez/query.fcgi?cmd=search&db=gene&term=Ear5) | eosinophil-associated, ribonuclease A family, member 5 |
| 0.041331 | 0.360568 | 90.0188776 | 40.5769774 | 2.2 | [1451502_at](https://www.affymetrix.com/LinkServlet?probeset=1451502_at) | [Pla2g10](http://www.ncbi.nlm.nih.gov/entrez/query.fcgi?cmd=search&db=gene&term=Pla2g10) | phospholipase A2, group X |
| 0.002325 | 0.26452 | 75.064111 | 33.9411336 | 2.2 | [1427424_at](https://www.affymetrix.com/LinkServlet?probeset=1427424_at) | [Galnt6](http://www.ncbi.nlm.nih.gov/entrez/query.fcgi?cmd=search&db=gene&term=Galnt6) | UDP-N-acetyl-alpha-D-galactosamine:polypeptide N-acetylgalactosaminyltransferase 6 |
| 0.027156 | 0.341517 | 41.3032799 | 18.6881127 | 2.2 | [1426300_at](https://www.affymetrix.com/LinkServlet?probeset=1426300_at) | [Alcam](http://www.ncbi.nlm.nih.gov/entrez/query.fcgi?cmd=search&db=gene&term=Alcam) | activated leukocyte cell adhesion molecule |
| 0.002242 | 0.26452 | 15.5244987 | 7.0548058 | 2.2 | [1418937_at](https://www.affymetrix.com/LinkServlet?probeset=1418937_at) | [Dio2](http://www.ncbi.nlm.nih.gov/entrez/query.fcgi?cmd=search&db=gene&term=Dio2) | deiodinase, iodothyronine, type II |
| 0.003287 | 0.276588 | 2384.98499 | 1084.83977 | 2.2 | [1448756_at](https://www.affymetrix.com/LinkServlet?probeset=1448756_at) | [S100a9](http://www.ncbi.nlm.nih.gov/entrez/query.fcgi?cmd=search&db=gene&term=S100a9) | S100 calcium binding protein A9 (calgranulin B) |
| 0.01596 | 0.320151 | 232.4757275 | 105.9334252 | 2.2 | [1423933_a_at](https://www.affymetrix.com/LinkServlet?probeset=1423933_a_at) | [1600029D21Rik](http://www.ncbi.nlm.nih.gov/entrez/query.fcgi?cmd=search&db=gene&term=1600029D21Rik) | RIKEN cDNA 1600029D21 gene |
| 0.012307 | 0.312777 | 195.3403094 | 89.0702862 | 2.2 | [1440147_at](https://www.affymetrix.com/LinkServlet?probeset=1440147_at) | [Lgi2](http://www.ncbi.nlm.nih.gov/entrez/query.fcgi?cmd=search&db=gene&term=Lgi2) | leucine-rich repeat LGI family, member 2 |
| 0.016178 | 0.320151 | 79.3572025 | 36.3787699 | 2.2 | [1430479_at](https://www.affymetrix.com/LinkServlet?probeset=1430479_at) | [2010007H06Rik](http://www.ncbi.nlm.nih.gov/entrez/query.fcgi?cmd=search&db=gene&term=2010007H06Rik) | RIKEN cDNA 2010007H06 gene |
| 0.045894 | 0.366892 | 258.8542327 | 119.11113 | 2.2 | [1454883_at](https://www.affymetrix.com/LinkServlet?probeset=1454883_at) | [Gsdmc2](http://www.ncbi.nlm.nih.gov/entrez/query.fcgi?cmd=search&db=gene&term=Gsdmc2) | gasdermin C2 |
| 0.001109 | 0.26452 | 6400.435144 | 2951.131317 | 2.2 | [1420771_at](https://www.affymetrix.com/LinkServlet?probeset=1420771_at) | [Sprr2d](http://www.ncbi.nlm.nih.gov/entrez/query.fcgi?cmd=search&db=gene&term=Sprr2d) | small proline-rich protein 2D |
| 0.036247 | 0.353289 | 18.3456432 | 8.477504 | 2.2 | [1424901_at](https://www.affymetrix.com/LinkServlet?probeset=1424901_at) | [Gcnt3](http://www.ncbi.nlm.nih.gov/entrez/query.fcgi?cmd=search&db=gene&term=Gcnt3) | glucosaminyl (N-acetyl) transferase 3, mucin type |
| 0.007382 | 0.293537 | 34.7928127 | 16.1078371 | 2.2 | [1429183_at](https://www.affymetrix.com/LinkServlet?probeset=1429183_at) | [Pkp2](http://www.ncbi.nlm.nih.gov/entrez/query.fcgi?cmd=search&db=gene&term=Pkp2) | plakophilin 2 |
| 0.02192 | 0.32853 | 78.8174046 | 36.6625124 | 2.1 | [1417600_at](https://www.affymetrix.com/LinkServlet?probeset=1417600_at) | [Slc15a2](http://www.ncbi.nlm.nih.gov/entrez/query.fcgi?cmd=search&db=gene&term=Slc15a2) | solute carrier family 15 (H+/peptide transporter), member 2 |
| 0.018525 | 0.323481 | 231.660493 | 107.928263 | 2.1 | [1416190_a_at](https://www.affymetrix.com/LinkServlet?probeset=1416190_a_at) | [Sec61a1](http://www.ncbi.nlm.nih.gov/entrez/query.fcgi?cmd=search&db=gene&term=Sec61a1) | Sec61 alpha 1 subunit (S. cerevisiae) |
| 0.002341 | 0.26452 | 349.7174714 | 163.036965 | 2.1 | [1417822_at](https://www.affymetrix.com/LinkServlet?probeset=1417822_at) | [D17H6S56E-5](http://www.ncbi.nlm.nih.gov/entrez/query.fcgi?cmd=search&db=gene&term=D17H6S56E-5) | DNA segment, Chr 17, human D6S56E 5 |
| 0.031233 | 0.343746 | 109.4892251 | 51.2757657 | 2.1 | [1448766_at](https://www.affymetrix.com/LinkServlet?probeset=1448766_at) | [Gjb1](http://www.ncbi.nlm.nih.gov/entrez/query.fcgi?cmd=search&db=gene&term=Gjb1) | gap junction protein, beta 1 |
| 0.011115 | 0.307384 | 91.5596742 | 42.9232514 | 2.1 | [1458930_at](https://www.affymetrix.com/LinkServlet?probeset=1458930_at) | [A4gnt](http://www.ncbi.nlm.nih.gov/entrez/query.fcgi?cmd=search&db=gene&term=A4gnt) | alpha-1,4-N-acetylglucosaminyltransferase |
| 0.041801 | 0.361895 | 53.1578904 | 24.9426194 | 2.1 | [1443408_at](https://www.affymetrix.com/LinkServlet?probeset=1443408_at) | [NA](http://www.ncbi.nlm.nih.gov/entrez/query.fcgi?cmd=search&db=gene&term=NA) | NA |
| 0.014167 | 0.316828 | 102.4290736 | 48.1543507 | 2.1 | [1424339_at](https://www.affymetrix.com/LinkServlet?probeset=1424339_at) | [Oasl1](http://www.ncbi.nlm.nih.gov/entrez/query.fcgi?cmd=search&db=gene&term=Oasl1) | 2@#$%&-5@#$%& oligoadenylate synthetase-like 1 |
| 0.002581 | 0.267906 | 347.0786619 | 163.283022 | 2.1 | [1434905_at](https://www.affymetrix.com/LinkServlet?probeset=1434905_at) | [Ndufa4l2](http://www.ncbi.nlm.nih.gov/entrez/query.fcgi?cmd=search&db=gene&term=Ndufa4l2) | NADH dehydrogenase (ubiquinone) 1 alpha subcomplex, 4-like 2 |
| 0.002736 | 0.272369 | 24.0238863 | 11.3711807 | 2.1 | [1452463_x_at](https://www.affymetrix.com/LinkServlet?probeset=1452463_x_at) | [Igk-V1](http://www.ncbi.nlm.nih.gov/entrez/query.fcgi?cmd=search&db=gene&term=Igk-V1) | immunoglobulin kappa chain variable 1 (V1) |
| 0.031845 | 0.344316 | 18.9548847 | 9.01504 | 2.1 | [1420504_at](https://www.affymetrix.com/LinkServlet?probeset=1420504_at) | [Slc6a14](http://www.ncbi.nlm.nih.gov/entrez/query.fcgi?cmd=search&db=gene&term=Slc6a14) | solute carrier family 6 (neurotransmitter transporter), member 14 |
| 0.000274 | 0.257465 | 21.3408689 | 10.1988214 | 2.1 | [1419314_at](https://www.affymetrix.com/LinkServlet?probeset=1419314_at) | [Tinag](http://www.ncbi.nlm.nih.gov/entrez/query.fcgi?cmd=search&db=gene&term=Tinag) | tubulointerstitial nephritis antigen |
| 0.00627 | 0.287694 | 149.7345938 | 71.5636814 | 2.1 | [1454254_s_at](https://www.affymetrix.com/LinkServlet?probeset=1454254_s_at) | [1600029D21Rik](http://www.ncbi.nlm.nih.gov/entrez/query.fcgi?cmd=search&db=gene&term=1600029D21Rik) | RIKEN cDNA 1600029D21 gene |
| 0.029331 | 0.341697 | 417.3489788 | 199.9414542 | 2.1 | [1444061_at](https://www.affymetrix.com/LinkServlet?probeset=1444061_at) | [A030004J04Rik](http://www.ncbi.nlm.nih.gov/entrez/query.fcgi?cmd=search&db=gene&term=A030004J04Rik) | RIKEN cDNA A030004J04 gene |
| 0.023911 | 0.332147 | 54.0769245 | 25.9394689 | 2.1 | [1437466_at](https://www.affymetrix.com/LinkServlet?probeset=1437466_at) | [Alcam](http://www.ncbi.nlm.nih.gov/entrez/query.fcgi?cmd=search&db=gene&term=Alcam) | activated leukocyte cell adhesion molecule |
| 0.014993 | 0.317295 | 132.8344583 | 63.9984347 | 2.1 | [1421134_at](https://www.affymetrix.com/LinkServlet?probeset=1421134_at) | [Areg](http://www.ncbi.nlm.nih.gov/entrez/query.fcgi?cmd=search&db=gene&term=Areg) | amphiregulin |
| 0.027543 | 0.341648 | 63.7233661 | 30.7079554 | 2.1 | [1444262_at](https://www.affymetrix.com/LinkServlet?probeset=1444262_at) | [1110017F19Rik](http://www.ncbi.nlm.nih.gov/entrez/query.fcgi?cmd=search&db=gene&term=1110017F19Rik) | RIKEN cDNA 1110017F19 gene |
| 0.008419 | 0.300016 | 50.6019561 | 24.4320294 | 2.1 | [1451499_at](https://www.affymetrix.com/LinkServlet?probeset=1451499_at) | [Cadps2](http://www.ncbi.nlm.nih.gov/entrez/query.fcgi?cmd=search&db=gene&term=Cadps2) | Ca2+-dependent activator protein for secretion 2 |
| 0.02092 | 0.327564 | 81.5825857 | 39.4183292 | 2.1 | [1426633_s_at](https://www.affymetrix.com/LinkServlet?probeset=1426633_s_at) | [Kctd14](http://www.ncbi.nlm.nih.gov/entrez/query.fcgi?cmd=search&db=gene&term=Kctd14) | potassium channel tetramerisation domain containing 14 |
| 0.034005 | 0.347737 | 91.6821059 | 44.3509989 | 2.1 | [1419148_at](https://www.affymetrix.com/LinkServlet?probeset=1419148_at) | [Avil](http://www.ncbi.nlm.nih.gov/entrez/query.fcgi?cmd=search&db=gene&term=Avil) | advillin |
| 0.016233 | 0.320151 | 45.0085908 | 21.9006961 | 2.1 | [1422211_a_at](https://www.affymetrix.com/LinkServlet?probeset=1422211_a_at) | [B3gnt3](http://www.ncbi.nlm.nih.gov/entrez/query.fcgi?cmd=search&db=gene&term=B3gnt3) | UDP-GlcNAc:betaGal beta-1,3-N-acetylglucosaminyltransferase 3 |
| 0.028485 | 0.341697 | 231.9261257 | 112.9512818 | 2.1 | [1460248_at](https://www.affymetrix.com/LinkServlet?probeset=1460248_at) | [Cpxm2](http://www.ncbi.nlm.nih.gov/entrez/query.fcgi?cmd=search&db=gene&term=Cpxm2) | carboxypeptidase X 2 (M14 family) |
| 0.025643 | 0.338961 | 46.1514967 | 22.5073173 | 2.1 | [1446730_at](https://www.affymetrix.com/LinkServlet?probeset=1446730_at) | [NA](http://www.ncbi.nlm.nih.gov/entrez/query.fcgi?cmd=search&db=gene&term=NA) | NA |
| 0.02923 | 0.341697 | 56.1275475 | 27.3768528 | 2.1 | [1426663_s_at](https://www.affymetrix.com/LinkServlet?probeset=1426663_s_at) | [Slc45a3](http://www.ncbi.nlm.nih.gov/entrez/query.fcgi?cmd=search&db=gene&term=Slc45a3) | solute carrier family 45, member 3 |
| 0.00186 | 0.26452 | 174.5630661 | 85.3311906 | 2.0 | [1424775_at](https://www.affymetrix.com/LinkServlet?probeset=1424775_at) | [Oas1g](http://www.ncbi.nlm.nih.gov/entrez/query.fcgi?cmd=search&db=gene&term=Oas1g) | oligoadenylate synthetase 1G |
| 0.002761 | 0.272369 | 368.2255113 | 180.1193114 | 2.0 | [1422425_at](https://www.affymetrix.com/LinkServlet?probeset=1422425_at) | [Sprr2k](http://www.ncbi.nlm.nih.gov/entrez/query.fcgi?cmd=search&db=gene&term=Sprr2k) | small proline-rich protein 2K |
| 0.02084 | 0.32727 | 38.9082481 | 19.0951547 | 2.0 | [1455274_at](https://www.affymetrix.com/LinkServlet?probeset=1455274_at) | [NA](http://www.ncbi.nlm.nih.gov/entrez/query.fcgi?cmd=search&db=gene&term=NA) | NA |
| 0.014831 | 0.316828 | 80.8368524 | 39.7005807 | 2.0 | [1443339_at](https://www.affymetrix.com/LinkServlet?probeset=1443339_at) | [2310056P07Rik](http://www.ncbi.nlm.nih.gov/entrez/query.fcgi?cmd=search&db=gene&term=2310056P07Rik) | RIKEN cDNA 2310056P07 gene |
| 0.010519 | 0.305162 | 79.3736957 | 39.094602 | 2.0 | [1449341_a_at](https://www.affymetrix.com/LinkServlet?probeset=1449341_a_at) | [Stom](http://www.ncbi.nlm.nih.gov/entrez/query.fcgi?cmd=search&db=gene&term=Stom) | stomatin |
| 0.007236 | 0.293015 | 46.2749666 | 22.7950773 | 2.0 | [1427119_at](https://www.affymetrix.com/LinkServlet?probeset=1427119_at) | [Spink4](http://www.ncbi.nlm.nih.gov/entrez/query.fcgi?cmd=search&db=gene&term=Spink4) | serine peptidase inhibitor, Kazal type 4 |
| 0.029054 | 0.341697 | 211.6877649 | 104.8161179 | 2.0 | [1450383_at](https://www.affymetrix.com/LinkServlet?probeset=1450383_at) | [Ldlr](http://www.ncbi.nlm.nih.gov/entrez/query.fcgi?cmd=search&db=gene&term=Ldlr) | low density lipoprotein receptor |
| 0.008122 | 0.300016 | 150.6636079 | 74.6797732 | 2.0 | [1420062_at](https://www.affymetrix.com/LinkServlet?probeset=1420062_at) | [NA](http://www.ncbi.nlm.nih.gov/entrez/query.fcgi?cmd=search&db=gene&term=NA) | NA |
| 0.008964 | 0.302035 | 204.4134414 | 102.0287898 | 2.0 | [1424849_at](https://www.affymetrix.com/LinkServlet?probeset=1424849_at) | [Wdr62](http://www.ncbi.nlm.nih.gov/entrez/query.fcgi?cmd=search&db=gene&term=Wdr62) | WD repeat domain 62 |
| 0.014798 | 0.316828 | 460.9614832 | 230.2715753 | 2.0 | [1418350_at](https://www.affymetrix.com/LinkServlet?probeset=1418350_at) | [Hbegf](http://www.ncbi.nlm.nih.gov/entrez/query.fcgi?cmd=search&db=gene&term=Hbegf) | heparin-binding EGF-like growth factor |
| 0.002909 | 0.276227 | 64.3629933 | 32.4210795 | 2.0 | [1435951_at](https://www.affymetrix.com/LinkServlet?probeset=1435951_at) | [Grip1](http://www.ncbi.nlm.nih.gov/entrez/query.fcgi?cmd=search&db=gene&term=Grip1) | glutamate receptor interacting protein 1 |
| 0.002568 | 0.267906 | 2324.44964 | 1173.234909 | 2.0 | [1453503_at](https://www.affymetrix.com/LinkServlet?probeset=1453503_at) | [Spink12](http://www.ncbi.nlm.nih.gov/entrez/query.fcgi?cmd=search&db=gene&term=Spink12) | serine peptidase inhibitor, Kazal type 11 |
| 0.017879 | 0.323469 | 67.6681032 | 34.1765236 | 2.0 | [1453794_at](https://www.affymetrix.com/LinkServlet?probeset=1453794_at) | [Fer1l4](http://www.ncbi.nlm.nih.gov/entrez/query.fcgi?cmd=search&db=gene&term=Fer1l4) | fer-1-like 4 (C. elegans) |
| 0.006889 | 0.290571 | 72.255833 | 36.5077102 | 2.0 | [1449615_s_at](https://www.affymetrix.com/LinkServlet?probeset=1449615_s_at) | [Hdlbp](http://www.ncbi.nlm.nih.gov/entrez/query.fcgi?cmd=search&db=gene&term=Hdlbp) | high density lipoprotein (HDL) binding protein |
| 0.002878 | 0.276191 | 118.0199065 | 59.6608898 | 2.0 | [1428936_at](https://www.affymetrix.com/LinkServlet?probeset=1428936_at) | [Atp2b1](http://www.ncbi.nlm.nih.gov/entrez/query.fcgi?cmd=search&db=gene&term=Atp2b1) | ATPase, Ca++ transporting, plasma membrane 1 |
| 0.001044 | 0.26452 | 23.0419057 | 11.6613871 | 2.0 | [1417232_at](https://www.affymetrix.com/LinkServlet?probeset=1417232_at) | [Cldn2](http://www.ncbi.nlm.nih.gov/entrez/query.fcgi?cmd=search&db=gene&term=Cldn2) | claudin 2 |
| 0.024225 | 0.332823 | 16.8410158 | 8.5602326 | 2.0 | [1443086_at](https://www.affymetrix.com/LinkServlet?probeset=1443086_at) | [Alcam](http://www.ncbi.nlm.nih.gov/entrez/query.fcgi?cmd=search&db=gene&term=Alcam) | activated leukocyte cell adhesion molecule |
| 0.017026 | 0.322189 | 50.0981313 | 25.48852 | 2.0 | [1437467_at](https://www.affymetrix.com/LinkServlet?probeset=1437467_at) | [Alcam](http://www.ncbi.nlm.nih.gov/entrez/query.fcgi?cmd=search&db=gene&term=Alcam) | activated leukocyte cell adhesion molecule |
| 0.016603 | 0.321101 | 44.0250107 | 22.4030739 | 2.0 | [1452934_at](https://www.affymetrix.com/LinkServlet?probeset=1452934_at) | [Tmc5](http://www.ncbi.nlm.nih.gov/entrez/query.fcgi?cmd=search&db=gene&term=Tmc5) | transmembrane channel-like gene family 5 |
| 0.001006 | 0.26452 | 552.9154004 | 281.9374751 | 2.0 | [1441629_at](https://www.affymetrix.com/LinkServlet?probeset=1441629_at) | [NA](http://www.ncbi.nlm.nih.gov/entrez/query.fcgi?cmd=search&db=gene&term=NA) | NA |
| 0.042306 | 0.362951 | 175.4236871 | 89.5352761 | 2.0 | [1448541_at](https://www.affymetrix.com/LinkServlet?probeset=1448541_at) | [Klc1](http://www.ncbi.nlm.nih.gov/entrez/query.fcgi?cmd=search&db=gene&term=Klc1) | kinesin light chain 1 |
| 0.009924 | 0.304874 | 299.5640255 | 153.2470822 | 2.0 | [1448767_s_at](https://www.affymetrix.com/LinkServlet?probeset=1448767_s_at) | [Gjb1](http://www.ncbi.nlm.nih.gov/entrez/query.fcgi?cmd=search&db=gene&term=Gjb1) | gap junction protein, beta 1 |
| 0.002851 | 0.275799 | 82.4573271 | 525.1938923 | 0.16 | [1416612_at](https://www.affymetrix.com/LinkServlet?probeset=1416612_at) | [Cyp1b1](http://www.ncbi.nlm.nih.gov/entrez/query.fcgi?cmd=search&db=gene&term=Cyp1b1) | cytochrome P450, family 1, subfamily b, polypeptide 1 |
| 0.004911 | 0.279831 | 20.5675104 | 101.4764507 | 0.20 | [1416613_at](https://www.affymetrix.com/LinkServlet?probeset=1416613_at) | [Cyp1b1](http://www.ncbi.nlm.nih.gov/entrez/query.fcgi?cmd=search&db=gene&term=Cyp1b1) | cytochrome P450, family 1, subfamily b, polypeptide 1 |
| 0.044002 | 0.364609 | 4.6098021 | 15.7520464 | 0.29 | [1427868_x_at](https://www.affymetrix.com/LinkServlet?probeset=1427868_x_at) | [Myh1](http://www.ncbi.nlm.nih.gov/entrez/query.fcgi?cmd=search&db=gene&term=Myh1) | myosin, heavy polypeptide 1, skeletal muscle, adult |
| 0.00544 | 0.281037 | 45.3519228 | 154.0499954 | 0.29 | [1435621_at](https://www.affymetrix.com/LinkServlet?probeset=1435621_at) | [Far2](http://www.ncbi.nlm.nih.gov/entrez/query.fcgi?cmd=search&db=gene&term=Far2) | fatty acyl CoA reductase 2 |
| 0.003324 | 0.276588 | 19.4464313 | 57.6077031 | 0.34 | [1420796_at](https://www.affymetrix.com/LinkServlet?probeset=1420796_at) | [Ahrr](http://www.ncbi.nlm.nih.gov/entrez/query.fcgi?cmd=search&db=gene&term=Ahrr) | aryl-hydrocarbon receptor repressor |
| 0.013351 | 0.315296 | 174.2550957 | 504.1785157 | 0.34 | [1455889_at](https://www.affymetrix.com/LinkServlet?probeset=1455889_at) | [Far2](http://www.ncbi.nlm.nih.gov/entrez/query.fcgi?cmd=search&db=gene&term=Far2) | fatty acyl CoA reductase 2 |
| 0.001167 | 0.26452 | 201.1606119 | 575.3935722 | 0.35 | [1417751_at](https://www.affymetrix.com/LinkServlet?probeset=1417751_at) | [Stk10](http://www.ncbi.nlm.nih.gov/entrez/query.fcgi?cmd=search&db=gene&term=Stk10) | serine/threonine kinase 10 |
| 6.51E-05 | 0.166874 | 60.4471103 | 171.8250373 | 0.35 | [1419437_at](https://www.affymetrix.com/LinkServlet?probeset=1419437_at) | [Sim2](http://www.ncbi.nlm.nih.gov/entrez/query.fcgi?cmd=search&db=gene&term=Sim2) | single-minded homolog 2 (Drosophila) |
| 0.003401 | 0.276588 | 48.5121056 | 128.3698465 | 0.38 | [1440884_s_at](https://www.affymetrix.com/LinkServlet?probeset=1440884_s_at) | [A530047J11Rik](http://www.ncbi.nlm.nih.gov/entrez/query.fcgi?cmd=search&db=gene&term=A530047J11Rik) | RIKEN cDNA A530047J11 gene |
| 0.000712 | 0.26452 | 46.425213 | 121.1354961 | 0.38 | [1458680_at](https://www.affymetrix.com/LinkServlet?probeset=1458680_at) | [NA](http://www.ncbi.nlm.nih.gov/entrez/query.fcgi?cmd=search&db=gene&term=NA) | NA |
| 0.017691 | 0.323469 | 94.4147284 | 245.5619049 | 0.38 | [1431076_at](https://www.affymetrix.com/LinkServlet?probeset=1431076_at) | [Add2](http://www.ncbi.nlm.nih.gov/entrez/query.fcgi?cmd=search&db=gene&term=Add2) | adducin 2 (beta) |
| 2.35E-05 | 0.161134 | 17.3705628 | 45.0508218 | 0.39 | [1458345_s_at](https://www.affymetrix.com/LinkServlet?probeset=1458345_s_at) | [Colec11](http://www.ncbi.nlm.nih.gov/entrez/query.fcgi?cmd=search&db=gene&term=Colec11) | collectin sub-family member 11 |
| 0.031957 | 0.344316 | 31.8294091 | 81.6881055 | 0.39 | [1434264_at](https://www.affymetrix.com/LinkServlet?probeset=1434264_at) | [Ank2](http://www.ncbi.nlm.nih.gov/entrez/query.fcgi?cmd=search&db=gene&term=Ank2) | ankyrin 2, brain |
| 0.000896 | 0.26452 | 24.6001908 | 62.0759247 | 0. 40 | [1442721_at](https://www.affymetrix.com/LinkServlet?probeset=1442721_at) | [NA](http://www.ncbi.nlm.nih.gov/entrez/query.fcgi?cmd=search&db=gene&term=NA) | NA |
| 0.004638 | 0.278162 | 48.1587795 | 119.9007199 | 0.40 | [1439622_at](https://www.affymetrix.com/LinkServlet?probeset=1439622_at) | [Rassf4](http://www.ncbi.nlm.nih.gov/entrez/query.fcgi?cmd=search&db=gene&term=Rassf4) | Ras association (RalGDS/AF-6) domain family member 4 |
| 0.002042 | 0.26452 | 40.3515662 | 100.3893532 | 0.40 | [1456523_at](https://www.affymetrix.com/LinkServlet?probeset=1456523_at) | [100039239](http://www.ncbi.nlm.nih.gov/entrez/query.fcgi?cmd=search&db=gene&term=100039239) | predicted gene, 100039239 |
| 0.001556 | 0.26452 | 76.4397848 | 188.6823631 | 0.41 | [1436736_x_at](https://www.affymetrix.com/LinkServlet?probeset=1436736_x_at) | [D0H4S114](http://www.ncbi.nlm.nih.gov/entrez/query.fcgi?cmd=search&db=gene&term=D0H4S114) | DNA segment, human D4S114 |
| 0.005542 | 0.282385 | 52.0863535 | 127.7892976 | 0.41 | [1433551_at](https://www.affymetrix.com/LinkServlet?probeset=1433551_at) | [AI427515](http://www.ncbi.nlm.nih.gov/entrez/query.fcgi?cmd=search&db=gene&term=AI427515) | expressed sequence AI427515 |
| 0.001915 | 0.26452 | 52.6324398 | 129.0110003 | 0.41 | [1449009_at](https://www.affymetrix.com/LinkServlet?probeset=1449009_at) | [Tgtp](http://www.ncbi.nlm.nih.gov/entrez/query.fcgi?cmd=search&db=gene&term=Tgtp) | T-cell specific GTPase |
| 1.30E-06 | 0.029316 | 28.0385462 | 67.7102698 | 0.41 | [1419042_at](https://www.affymetrix.com/LinkServlet?probeset=1419042_at) | [Iigp1](http://www.ncbi.nlm.nih.gov/entrez/query.fcgi?cmd=search&db=gene&term=Iigp1) | interferon inducible GTPase 1 |
| 0.021081 | 0.327626 | 51.4513928 | 122.1201147 | 0.42 | [1449314_at](https://www.affymetrix.com/LinkServlet?probeset=1449314_at) | [Zfpm2](http://www.ncbi.nlm.nih.gov/entrez/query.fcgi?cmd=search&db=gene&term=Zfpm2) | zinc finger protein, multitype 2 |
| 0.000202 | 0.229386 | 178.0929312 | 412.2909076 | 0.43 | [1453279_x_at](https://www.affymetrix.com/LinkServlet?probeset=1453279_x_at) | [Krt76](http://www.ncbi.nlm.nih.gov/entrez/query.fcgi?cmd=search&db=gene&term=Krt76) | keratin 76 |
| 2.09E-05 | 0.161134 | 55.8178813 | 128.9966206 | 0.43 | [1437614_x_at](https://www.affymetrix.com/LinkServlet?probeset=1437614_x_at) | [Zdhhc14](http://www.ncbi.nlm.nih.gov/entrez/query.fcgi?cmd=search&db=gene&term=Zdhhc14) | zinc finger, DHHC domain containing 14 |
| 0.01858 | 0.323481 | 37.8346084 | 87.4059138 | 0.43 | [1427975_at](https://www.affymetrix.com/LinkServlet?probeset=1427975_at) | [Rasl10a](http://www.ncbi.nlm.nih.gov/entrez/query.fcgi?cmd=search&db=gene&term=Rasl10a) | RAS-like, family 10, member A |
| 0.013891 | 0.316828 | 65.2953888 | 149.6894162 | 0.44 | [1438531_at](https://www.affymetrix.com/LinkServlet?probeset=1438531_at) | [A730054J21Rik](http://www.ncbi.nlm.nih.gov/entrez/query.fcgi?cmd=search&db=gene&term=A730054J21Rik) | RIKEN cDNA A730054J21 gene |
| 0.031576 | 0.343746 | 202.2834795 | 458.5593928 | 0.44 | [1416713_at](https://www.affymetrix.com/LinkServlet?probeset=1416713_at) | [Tppp3](http://www.ncbi.nlm.nih.gov/entrez/query.fcgi?cmd=search&db=gene&term=Tppp3) | tubulin polymerization-promoting protein family member 3 |
| 0.02628 | 0.339666 | 25.4447243 | 57.5875856 | 0.44 | [1428114_at](https://www.affymetrix.com/LinkServlet?probeset=1428114_at) | [Slc14a1](http://www.ncbi.nlm.nih.gov/entrez/query.fcgi?cmd=search&db=gene&term=Slc14a1) | solute carrier family 14 (urea transporter), member 1 |
| 0.007444 | 0.293751 | 37.5631368 | 84.9107493 | 0.44 | [1426906_at](https://www.affymetrix.com/LinkServlet?probeset=1426906_at) | [Ifi205](http://www.ncbi.nlm.nih.gov/entrez/query.fcgi?cmd=search&db=gene&term=Ifi205) | interferon activated gene 205 |
| 0.00452 | 0.277913 | 17.9363646 | 40.5267123 | 0.44 | [1444965_at](https://www.affymetrix.com/LinkServlet?probeset=1444965_at) | [E130002L11Rik](http://www.ncbi.nlm.nih.gov/entrez/query.fcgi?cmd=search&db=gene&term=E130002L11Rik) | RIKEN cDNA E130002L11 gene |
| 5.43E-05 | 0.166874 | 95.8339266 | 211.8317003 | 0.45 | [1419043_a_at](https://www.affymetrix.com/LinkServlet?probeset=1419043_a_at) | [Iigp1](http://www.ncbi.nlm.nih.gov/entrez/query.fcgi?cmd=search&db=gene&term=Iigp1) | interferon inducible GTPase 1 |
| 0.03077 | 0.343746 | 70.4912066 | 153.9132567 | 0.46 | [1434265_s_at](https://www.affymetrix.com/LinkServlet?probeset=1434265_s_at) | [Ank2](http://www.ncbi.nlm.nih.gov/entrez/query.fcgi?cmd=search&db=gene&term=Ank2) | ankyrin 2, brain |
| 8.94E-05 | 0.207465 | 53.6266347 | 115.5881525 | 0.46 | [1419435_at](https://www.affymetrix.com/LinkServlet?probeset=1419435_at) | [Aox1](http://www.ncbi.nlm.nih.gov/entrez/query.fcgi?cmd=search&db=gene&term=Aox1) | aldehyde oxidase 1 |
| 0.001702 | 0.26452 | 50.3912736 | 108.5136303 | 0.46 | [1434909_at](https://www.affymetrix.com/LinkServlet?probeset=1434909_at) | [Rragd](http://www.ncbi.nlm.nih.gov/entrez/query.fcgi?cmd=search&db=gene&term=Rragd) | Ras-related GTP binding D |
| 6.07E-05 | 0.166874 | 131.7226094 | 282.4063314 | 0.47 | [1435906_x_at](https://www.affymetrix.com/LinkServlet?probeset=1435906_x_at) | [Gbp2](http://www.ncbi.nlm.nih.gov/entrez/query.fcgi?cmd=search&db=gene&term=Gbp2) | guanylate binding protein 2 |
| 0.00359 | 0.276588 | 90.0508527 | 192.1809748 | 0.47 | [1424797_a_at](https://www.affymetrix.com/LinkServlet?probeset=1424797_a_at) | [Pitx2](http://www.ncbi.nlm.nih.gov/entrez/query.fcgi?cmd=search&db=gene&term=Pitx2) | paired-like homeodomain transcription factor 2 |
| 0.009188 | 0.302035 | 253.0213083 | 539.6088276 | 0.47 | [1452107_s_at](https://www.affymetrix.com/LinkServlet?probeset=1452107_s_at) | [NA](http://www.ncbi.nlm.nih.gov/entrez/query.fcgi?cmd=search&db=gene&term=NA) | NA |
| 0.028711 | 0.341697 | 143.9914781 | 306.868222 | 0.47 | [1427038_at](https://www.affymetrix.com/LinkServlet?probeset=1427038_at) | [Penk1](http://www.ncbi.nlm.nih.gov/entrez/query.fcgi?cmd=search&db=gene&term=Penk1) | preproenkephalin 1 |
| 0.0029 | 0.276227 | 28.3895562 | 60.0943874 | 0.47 | [1456873_at](https://www.affymetrix.com/LinkServlet?probeset=1456873_at) | [Clic5](http://www.ncbi.nlm.nih.gov/entrez/query.fcgi?cmd=search&db=gene&term=Clic5) | chloride intracellular channel 5 |
| 0.009362 | 0.302941 | 34.3796871 | 72.6403357 | 0.47 | [1424737_at](https://www.affymetrix.com/LinkServlet?probeset=1424737_at) | [Thrsp](http://www.ncbi.nlm.nih.gov/entrez/query.fcgi?cmd=search&db=gene&term=Thrsp) | thyroid hormone responsive SPOT14 homolog (Rattus) |
| 0.016079 | 0.320151 | 92.350079 | 194.9947968 | 0.47 | [1418728_at](https://www.affymetrix.com/LinkServlet?probeset=1418728_at) | [Star](http://www.ncbi.nlm.nih.gov/entrez/query.fcgi?cmd=search&db=gene&term=Star) | steroidogenic acute regulatory protein |
| 0.015502 | 0.31881 | 91.121185 | 192.0778947 | 0.47 | [1455792_x_at](https://www.affymetrix.com/LinkServlet?probeset=1455792_x_at) | [Ndn](http://www.ncbi.nlm.nih.gov/entrez/query.fcgi?cmd=search&db=gene&term=Ndn) | necdin |
| 0.020175 | 0.326003 | 41.3479342 | 86.7284134 | 0.47 | [1452766_at](https://www.affymetrix.com/LinkServlet?probeset=1452766_at) | [Tppp](http://www.ncbi.nlm.nih.gov/entrez/query.fcgi?cmd=search&db=gene&term=Tppp) | tubulin polymerization promoting protein |
| 0.001523 | 0.26452 | 37.1526769 | 77.1841163 | 0.48 | [1422155_at](https://www.affymetrix.com/LinkServlet?probeset=1422155_at) | [Hist2h3c2](http://www.ncbi.nlm.nih.gov/entrez/query.fcgi?cmd=search&db=gene&term=Hist2h3c2) | histone cluster 2, H3c2 |
| 0.020929 | 0.327564 | 13.3797488 | 27.6965261 | 0.48 | [1452348_s_at](https://www.affymetrix.com/LinkServlet?probeset=1452348_s_at) | [Ifi205](http://www.ncbi.nlm.nih.gov/entrez/query.fcgi?cmd=search&db=gene&term=Ifi205) | interferon activated gene 205 |
| 0.002946 | 0.276289 | 1783.272629 | 3691.428923 | 0.48 | [1418752_at](https://www.affymetrix.com/LinkServlet?probeset=1418752_at) | [Aldh3a1](http://www.ncbi.nlm.nih.gov/entrez/query.fcgi?cmd=search&db=gene&term=Aldh3a1) | aldehyde dehydrogenase family 3, subfamily A1 |
| 0.002846 | 0.275799 | 134.5656118 | 278.0548135 | 0.48 | [1450839_at](https://www.affymetrix.com/LinkServlet?probeset=1450839_at) | [D0H4S114](http://www.ncbi.nlm.nih.gov/entrez/query.fcgi?cmd=search&db=gene&term=D0H4S114) | DNA segment, human D4S114 |
| 0.011588 | 0.310763 | 48.889866 | 100.8889554 | 0.48 | [1433933_s_at](https://www.affymetrix.com/LinkServlet?probeset=1433933_s_at) | [Slco2b1](http://www.ncbi.nlm.nih.gov/entrez/query.fcgi?cmd=search&db=gene&term=Slco2b1) | solute carrier organic anion transporter family, member 2b1 |
| 0.000291 | 0.257465 | 198.5122596 | 409.3850925 | 0.48 | [1457306_at](https://www.affymetrix.com/LinkServlet?probeset=1457306_at) | [NA](http://www.ncbi.nlm.nih.gov/entrez/query.fcgi?cmd=search&db=gene&term=NA) | NA |
| 0.007547 | 0.294522 | 252.0940488 | 519.7152551 | 0.49 | [1456156_at](https://www.affymetrix.com/LinkServlet?probeset=1456156_at) | [Lepr](http://www.ncbi.nlm.nih.gov/entrez/query.fcgi?cmd=search&db=gene&term=Lepr) | leptin receptor |
| 0.014102 | 0.316828 | 171.0210128 | 352.1585304 | 0.49 | [1424733_at](https://www.affymetrix.com/LinkServlet?probeset=1424733_at) | [P2ry14](http://www.ncbi.nlm.nih.gov/entrez/query.fcgi?cmd=search&db=gene&term=P2ry14) | purinergic receptor P2Y, G-protein coupled, 14 |
| 0.020168 | 0.326003 | 125.6110702 | 258.1716269 | 0.49 | [1425644_at](https://www.affymetrix.com/LinkServlet?probeset=1425644_at) | [Lepr](http://www.ncbi.nlm.nih.gov/entrez/query.fcgi?cmd=search&db=gene&term=Lepr) | leptin receptor |
| 0.007209 | 0.293015 | 174.7133864 | 358.518348 | 0.49 | [1429506_at](https://www.affymetrix.com/LinkServlet?probeset=1429506_at) | [Nkd1](http://www.ncbi.nlm.nih.gov/entrez/query.fcgi?cmd=search&db=gene&term=Nkd1) | naked cuticle 1 homolog (Drosophila) |
| 0.005791 | 0.286767 | 32.3002999 | 66.1633346 | 0.49 | [1435387_at](https://www.affymetrix.com/LinkServlet?probeset=1435387_at) | [Slc2a13](http://www.ncbi.nlm.nih.gov/entrez/query.fcgi?cmd=search&db=gene&term=Slc2a13) | solute carrier family 2 (facilitated glucose transporter), member 13 |
| 0.002163 | 0.26452 | 88.9045571 | 181.6261316 | 0.49 | [1427086_at](https://www.affymetrix.com/LinkServlet?probeset=1427086_at) | [Slit3](http://www.ncbi.nlm.nih.gov/entrez/query.fcgi?cmd=search&db=gene&term=Slit3) | slit homolog 3 (Drosophila) |
| 0.009333 | 0.302941 | 213.6318285 | 434.2743979 | 0.49 | [1453152_at](https://www.affymetrix.com/LinkServlet?probeset=1453152_at) | [Mamdc2](http://www.ncbi.nlm.nih.gov/entrez/query.fcgi?cmd=search&db=gene&term=Mamdc2) | MAM domain containing 2 |
| 0.008585 | 0.301797 | 1425.95725 | 2880.835688 | 0.49 | [1455077_a_at](https://www.affymetrix.com/LinkServlet?probeset=1455077_a_at) | [Morf4l1](http://www.ncbi.nlm.nih.gov/entrez/query.fcgi?cmd=search&db=gene&term=Morf4l1) | mortality factor 4 like 1 |
| 0.00497 | 0.280512 | 117.7169814 | 237.1968396 | 0.50 | [1455554_at](https://www.affymetrix.com/LinkServlet?probeset=1455554_at) | [A830039N20Rik](http://www.ncbi.nlm.nih.gov/entrez/query.fcgi?cmd=search&db=gene&term=A830039N20Rik) | RIKEN cDNA A830039N20 gene |
| 0.001277 | 0.26452 | 116.1247084 | 232.7094501 | 0.50 | [1456602_at](https://www.affymetrix.com/LinkServlet?probeset=1456602_at) | [4932417I16Rik](http://www.ncbi.nlm.nih.gov/entrez/query.fcgi?cmd=search&db=gene&term=4932417I16Rik) | RIKEN cDNA 4932417I16 gene |
| 0.000971 | 0.26452 | 86.5203667 | 173.1771643 | 0.50 | [1445332_at](https://www.affymetrix.com/LinkServlet?probeset=1445332_at) | [NA](http://www.ncbi.nlm.nih.gov/entrez/query.fcgi?cmd=search&db=gene&term=NA) | NA |
| 0.039733 | 0.359285 | 107.4369113 | 215.0298386 | 0.50 | [1435383_x_at](https://www.affymetrix.com/LinkServlet?probeset=1435383_x_at) | [Ndn](http://www.ncbi.nlm.nih.gov/entrez/query.fcgi?cmd=search&db=gene&term=Ndn) | necdin |
| 0.036677 | 0.353918 | 129.1961288 | 258.2918214 | 0.50 | [1435382_at](https://www.affymetrix.com/LinkServlet?probeset=1435382_at) | [Ndn](http://www.ncbi.nlm.nih.gov/entrez/query.fcgi?cmd=search&db=gene&term=Ndn) | necdin |
| 0.000218 | 0.229386 | 75.4470166 | 150.7893731 | 0.50 | [1438619_x_at](https://www.affymetrix.com/LinkServlet?probeset=1438619_x_at) | [Zdhhc14](http://www.ncbi.nlm.nih.gov/entrez/query.fcgi?cmd=search&db=gene&term=Zdhhc14) | zinc finger, DHHC domain containing 14 |
| 0.021631 | 0.327657 | 25.1454339 | 50.2000065 | 0.50 | [1460118_at](https://www.affymetrix.com/LinkServlet?probeset=1460118_at) | [NA](http://www.ncbi.nlm.nih.gov/entrez/query.fcgi?cmd=search&db=gene&term=NA) | NA |
| 0.00415 | 0.277913 | 55.6152161 | 110.9838193 | 0.50 | [1445963_at](https://www.affymetrix.com/LinkServlet?probeset=1445963_at) | [NA](http://www.ncbi.nlm.nih.gov/entrez/query.fcgi?cmd=search&db=gene&term=NA) | NA |
| 0.024527 | 0.334052 | 105.9028371 | 211.1904147 | 0.50 | [1428948_at](https://www.affymetrix.com/LinkServlet?probeset=1428948_at) | [5730414M22Rik](http://www.ncbi.nlm.nih.gov/entrez/query.fcgi?cmd=search&db=gene&term=5730414M22Rik) | RIKEN cDNA 5730414M22 gene |
| 0.042696 | 0.363753 | 40.0573709 | 79.8679969 | 0.50 | [1435343_at](https://www.affymetrix.com/LinkServlet?probeset=1435343_at) | [Dock10](http://www.ncbi.nlm.nih.gov/entrez/query.fcgi?cmd=search&db=gene&term=Dock10) | dedicator of cytokinesis 10 |
| 0.000172 | 0.229386 | 62.731829 | 124.9246875 | 0.50 | [1438975_x_at](https://www.affymetrix.com/LinkServlet?probeset=1438975_x_at) | [Zdhhc14](http://www.ncbi.nlm.nih.gov/entrez/query.fcgi?cmd=search&db=gene&term=Zdhhc14) | zinc finger, DHHC domain containing 14 |
| 0.010426 | 0.305162 | 37.2208699 | 74.0574433 | 0.50 | [1446127_at](https://www.affymetrix.com/LinkServlet?probeset=1446127_at) | [Zeb1](http://www.ncbi.nlm.nih.gov/entrez/query.fcgi?cmd=search&db=gene&term=Zeb1) | zinc finger E-box binding homeobox 1 |
| 0.004139 | 0.277913 | 210.5026754 | 417.8446553 | 0.50 | [1447830_s_at](https://www.affymetrix.com/LinkServlet?probeset=1447830_s_at) | [Rgs2](http://www.ncbi.nlm.nih.gov/entrez/query.fcgi?cmd=search&db=gene&term=Rgs2) | regulator of G-protein signaling 2 |
| 0.011639 | 0.310763 | 464.0818159 | 918.7981157 | 0.50 | [1425993_a_at](https://www.affymetrix.com/LinkServlet?probeset=1425993_a_at) | [Hsph1](http://www.ncbi.nlm.nih.gov/entrez/query.fcgi?cmd=search&db=gene&term=Hsph1) | heat shock 105kDa/110kDa protein 1 |
| 0.000173 | 0.229386 | 78.7804735 | 155.672979 | 0.50 | [1418392_a_at](https://www.affymetrix.com/LinkServlet?probeset=1418392_a_at) | [Gbp3](http://www.ncbi.nlm.nih.gov/entrez/query.fcgi?cmd=search&db=gene&term=Gbp3) | guanylate binding protein 3 |
| 0.039036 | 0.357763 | 57.8856521 | 114.3669352 | 0.50 | [1431248_at](https://www.affymetrix.com/LinkServlet?probeset=1431248_at) | [5031426D15Rik](http://www.ncbi.nlm.nih.gov/entrez/query.fcgi?cmd=search&db=gene&term=5031426D15Rik) | RIKEN cDNA 5031426D15 gene |
