# Supplementary material for: Zinc deficiency activates S100A8 inflammation in the absence of COX-2 and promotes murine oral-esophageal tumor progression
Source: Int J Cancer. 2010 Sep 20;129(2):331–45. doi: 10.1002/ijc.25688 (PMC3015018; doi:10.1002/ijc.25688)
Supplement: Supplementary file 4 [file ijc0129-0331-SD4.doc]

**Supporting Information Table 3.** Gene expression profile analysis of ZD:WT *vs* ZS:WT mouse forestomach

**Description of the problem:**

Number of classes: 2

Number of genes used for random variance estimation: 45101

Number of genes that passed filtering criteria: 7259

Type of univariate test used: Two-sample T-test (with random variance model)

Column of the Experiment Descriptors sheet that defines class variable: **ZD++ vs ZS++ (ZD:WT versus ZS:WT)**

Univariate test random variance model parameters: a= 1.38833, b= 18.96678 , Kolmogorov-Smirnov statistic= 0.01601

ZD = zinc-deficient; ZS = zinc-sufficient

Nominal significance level of each univariate test: 0.05

**Summary of Results:**

**Number of genes significant at 0.05 level of the univariate test: 463**

**Number of genes significant at 0.05 level and with a cut-off point of 2-fold or more difference: 67**

**Genes which discriminate among classes:**

Table - Sorted by p-value of the univariate test and a cut-off point of 2-fold or more difference

Class 1: *ZD++*; Class 2: *ZS++*.

**Up-regulated genes are in purple, down-regulated genes are in blue (43 up-regulated and 24 down-regulated)**

| **p-value** | **FDR** | **ZD++** | **ZS++** | **Fold-change** | **Probe set** | **Gene symbol** | **Description** |
| --- | --- | --- | --- | --- | --- | --- | --- |
| 0.00303 | 0.700639 | 218.238 | 20.91962 | 10 | [1423719_at](https://www.affymetrix.com/LinkServlet?probeset=1423719_at) | [U46068](http://www.ncbi.nlm.nih.gov/entrez/query.fcgi?cmd=search&db=gene&term=U46068) | cDNA sequence U46068 |
| 0.041188 | 0.700639 | 184.5726 | 20.23087 | 9.1 | [1449833_at](https://www.affymetrix.com/LinkServlet?probeset=1449833_at) | [Sprr2f](http://www.ncbi.nlm.nih.gov/entrez/query.fcgi?cmd=search&db=gene&term=Sprr2f) | small proline-rich protein 2F |
| 0.036991 | 0.700639 | 221.1309 | 26.2661 | 8.4 | [1422240_s_at](https://www.affymetrix.com/LinkServlet?probeset=1422240_s_at) | [Sprr2h](http://www.ncbi.nlm.nih.gov/entrez/query.fcgi?cmd=search&db=gene&term=Sprr2h) | small proline-rich protein 2H |
| 0.006543 | 0.700639 | 389.3189 | 53.07377 | 7.3 | [1422448_at](https://www.affymetrix.com/LinkServlet?probeset=1422448_at) | [Tff2](http://www.ncbi.nlm.nih.gov/entrez/query.fcgi?cmd=search&db=gene&term=Tff2) | trefoil factor 2 (spasmolytic protein 1) |
| 0.024162 | 0.700639 | 627.5183 | 87.93106 | 7.1 | [1422783_a_at](https://www.affymetrix.com/LinkServlet?probeset=1422783_a_at) | [Krt6a](http://www.ncbi.nlm.nih.gov/entrez/query.fcgi?cmd=search&db=gene&term=Krt6a) | keratin 6A |
| 0.00629 | 0.700639 | 111.9929 | 16.73546 | 6.7 | [1450060_at](https://www.affymetrix.com/LinkServlet?probeset=1450060_at) | [Pigr](http://www.ncbi.nlm.nih.gov/entrez/query.fcgi?cmd=search&db=gene&term=Pigr) | polymeric immunoglobulin receptor |
| 0.02932 | 0.700639 | 258.1109 | 41.8535 | 6.2 | [1421806_at](https://www.affymetrix.com/LinkServlet?probeset=1421806_at) | [Defb3](http://www.ncbi.nlm.nih.gov/entrez/query.fcgi?cmd=search&db=gene&term=Defb3) | defensin beta 3 |
| 0.01344 | 0.700639 | 149.8404 | 24.92137 | 6.0 | [1449426_a_at](https://www.affymetrix.com/LinkServlet?probeset=1449426_a_at) | [Anxa10](http://www.ncbi.nlm.nih.gov/entrez/query.fcgi?cmd=search&db=gene&term=Anxa10) | annexin A10 |
| 0.005548 | 0.700639 | 244.1862 | 42.96361 | 5.7 | [1439423_x_at](https://www.affymetrix.com/LinkServlet?probeset=1439423_x_at) | [U46068](http://www.ncbi.nlm.nih.gov/entrez/query.fcgi?cmd=search&db=gene&term=U46068) | cDNA sequence U46068 |
| 0.007134 | 0.700639 | 126.0035 | 23.99238 | 5.3 | [1455490_at](https://www.affymetrix.com/LinkServlet?probeset=1455490_at) | [Pigr](http://www.ncbi.nlm.nih.gov/entrez/query.fcgi?cmd=search&db=gene&term=Pigr) | polymeric immunoglobulin receptor |
| 0.016732 | 0.700639 | 102.5663 | 22.10541 | 4.6 | [1419268_at](https://www.affymetrix.com/LinkServlet?probeset=1419268_at) | [Agr2](http://www.ncbi.nlm.nih.gov/entrez/query.fcgi?cmd=search&db=gene&term=Agr2) | anterior gradient 2 (Xenopus laevis) |
| 0.017233 | 0.700639 | 93.4319 | 20.562 | 4.5 | [1418287_a_at](https://www.affymetrix.com/LinkServlet?probeset=1418287_a_at) | [Dmbt1](http://www.ncbi.nlm.nih.gov/entrez/query.fcgi?cmd=search&db=gene&term=Dmbt1) | deleted in malignant brain tumors 1 |
| 0.009765 | 0.700639 | 97.76915 | 21.6649 | 4.5 | [1416325_at](https://www.affymetrix.com/LinkServlet?probeset=1416325_at) | [Crisp1](http://www.ncbi.nlm.nih.gov/entrez/query.fcgi?cmd=search&db=gene&term=Crisp1) | cysteine-rich secretory protein 1 |
| 0.008077 | 0.700639 | 184.8608 | 43.93195 | 4.2 | [1419394_s_at](https://www.affymetrix.com/LinkServlet?probeset=1419394_s_at) | [S100a8](http://www.ncbi.nlm.nih.gov/entrez/query.fcgi?cmd=search&db=gene&term=S100a8) | S100 calcium binding protein A8 (calgranulin A) |
| 0.014718 | 0.700639 | 296.0545 | 76.94034 | 3.8 | [1418989_at](https://www.affymetrix.com/LinkServlet?probeset=1418989_at) | [Ctse](http://www.ncbi.nlm.nih.gov/entrez/query.fcgi?cmd=search&db=gene&term=Ctse) | cathepsin E |
| 0.023571 | 0.700639 | 276.3356 | 72.38084 | 3.8 | [1427700_x_at](https://www.affymetrix.com/LinkServlet?probeset=1427700_x_at) | [Krt6a](http://www.ncbi.nlm.nih.gov/entrez/query.fcgi?cmd=search&db=gene&term=Krt6a) | keratin 6A |
| 0.025935 | 0.700639 | 153.7837 | 42.11548 | 3.7 | [1417156_at](https://www.affymetrix.com/LinkServlet?probeset=1417156_at) | [Krt19](http://www.ncbi.nlm.nih.gov/entrez/query.fcgi?cmd=search&db=gene&term=Krt19) | keratin 19 |
| 0.005994 | 0.700639 | 84.37923 | 24.07776 | 3.5 | [1418724_at](https://www.affymetrix.com/LinkServlet?probeset=1418724_at) | [Cfi](http://www.ncbi.nlm.nih.gov/entrez/query.fcgi?cmd=search&db=gene&term=Cfi) | complement component factor i |
| 0.033951 | 0.700639 | 135.118 | 42.98963 | 3.1 | [1442447_at](https://www.affymetrix.com/LinkServlet?probeset=1442447_at) | [NA](http://www.ncbi.nlm.nih.gov/entrez/query.fcgi?cmd=search&db=gene&term=NA) | NA |
| 0.03955 | 0.700639 | 167.1261 | 56.47904 | 3.0 | [1423691_x_at](https://www.affymetrix.com/LinkServlet?probeset=1423691_x_at) | [Krt8](http://www.ncbi.nlm.nih.gov/entrez/query.fcgi?cmd=search&db=gene&term=Krt8) | keratin 8 |
| 0.012064 | 0.700639 | 292.9239 | 103.99 | 2.8 | [1444061_at](https://www.affymetrix.com/LinkServlet?probeset=1444061_at) | [A030004J04Rik](http://www.ncbi.nlm.nih.gov/entrez/query.fcgi?cmd=search&db=gene&term=A030004J04Rik) | RIKEN cDNA A030004J04 gene |
| 0.014263 | 0.700639 | 74.29131 | 28.03342 | 2.7 | [1443339_at](https://www.affymetrix.com/LinkServlet?probeset=1443339_at) | [2310056P07Rik](http://www.ncbi.nlm.nih.gov/entrez/query.fcgi?cmd=search&db=gene&term=2310056P07Rik) | RIKEN cDNA 2310056P07 gene |
| 0.02825 | 0.700639 | 45.66178 | 18.07786 | 2.5 | [1425522_at](https://www.affymetrix.com/LinkServlet?probeset=1425522_at) | [Rbm25](http://www.ncbi.nlm.nih.gov/entrez/query.fcgi?cmd=search&db=gene&term=Rbm25) | RNA binding motif protein 25 |
| 0.039684 | 0.700639 | 1708.174 | 699.1859 | 2.4 | [1448756_at](https://www.affymetrix.com/LinkServlet?probeset=1448756_at) | [S100a9](http://www.ncbi.nlm.nih.gov/entrez/query.fcgi?cmd=search&db=gene&term=S100a9) | S100 calcium binding protein A9 (calgranulin B) |
| 0.00794 | 0.700639 | 24.67921 | 10.28686 | 2.4 | [1454866_s_at](https://www.affymetrix.com/LinkServlet?probeset=1454866_s_at) | [Clic6](http://www.ncbi.nlm.nih.gov/entrez/query.fcgi?cmd=search&db=gene&term=Clic6) | chloride intracellular channel 6 |
| 0.02338 | 0.700639 | 125.8552 | 53.40195 | 2.4 | [1449199_at](https://www.affymetrix.com/LinkServlet?probeset=1449199_at) | [Muc1](http://www.ncbi.nlm.nih.gov/entrez/query.fcgi?cmd=search&db=gene&term=Muc1) | mucin 1, transmembrane |
| 0.005404 | 0.700639 | 118.2006 | 50.21602 | 2.3 | [1443026_at](https://www.affymetrix.com/LinkServlet?probeset=1443026_at) | [NA](http://www.ncbi.nlm.nih.gov/entrez/query.fcgi?cmd=search&db=gene&term=NA) | NA |
| 0.036252 | 0.700639 | 76.52136 | 33.25391 | 2.3 | [1454254_s_at](https://www.affymetrix.com/LinkServlet?probeset=1454254_s_at) | [1600029D21Rik](http://www.ncbi.nlm.nih.gov/entrez/query.fcgi?cmd=search&db=gene&term=1600029D21Rik) | RIKEN cDNA 1600029D21 gene |
| 0.006585 | 0.700639 | 120.8848 | 53.91129 | 2.2 | [1420062_at](https://www.affymetrix.com/LinkServlet?probeset=1420062_at) | [NA](http://www.ncbi.nlm.nih.gov/entrez/query.fcgi?cmd=search&db=gene&term=NA) | NA |
| 0.025904 | 0.700639 | 21.04441 | 9.433337 | 2.2 | [1418480_at](https://www.affymetrix.com/LinkServlet?probeset=1418480_at) | [Ppbp](http://www.ncbi.nlm.nih.gov/entrez/query.fcgi?cmd=search&db=gene&term=Ppbp) | pro-platelet basic protein |
| 0.017746 | 0.700639 | 124.9494 | 57.13353 | 2.2 | [1440916_at](https://www.affymetrix.com/LinkServlet?probeset=1440916_at) | [2510049J12Rik](http://www.ncbi.nlm.nih.gov/entrez/query.fcgi?cmd=search&db=gene&term=2510049J12Rik) | RIKEN cDNA 2510049J12 gene |
| 0.02748 | 0.700639 | 244.4598 | 114.996 | 2.1 | [1435460_at](https://www.affymetrix.com/LinkServlet?probeset=1435460_at) | [Prkg2](http://www.ncbi.nlm.nih.gov/entrez/query.fcgi?cmd=search&db=gene&term=Prkg2) | protein kinase, cGMP-dependent, type II |
| 0.045069 | 0.700639 | 71.51942 | 33.71735 | 2.1 | [1427353_at](https://www.affymetrix.com/LinkServlet?probeset=1427353_at) | [Clasp1](http://www.ncbi.nlm.nih.gov/entrez/query.fcgi?cmd=search&db=gene&term=Clasp1) | CLIP associating protein 1 |
| 0.032757 | 0.700639 | 469.1779 | 222.6804 | 2.1 | [1450616_at](https://www.affymetrix.com/LinkServlet?probeset=1450616_at) | [Ear5](http://www.ncbi.nlm.nih.gov/entrez/query.fcgi?cmd=search&db=gene&term=Ear5) | eosinophil-associated, ribonuclease A family, member 5 |
| 0.045717 | 0.700639 | 54.13382 | 26.03656 | 2.1 | [1455531_at](https://www.affymetrix.com/LinkServlet?probeset=1455531_at) | [Mfsd4](http://www.ncbi.nlm.nih.gov/entrez/query.fcgi?cmd=search&db=gene&term=Mfsd4) | major facilitator superfamily domain containing 4 |
| 0.004555 | 0.700639 | 21.78284 | 10.48441 | 2.1 | [1418907_at](https://www.affymetrix.com/LinkServlet?probeset=1418907_at) | [F5](http://www.ncbi.nlm.nih.gov/entrez/query.fcgi?cmd=search&db=gene&term=F5) | coagulation factor V |
| 0.016291 | 0.700639 | 42.30237 | 21.06227 | 2.0 | [1451424_at](https://www.affymetrix.com/LinkServlet?probeset=1451424_at) | [Gabrp](http://www.ncbi.nlm.nih.gov/entrez/query.fcgi?cmd=search&db=gene&term=Gabrp) | gamma-aminobutyric acid (GABA-A) receptor, pi |
| 0.025415 | 0.700639 | 18.10944 | 9.029292 | 2.0 | [1449254_at](https://www.affymetrix.com/LinkServlet?probeset=1449254_at) | [Spp1](http://www.ncbi.nlm.nih.gov/entrez/query.fcgi?cmd=search&db=gene&term=Spp1) | secreted phosphoprotein 1 |
| 0.02226 | 0.700639 | 591.9915 | 298.9633 | 2.0 | [1415823_at](https://www.affymetrix.com/LinkServlet?probeset=1415823_at) | [Scd2](http://www.ncbi.nlm.nih.gov/entrez/query.fcgi?cmd=search&db=gene&term=Scd2) | stearoyl-Coenzyme A desaturase 2 |
| 0.018714 | 0.700639 | 323.8903 | 163.9829 | 2.0 | [1417136_s_at](https://www.affymetrix.com/LinkServlet?probeset=1417136_s_at) | [Srpk2](http://www.ncbi.nlm.nih.gov/entrez/query.fcgi?cmd=search&db=gene&term=Srpk2) | serine/arginine-rich protein specific kinase 2 |
| 0.009307 | 0.700639 | 71.2778 | 36.27811 | 2.0 | [1445226_at](https://www.affymetrix.com/LinkServlet?probeset=1445226_at) | [BC023969](http://www.ncbi.nlm.nih.gov/entrez/query.fcgi?cmd=search&db=gene&term=BC023969) | cDNA sequence BC023969 |
| 0.035261 | 0.700639 | 190.6 | 97.55193 | 2.0 | [1422123_s_at](https://www.affymetrix.com/LinkServlet?probeset=1422123_s_at) | [Ceacam1](http://www.ncbi.nlm.nih.gov/entrez/query.fcgi?cmd=search&db=gene&term=Ceacam1) | carcinoembryonic antigen-related cell adhesion molecule 1 |
| 0.003048 | 0.700639 | 17.58893 | 9.011635 | 2.0 | [1449269_at](https://www.affymetrix.com/LinkServlet?probeset=1449269_at) | [F5](http://www.ncbi.nlm.nih.gov/entrez/query.fcgi?cmd=search&db=gene&term=F5) | coagulation factor V |
| 0.002268 | 0.700639 | 166.978 | 488.9893 | 0.34 | [1427038_at](https://www.affymetrix.com/LinkServlet?probeset=1427038_at) | [Penk1](http://www.ncbi.nlm.nih.gov/entrez/query.fcgi?cmd=search&db=gene&term=Penk1) | preproenkephalin 1 |
| 0.021844 | 0.700639 | 79.47554 | 229.3022 | 0.35 | [1431248_at](https://www.affymetrix.com/LinkServlet?probeset=1431248_at) | [5031426D15Rik](http://www.ncbi.nlm.nih.gov/entrez/query.fcgi?cmd=search&db=gene&term=5031426D15Rik) | RIKEN cDNA 5031426D15 gene |
| 0.009086 | 0.700639 | 28.86559 | 74.9706 | 0.39 | [1418826_at](https://www.affymetrix.com/LinkServlet?probeset=1418826_at) | [Ms4a6b](http://www.ncbi.nlm.nih.gov/entrez/query.fcgi?cmd=search&db=gene&term=Ms4a6b) | membrane-spanning 4-domains, subfamily A, member 6B |
| 0.008847 | 0.700639 | 49.39478 | 128.1444 | 0.39 | [1452349_x_at](https://www.affymetrix.com/LinkServlet?probeset=1452349_x_at) | [Ifi205](http://www.ncbi.nlm.nih.gov/entrez/query.fcgi?cmd=search&db=gene&term=Ifi205) | interferon activated gene 205 |
| 0.038144 | 0.700639 | 39.05064 | 98.56608 | 0.40 | [1422789_at](https://www.affymetrix.com/LinkServlet?probeset=1422789_at) | [Aldh1a2](http://www.ncbi.nlm.nih.gov/entrez/query.fcgi?cmd=search&db=gene&term=Aldh1a2) | aldehyde dehydrogenase family 1, subfamily A2 |
| 0.028583 | 0.700639 | 62.24286 | 156.1566 | 0.40 | [1440879_at](https://www.affymetrix.com/LinkServlet?probeset=1440879_at) | [Abca9](http://www.ncbi.nlm.nih.gov/entrez/query.fcgi?cmd=search&db=gene&term=Abca9) | ATP-binding cassette, sub-family A (ABC1), member 9 |
| 0.000683 | 0.700639 | 99.47266 | 244.992 | 0.41 | [1427086_at](https://www.affymetrix.com/LinkServlet?probeset=1427086_at) | [Slit3](http://www.ncbi.nlm.nih.gov/entrez/query.fcgi?cmd=search&db=gene&term=Slit3) | slit homolog 3 (Drosophila) |
| 0.01222 | 0.700639 | 16.7431 | 40.57765 | 0.41 | [1456295_at](https://www.affymetrix.com/LinkServlet?probeset=1456295_at) | [B230114P17Rik](http://www.ncbi.nlm.nih.gov/entrez/query.fcgi?cmd=search&db=gene&term=B230114P17Rik) | RIKEN cDNA B230114P17 gene |
| 0.018877 | 0.700639 | 73.51457 | 175.3282 | 0.42 | [1419070_at](https://www.affymetrix.com/LinkServlet?probeset=1419070_at) | [Cys1](http://www.ncbi.nlm.nih.gov/entrez/query.fcgi?cmd=search&db=gene&term=Cys1) | cystin 1 |
| 0.004917 | 0.700639 | 121.509 | 278.0201 | 0.44 | [1417235_at](https://www.affymetrix.com/LinkServlet?probeset=1417235_at) | [Ehd3](http://www.ncbi.nlm.nih.gov/entrez/query.fcgi?cmd=search&db=gene&term=Ehd3) | EH-domain containing 3 |
| 0.002729 | 0.700639 | 84.65437 | 190.0167 | 0.45 | [1452296_at](https://www.affymetrix.com/LinkServlet?probeset=1452296_at) | [Slit3](http://www.ncbi.nlm.nih.gov/entrez/query.fcgi?cmd=search&db=gene&term=Slit3) | slit homolog 3 (Drosophila) |
| 0.014434 | 0.700639 | 1416.511 | 3173.739 | 0.45 | [1437056_x_at](https://www.affymetrix.com/LinkServlet?probeset=1437056_x_at) | [Crispld2](http://www.ncbi.nlm.nih.gov/entrez/query.fcgi?cmd=search&db=gene&term=Crispld2) | cysteine-rich secretory protein LCCL domain containing 2 |
| 0.024507 | 0.700639 | 119.9089 | 266.4545 | 0.45 | [1436698_x_at](https://www.affymetrix.com/LinkServlet?probeset=1436698_x_at) | [Tmem204](http://www.ncbi.nlm.nih.gov/entrez/query.fcgi?cmd=search&db=gene&term=Tmem204) | transmembrane protein 204 |
| 0.00678 | 0.700639 | 44.69827 | 98.64412 | 0.45 | [1424737_at](https://www.affymetrix.com/LinkServlet?probeset=1424737_at) | [Thrsp](http://www.ncbi.nlm.nih.gov/entrez/query.fcgi?cmd=search&db=gene&term=Thrsp) | thyroid hormone responsive SPOT14 homolog (Rattus) |
| 0.029533 | 0.700639 | 74.72845 | 161.3596 | 0.46 | [1415904_at](https://www.affymetrix.com/LinkServlet?probeset=1415904_at) | [Lpl](http://www.ncbi.nlm.nih.gov/entrez/query.fcgi?cmd=search&db=gene&term=Lpl) | lipoprotein lipase |
| 0.000178 | 0.700639 | 392.6513 | 846.6492 | 0.46 | [1435459_at](https://www.affymetrix.com/LinkServlet?probeset=1435459_at) | [Fmo2](http://www.ncbi.nlm.nih.gov/entrez/query.fcgi?cmd=search&db=gene&term=Fmo2) | flavin containing monooxygenase 2 |
| 0.032797 | 0.700639 | 4.730499 | 9.837077 | 0.48 | [1427868_x_at](https://www.affymetrix.com/LinkServlet?probeset=1427868_x_at) | [Myh1](http://www.ncbi.nlm.nih.gov/entrez/query.fcgi?cmd=search&db=gene&term=Myh1) | myosin, heavy polypeptide 1, skeletal muscle, adult |
| 0.028852 | 0.700639 | 516.5624 | 1055.883 | 0.49 | [1435370_a_at](https://www.affymetrix.com/LinkServlet?probeset=1435370_a_at) | [Ces3](http://www.ncbi.nlm.nih.gov/entrez/query.fcgi?cmd=search&db=gene&term=Ces3) | carboxylesterase 3 |
| 0.028402 | 0.700639 | 117.7243 | 240.5598 | 0.49 | [1435879_at](https://www.affymetrix.com/LinkServlet?probeset=1435879_at) | [Akt3](http://www.ncbi.nlm.nih.gov/entrez/query.fcgi?cmd=search&db=gene&term=Akt3) | thymoma viral proto-oncogene 3 |
| 0.034002 | 0.700639 | 119.3659 | 242.5793 | 0.49 | [1418084_at](https://www.affymetrix.com/LinkServlet?probeset=1418084_at) | [Nrp1](http://www.ncbi.nlm.nih.gov/entrez/query.fcgi?cmd=search&db=gene&term=Nrp1) | neuropilin 1 |
| 0.001408 | 0.700639 | 1359.607 | 2757.742 | 0.49 | [1456341_a_at](https://www.affymetrix.com/LinkServlet?probeset=1456341_a_at) | [Klf9](http://www.ncbi.nlm.nih.gov/entrez/query.fcgi?cmd=search&db=gene&term=Klf9) | Kruppel-like factor 9 |
| 0.039815 | 0.700639 | 34.80858 | 69.53263 | 0.50 | [1439066_at](https://www.affymetrix.com/LinkServlet?probeset=1439066_at) | [Angpt1](http://www.ncbi.nlm.nih.gov/entrez/query.fcgi?cmd=search&db=gene&term=Angpt1) | angiopoietin 1 |
| 0.039252 | 0.700639 | 268.9471 | 534.6524 | 0.50 | [1448823_at](https://www.affymetrix.com/LinkServlet?probeset=1448823_at) | [Cxcl12](http://www.ncbi.nlm.nih.gov/entrez/query.fcgi?cmd=search&db=gene&term=Cxcl12) | chemokine (C-X-C motif) ligand 12 |
| 0.012043 | 0.700639 | 98.91886 | 195.8887 | 0.50 | [1438619_x_at](https://www.affymetrix.com/LinkServlet?probeset=1438619_x_at) | [Zdhhc14](http://www.ncbi.nlm.nih.gov/entrez/query.fcgi?cmd=search&db=gene&term=Zdhhc14) | zinc finger, DHHC domain containing 14 |
