# Supplementary material for: Zinc deficiency activates S100A8 inflammation in the absence of COX-2 and promotes murine oral-esophageal tumor progression
Source: Int J Cancer. 2010 Sep 20;129(2):331–45. doi: 10.1002/ijc.25688 (PMC3015018; doi:10.1002/ijc.25688)
Supplement: Supplementary file 5 [file ijc0129-0331-SD5.doc]

**Supporting Information Table 4.** Gene expression profile analysis of ZD:*Cox-2^-/-^* *vs* ZD:WT mouse forestomach

**Description of the problem:**

Number of classes: 2

Number of genes used for random variance estimation: 45101

Number of genes that passed filtering criteria: 7259

Type of univariate test used: Two-sample T-test (with random variance model)

Column of the Experiment Descriptors sheet that defines class variable : **ZD-- vs ZD++ (ZD:*Cox-2^-/-^* versus ZD:WT)**

Univariate test random variance model parameters: a= 1.89745 , b= 19.83534 , Kolmogorov-Smirnov statistic= 0.02292

ZD = zinc-deficient; ZS = zinc-sufficient

Nominal significance level of each univariate test: 0.05

**Summary of Results:**

**Number of genes significant at 0.05 level of the univariate test: 1860**

**Number of genes significant at 0.05 level and with a cut-off point of 2-fold or more difference: 90**

**Genes which discriminate among classes:**

Table - Sorted by p-value of the univariate test and a cut-off point of 2-fold or more difference

Class 1: *ZD--*; Class 2: *ZD++*

**Up-regulated genes are in purple, down-regulated genes are in blue (68 up-regulated and 22 down-regulated)**

| **p-value** | **FDR** | **ZD--** | **ZD++** | **Fold-change** | **Probe set** | **Gene symbol** | **Description** |
| --- | --- | --- | --- | --- | --- | --- | --- |
| 0.02315 | 0.1678758 | 2810.089 | 308.2985 | 9.1 | [1448932_at](https://www.affymetrix.com/LinkServlet?probeset=1448932_at) | [Krt16](http://www.ncbi.nlm.nih.gov/entrez/query.fcgi?cmd=search&db=gene&term=Krt16) | keratin 16 |
| 0.00582 | 0.1595038 | 814.0363 | 93.4319 | 8.7 | [1418287_a_at](https://www.affymetrix.com/LinkServlet?probeset=1418287_a_at) | [Dmbt1](http://www.ncbi.nlm.nih.gov/entrez/query.fcgi?cmd=search&db=gene&term=Dmbt1) | deleted in malignant brain tumors 1 |
| 0.02054 | 0.1638291 | 280.7756 | 34.19186 | 8.2 | [1449989_at](https://www.affymetrix.com/LinkServlet?probeset=1449989_at) | [Mcpt2](http://www.ncbi.nlm.nih.gov/entrez/query.fcgi?cmd=search&db=gene&term=Mcpt2) | mast cell protease 2 |
| 0.00222 | 0.1595038 | 1377.648 | 184.8608 | 7.5 | [1419394_s_at](https://www.affymetrix.com/LinkServlet?probeset=1419394_s_at) | [S100a8](http://www.ncbi.nlm.nih.gov/entrez/query.fcgi?cmd=search&db=gene&term=S100a8) | S100 calcium binding protein A8 (calgranulin A) |
| 3.00E-06 | 0.0195993 | 108.908 | 15.29444 | 7.1 | [1451699_at](https://www.affymetrix.com/LinkServlet?probeset=1451699_at) | [EG668468](http://www.ncbi.nlm.nih.gov/entrez/query.fcgi?cmd=search&db=gene&term=EG668468) | predicted gene, EG668468 |
| 0.00801 | 0.1595038 | 108.8368 | 15.91068 | 6.8 | [1422352_at](https://www.affymetrix.com/LinkServlet?probeset=1422352_at) | [Mcpt1](http://www.ncbi.nlm.nih.gov/entrez/query.fcgi?cmd=search&db=gene&term=Mcpt1) | mast cell protease 1 |
| 0.00881 | 0.1595038 | 903.1536 | 148.3402 | 6.1 | [1435989_x_at](https://www.affymetrix.com/LinkServlet?probeset=1435989_x_at) | [Krt8](http://www.ncbi.nlm.nih.gov/entrez/query.fcgi?cmd=search&db=gene&term=Krt8) | keratin 8 |
| 0.02476 | 0.1681001 | 920.0291 | 153.7837 | 6.0 | [1417156_at](https://www.affymetrix.com/LinkServlet?probeset=1417156_at) | [Krt19](http://www.ncbi.nlm.nih.gov/entrez/query.fcgi?cmd=search&db=gene&term=Krt19) | keratin 19 |
| 0.01244 | 0.1595038 | 994.1158 | 167.1261 | 5.9 | [1423691_x_at](https://www.affymetrix.com/LinkServlet?probeset=1423691_x_at) | [Krt8](http://www.ncbi.nlm.nih.gov/entrez/query.fcgi?cmd=search&db=gene&term=Krt8) | keratin 8 |
| 0.01272 | 0.1595038 | 931.09 | 157.3257 | 5.9 | [1420647_a_at](https://www.affymetrix.com/LinkServlet?probeset=1420647_a_at) | [Krt8](http://www.ncbi.nlm.nih.gov/entrez/query.fcgi?cmd=search&db=gene&term=Krt8) | keratin 8 |
| 0.02356 | 0.1681001 | 175.6169 | 31.32537 | 5.6 | [1454264_at](https://www.affymetrix.com/LinkServlet?probeset=1454264_at) | [2310046K23Rik](http://www.ncbi.nlm.nih.gov/entrez/query.fcgi?cmd=search&db=gene&term=2310046K23Rik) | RIKEN cDNA 2310046K23 gene |
| 0.00865 | 0.1595038 | 161.7278 | 31.35349 | 5.2 | [1426284_at](https://www.affymetrix.com/LinkServlet?probeset=1426284_at) | [Krt20](http://www.ncbi.nlm.nih.gov/entrez/query.fcgi?cmd=search&db=gene&term=Krt20) | keratin 20 |
| 0.04099 | 0.1847341 | 578.6935 | 118.6265 | 4.9 | [1428909_at](https://www.affymetrix.com/LinkServlet?probeset=1428909_at) | [A130040M12Rik](http://www.ncbi.nlm.nih.gov/entrez/query.fcgi?cmd=search&db=gene&term=A130040M12Rik) | RIKEN cDNA A130040M12 gene |
| 0.0244 | 0.1681001 | 529.3296 | 111.0712 | 4.8 | [1448169_at](https://www.affymetrix.com/LinkServlet?probeset=1448169_at) | [Krt18](http://www.ncbi.nlm.nih.gov/entrez/query.fcgi?cmd=search&db=gene&term=Krt18) | keratin 18 |
| 0.01267 | 0.1595038 | 73.18124 | 16.71039 | 4.4 | [1420579_s_at](https://www.affymetrix.com/LinkServlet?probeset=1420579_s_at) | [Cftr](http://www.ncbi.nlm.nih.gov/entrez/query.fcgi?cmd=search&db=gene&term=Cftr) | cystic fibrosis transmembrane conductance regulator homolog |
| 0.04267 | 0.1875606 | 1199.752 | 276.3356 | 4.3 | [1427700_x_at](https://www.affymetrix.com/LinkServlet?probeset=1427700_x_at) | [Krt6a](http://www.ncbi.nlm.nih.gov/entrez/query.fcgi?cmd=search&db=gene&term=Krt6a) | keratin 6A |
| 0.00613 | 0.1595038 | 76.98058 | 18.10944 | 4.3 | [1449254_at](https://www.affymetrix.com/LinkServlet?probeset=1449254_at) | [Spp1](http://www.ncbi.nlm.nih.gov/entrez/query.fcgi?cmd=search&db=gene&term=Spp1) | secreted phosphoprotein 1 |
| 0.01681 | 0.1616151 | 38.81779 | 9.312791 | 4.2 | [1430443_at](https://www.affymetrix.com/LinkServlet?probeset=1430443_at) | [Anxa10](http://www.ncbi.nlm.nih.gov/entrez/query.fcgi?cmd=search&db=gene&term=Anxa10) | annexin A10 |
| 0.0349 | 0.1769879 | 339.3368 | 83.20553 | 4.1 | [1427660_x_at](https://www.affymetrix.com/LinkServlet?probeset=1427660_x_at) | [Igk-V1](http://www.ncbi.nlm.nih.gov/entrez/query.fcgi?cmd=search&db=gene&term=Igk-V1) | immunoglobulin kappa chain variable 1 (V1) |
| 0.01743 | 0.1616151 | 161.0008 | 42.17846 | 3.8 | [1452426_x_at](https://www.affymetrix.com/LinkServlet?probeset=1452426_x_at) | [NA](http://www.ncbi.nlm.nih.gov/entrez/query.fcgi?cmd=search&db=gene&term=NA) | NA |
| 0.01447 | 0.15971 | 168.3152 | 44.35533 | 3.8 | [1434046_at](https://www.affymetrix.com/LinkServlet?probeset=1434046_at) | [AA467197](http://www.ncbi.nlm.nih.gov/entrez/query.fcgi?cmd=search&db=gene&term=AA467197) | expressed sequence AA467197 |
| 0.04351 | 0.1883436 | 703.6904 | 187.8484 | 3.7 | [1423227_at](https://www.affymetrix.com/LinkServlet?probeset=1423227_at) | [Krt17](http://www.ncbi.nlm.nih.gov/entrez/query.fcgi?cmd=search&db=gene&term=Krt17) | keratin 17 |
| 0.04193 | 0.1866266 | 308.7244 | 84.37923 | 3.7 | [1418724_at](https://www.affymetrix.com/LinkServlet?probeset=1418724_at) | [Cfi](http://www.ncbi.nlm.nih.gov/entrez/query.fcgi?cmd=search&db=gene&term=Cfi) | complement component factor i |
| 0.02481 | 0.1681001 | 277.3724 | 76.88514 | 3.6 | [1427455_x_at](https://www.affymetrix.com/LinkServlet?probeset=1427455_x_at) | [Igk-V1](http://www.ncbi.nlm.nih.gov/entrez/query.fcgi?cmd=search&db=gene&term=Igk-V1) | immunoglobulin kappa chain variable 1 (V1) |
| 0.00395 | 0.1595038 | 86.34564 | 24.41521 | 3.5 | [1448393_at](https://www.affymetrix.com/LinkServlet?probeset=1448393_at) | [Cldn7](http://www.ncbi.nlm.nih.gov/entrez/query.fcgi?cmd=search&db=gene&term=Cldn7) | claudin 7 |
| 0.04575 | 0.1907595 | 418.808 | 125.8552 | 3.3 | [1449199_at](https://www.affymetrix.com/LinkServlet?probeset=1449199_at) | [Muc1](http://www.ncbi.nlm.nih.gov/entrez/query.fcgi?cmd=search&db=gene&term=Muc1) | mucin 1, transmembrane |
| 0.04652 | 0.1913644 | 85.60029 | 26.76494 | 3.2 | [1449896_at](https://www.affymetrix.com/LinkServlet?probeset=1449896_at) | [Mlph](http://www.ncbi.nlm.nih.gov/entrez/query.fcgi?cmd=search&db=gene&term=Mlph) | melanophilin |
| 0.02159 | 0.1638291 | 90.27983 | 29.18104 | 3.1 | [1425445_a_at](https://www.affymetrix.com/LinkServlet?probeset=1425445_a_at) | [Cldn18](http://www.ncbi.nlm.nih.gov/entrez/query.fcgi?cmd=search&db=gene&term=Cldn18) | claudin 18 |
| 0.02163 | 0.1638291 | 27.48522 | 9.024961 | 3.0 | [1425324_x_at](https://www.affymetrix.com/LinkServlet?probeset=1425324_x_at) | [Igh-6](http://www.ncbi.nlm.nih.gov/entrez/query.fcgi?cmd=search&db=gene&term=Igh-6) | immunoglobulin heavy chain 6 (heavy chain of IgM) |
| 0.00422 | 0.1595038 | 811.3555 | 270.5727 | 3.0 | [1435137_s_at](https://www.affymetrix.com/LinkServlet?probeset=1435137_s_at) | [1200016E24Rik](http://www.ncbi.nlm.nih.gov/entrez/query.fcgi?cmd=search&db=gene&term=1200016E24Rik) | RIKEN cDNA 1200016E24 gene |
| 0.02836 | 0.1703848 | 362.955 | 121.7103 | 3.0 | [1457666_s_at](https://www.affymetrix.com/LinkServlet?probeset=1457666_s_at) | [Ifi202b](http://www.ncbi.nlm.nih.gov/entrez/query.fcgi?cmd=search&db=gene&term=Ifi202b) | interferon activated gene 202B |
| 0.03567 | 0.1777257 | 365.0299 | 123.4933 | 3.0 | [1421551_s_at](https://www.affymetrix.com/LinkServlet?probeset=1421551_s_at) | [Ifi202b](http://www.ncbi.nlm.nih.gov/entrez/query.fcgi?cmd=search&db=gene&term=Ifi202b) | interferon activated gene 202B |
| 0.04114 | 0.1850213 | 84.26685 | 28.53256 | 3.0 | [1449409_at](https://www.affymetrix.com/LinkServlet?probeset=1449409_at) | [Sult1c2](http://www.ncbi.nlm.nih.gov/entrez/query.fcgi?cmd=search&db=gene&term=Sult1c2) | sulfotransferase family, cytosolic, 1C, member 2 |
| 0.00292 | 0.1595038 | 92.94745 | 31.64863 | 2.9 | [1420378_at](https://www.affymetrix.com/LinkServlet?probeset=1420378_at) | [Sftpd](http://www.ncbi.nlm.nih.gov/entrez/query.fcgi?cmd=search&db=gene&term=Sftpd) | surfactant associated protein D |
| 0.04241 | 0.1875606 | 131.0311 | 45.81718 | 2.9 | [1417231_at](https://www.affymetrix.com/LinkServlet?probeset=1417231_at) | [Cldn2](http://www.ncbi.nlm.nih.gov/entrez/query.fcgi?cmd=search&db=gene&term=Cldn2) | claudin 2 |
| 0.01999 | 0.1632196 | 20.79848 | 7.545389 | 2.8 | [1430234_at](https://www.affymetrix.com/LinkServlet?probeset=1430234_at) | [Arl14](http://www.ncbi.nlm.nih.gov/entrez/query.fcgi?cmd=search&db=gene&term=Arl14) | ADP-ribosylation factor-like 14 |
| 0.00208 | 0.1595038 | 21.02593 | 7.656573 | 2.7 | [1421775_at](https://www.affymetrix.com/LinkServlet?probeset=1421775_at) | [Fcer1a](http://www.ncbi.nlm.nih.gov/entrez/query.fcgi?cmd=search&db=gene&term=Fcer1a) | Fc receptor, IgE, high affinity I, alpha polypeptide |
| 0.019 | 0.162422 | 166.0885 | 60.66572 | 2.7 | [1416596_at](https://www.affymetrix.com/LinkServlet?probeset=1416596_at) | [Slc44a4](http://www.ncbi.nlm.nih.gov/entrez/query.fcgi?cmd=search&db=gene&term=Slc44a4) | solute carrier family 44, member 4 |
| 0.02025 | 0.1636719 | 63.18777 | 23.15257 | 2.7 | [1425247_a_at](https://www.affymetrix.com/LinkServlet?probeset=1425247_a_at) | [Igh-6](http://www.ncbi.nlm.nih.gov/entrez/query.fcgi?cmd=search&db=gene&term=Igh-6) | immunoglobulin heavy chain 6 (heavy chain of IgM) |
| 0.03278 | 0.1737573 | 153.0124 | 57.03326 | 2.7 | [1417957_a_at](https://www.affymetrix.com/LinkServlet?probeset=1417957_a_at) | [Tspan1](http://www.ncbi.nlm.nih.gov/entrez/query.fcgi?cmd=search&db=gene&term=Tspan1) | tetraspanin 1 |
| 0.01221 | 0.1595038 | 1740.466 | 662.4769 | 2.6 | [1427932_s_at](https://www.affymetrix.com/LinkServlet?probeset=1427932_s_at) | [1200016E24Rik](http://www.ncbi.nlm.nih.gov/entrez/query.fcgi?cmd=search&db=gene&term=1200016E24Rik) | RIKEN cDNA 1200016E24 gene |
| 0.01324 | 0.1595038 | 117.7453 | 45.39548 | 2.6 | [1420017_at](https://www.affymetrix.com/LinkServlet?probeset=1420017_at) | [Tspan8](http://www.ncbi.nlm.nih.gov/entrez/query.fcgi?cmd=search&db=gene&term=Tspan8) | tetraspanin 8 |
| 0.02877 | 0.1708531 | 80.65946 | 31.71467 | 2.5 | [1451498_at](https://www.affymetrix.com/LinkServlet?probeset=1451498_at) | [Lrrc26](http://www.ncbi.nlm.nih.gov/entrez/query.fcgi?cmd=search&db=gene&term=Lrrc26) | leucine rich repeat containing 26 |
| 0.04942 | 0.1944922 | 123.7176 | 49.34032 | 2.5 | [1418818_at](https://www.affymetrix.com/LinkServlet?probeset=1418818_at) | [Aqp5](http://www.ncbi.nlm.nih.gov/entrez/query.fcgi?cmd=search&db=gene&term=Aqp5) | aquaporin 5 |
| 0.01088 | 0.1595038 | 1358.106 | 543.9257 | 2.5 | [1453238_s_at](https://www.affymetrix.com/LinkServlet?probeset=1453238_s_at) | [1200016E24Rik](http://www.ncbi.nlm.nih.gov/entrez/query.fcgi?cmd=search&db=gene&term=1200016E24Rik) | RIKEN cDNA 1200016E24 gene |
| 0.04329 | 0.188045 | 36.01363 | 14.46607 | 2.5 | [1448837_at](https://www.affymetrix.com/LinkServlet?probeset=1448837_at) | [Vil1](http://www.ncbi.nlm.nih.gov/entrez/query.fcgi?cmd=search&db=gene&term=Vil1) | villin 1 |
| 0.04553 | 0.190714 | 25.23996 | 10.19925 | 2.5 | [1430963_at](https://www.affymetrix.com/LinkServlet?probeset=1430963_at) | [Gcnt3](http://www.ncbi.nlm.nih.gov/entrez/query.fcgi?cmd=search&db=gene&term=Gcnt3) | glucosaminyl (N-acetyl) transferase 3, mucin type |
| 0.03972 | 0.1824798 | 96.95114 | 39.1967 | 2.5 | [1418405_at](https://www.affymetrix.com/LinkServlet?probeset=1418405_at) | [Hgfac](http://www.ncbi.nlm.nih.gov/entrez/query.fcgi?cmd=search&db=gene&term=Hgfac) | hepatocyte growth factor activator |
| 0.03443 | 0.1759329 | 47.51821 | 19.3955 | 2.4 | [1450455_s_at](https://www.affymetrix.com/LinkServlet?probeset=1450455_s_at) | [Akr1c12](http://www.ncbi.nlm.nih.gov/entrez/query.fcgi?cmd=search&db=gene&term=Akr1c12) | aldo-keto reductase family 1, member C12 |
| 0.01576 | 0.161347 | 530.9789 | 217.4193 | 2.4 | [1451336_at](https://www.affymetrix.com/LinkServlet?probeset=1451336_at) | [Lgals4](http://www.ncbi.nlm.nih.gov/entrez/query.fcgi?cmd=search&db=gene&term=Lgals4) | lectin, galactose binding, soluble 4 |
| 0.03802 | 0.1805454 | 24.04396 | 10.18893 | 2.4 | [1439489_at](https://www.affymetrix.com/LinkServlet?probeset=1439489_at) | [Gpr120](http://www.ncbi.nlm.nih.gov/entrez/query.fcgi?cmd=search&db=gene&term=Gpr120) | G protein-coupled receptor 120 |
| 0.03983 | 0.1827511 | 71.32248 | 30.53238 | 2.3 | [1451139_at](https://www.affymetrix.com/LinkServlet?probeset=1451139_at) | [Slc39a4](http://www.ncbi.nlm.nih.gov/entrez/query.fcgi?cmd=search&db=gene&term=Slc39a4) | solute carrier family 39 (zinc transporter), member 4 |
| 0.04968 | 0.1946372 | 63.91141 | 27.98181 | 2.3 | [1448789_at](https://www.affymetrix.com/LinkServlet?probeset=1448789_at) | [Aldh1a3](http://www.ncbi.nlm.nih.gov/entrez/query.fcgi?cmd=search&db=gene&term=Aldh1a3) | aldehyde dehydrogenase family 1, subfamily A3 |
| 0.01268 | 0.1595038 | 132.8345 | 59.39859 | 2.2 | [1421134_at](https://www.affymetrix.com/LinkServlet?probeset=1421134_at) | [Areg](http://www.ncbi.nlm.nih.gov/entrez/query.fcgi?cmd=search&db=gene&term=Areg) | amphiregulin |
| 0.01422 | 0.1596356 | 24.02389 | 10.84878 | 2.2 | [1452463_x_at](https://www.affymetrix.com/LinkServlet?probeset=1452463_x_at) | [Igk-V1](http://www.ncbi.nlm.nih.gov/entrez/query.fcgi?cmd=search&db=gene&term=Igk-V1) | immunoglobulin kappa chain variable 1 (V1) |
| 0.02346 | 0.1681001 | 131.7033 | 61.14288 | 2.2 | [1427870_x_at](https://www.affymetrix.com/LinkServlet?probeset=1427870_x_at) | [Igh-6](http://www.ncbi.nlm.nih.gov/entrez/query.fcgi?cmd=search&db=gene&term=Igh-6) | immunoglobulin heavy chain 6 (heavy chain of IgM) |
| 0.02439 | 0.1681001 | 56.12755 | 26.28267 | 2.1 | [1426663_s_at](https://www.affymetrix.com/LinkServlet?probeset=1426663_s_at) | [Slc45a3](http://www.ncbi.nlm.nih.gov/entrez/query.fcgi?cmd=search&db=gene&term=Slc45a3) | solute carrier family 45, member 3 |
| 0.01452 | 0.15971 | 38.90825 | 18.30722 | 2.1 | [1455274_at](https://www.affymetrix.com/LinkServlet?probeset=1455274_at) | [NA](http://www.ncbi.nlm.nih.gov/entrez/query.fcgi?cmd=search&db=gene&term=NA) | NA |
| 0.03909 | 0.181666 | 204.9008 | 97.54187 | 2.1 | [1449994_at](https://www.affymetrix.com/LinkServlet?probeset=1449994_at) | [Epgn](http://www.ncbi.nlm.nih.gov/entrez/query.fcgi?cmd=search&db=gene&term=Epgn) | epithelial mitogen |
| 0.0247 | 0.1681001 | 79.3572 | 38.15931 | 2.1 | [1430479_at](https://www.affymetrix.com/LinkServlet?probeset=1430479_at) | [2010007H06Rik](http://www.ncbi.nlm.nih.gov/entrez/query.fcgi?cmd=search&db=gene&term=2010007H06Rik) | RIKEN cDNA 2010007H06 gene |
| 0.04082 | 0.1847083 | 91.68211 | 44.39735 | 2.1 | [1419148_at](https://www.affymetrix.com/LinkServlet?probeset=1419148_at) | [Avil](http://www.ncbi.nlm.nih.gov/entrez/query.fcgi?cmd=search&db=gene&term=Avil) | advillin |
| 0.02739 | 0.1703848 | 93.351 | 45.26222 | 2.1 | [1439506_at](https://www.affymetrix.com/LinkServlet?probeset=1439506_at) | [Gm98](http://www.ncbi.nlm.nih.gov/entrez/query.fcgi?cmd=search&db=gene&term=Gm98) | gene model 98, (NCBI) |
| 0.0462 | 0.1913644 | 18.34564 | 8.999526 | 2.0 | [1424901_at](https://www.affymetrix.com/LinkServlet?probeset=1424901_at) | [Gcnt3](http://www.ncbi.nlm.nih.gov/entrez/query.fcgi?cmd=search&db=gene&term=Gcnt3) | glucosaminyl (N-acetyl) transferase 3, mucin type |
| 0.03946 | 0.1820123 | 102.4291 | 50.24904 | 2.0 | [1424339_at](https://www.affymetrix.com/LinkServlet?probeset=1424339_at) | [Oasl1](http://www.ncbi.nlm.nih.gov/entrez/query.fcgi?cmd=search&db=gene&term=Oasl1) | oligoadenylate synthetase-like 1 |
| 0.03284 | 0.1737573 | 191.1815 | 96.29854 | 2.0 | [1451610_at](https://www.affymetrix.com/LinkServlet?probeset=1451610_at) | [Cxcl17](http://www.ncbi.nlm.nih.gov/entrez/query.fcgi?cmd=search&db=gene&term=Cxcl17) | chemokine (C-X-C motif) ligand 17 |
| 0.02726 | 0.1703848 | 45.00859 | 22.68697 | 2.0 | [1422211_a_at](https://www.affymetrix.com/LinkServlet?probeset=1422211_a_at) | [B3gnt3](http://www.ncbi.nlm.nih.gov/entrez/query.fcgi?cmd=search&db=gene&term=B3gnt3) | UDP-GlcNAc:betaGal beta-1,3-N-acetylglucosaminyltransferase 3 |
| 0.02777 | 0.1703848 | 149.7346 | 76.52136 | 2.0 | [1454254_s_at](https://www.affymetrix.com/LinkServlet?probeset=1454254_s_at) | [1600029D21Rik](http://www.ncbi.nlm.nih.gov/entrez/query.fcgi?cmd=search&db=gene&term=1600029D21Rik) | RIKEN cDNA 1600029D21 gene |
| 0.02054 | 0.1638291 | 232.4757 | 119.0402 | 2.0 | [1423933_a_at](https://www.affymetrix.com/LinkServlet?probeset=1423933_a_at) | [1600029D21Rik](http://www.ncbi.nlm.nih.gov/entrez/query.fcgi?cmd=search&db=gene&term=1600029D21Rik) | RIKEN cDNA 1600029D21 gene |
| 4.51E-05 | 0.1020615 | 14.35825 | 53.2151 | 0.27 | [1456084_x_at](https://www.affymetrix.com/LinkServlet?probeset=1456084_x_at) | [Fmod](http://www.ncbi.nlm.nih.gov/entrez/query.fcgi?cmd=search&db=gene&term=Fmod) | fibromodulin |
| 0.0016 | 0.1539827 | 253.0213 | 762.6098 | 0.33 | [1452107_s_at](https://www.affymetrix.com/LinkServlet?probeset=1452107_s_at) | [NA](http://www.ncbi.nlm.nih.gov/entrez/query.fcgi?cmd=search&db=gene&term=NA) | NA |
| 0.0165 | 0.1616151 | 25.14543 | 75.67462 | 0.33 | [1460118_at](https://www.affymetrix.com/LinkServlet?probeset=1460118_at) | [NA](http://www.ncbi.nlm.nih.gov/entrez/query.fcgi?cmd=search&db=gene&term=NA) | NA |
| 5.40E-06 | 0.0195993 | 11.30382 | 31.73836 | 0.36 | [1443639_at](https://www.affymetrix.com/LinkServlet?probeset=1443639_at) | [Apcdd1](http://www.ncbi.nlm.nih.gov/entrez/query.fcgi?cmd=search&db=gene&term=Apcdd1) | adenomatosis polyposis coli down-regulated 1 |
| 0.00653 | 0.1595038 | 51.45139 | 134.8271 | 0.38 | [1449314_at](https://www.affymetrix.com/LinkServlet?probeset=1449314_at) | [Zfpm2](http://www.ncbi.nlm.nih.gov/entrez/query.fcgi?cmd=search&db=gene&term=Zfpm2) | zinc finger protein, multitype 2 |
| 0.00098 | 0.1539827 | 791.9122 | 2016.273 | 0.39 | [1452106_at](https://www.affymetrix.com/LinkServlet?probeset=1452106_at) | [Npnt](http://www.ncbi.nlm.nih.gov/entrez/query.fcgi?cmd=search&db=gene&term=Npnt) | nephronectin |
| 0.01046 | 0.1595038 | 31.82941 | 78.00416 | 0.41 | [1434264_at](https://www.affymetrix.com/LinkServlet?probeset=1434264_at) | [Ank2](http://www.ncbi.nlm.nih.gov/entrez/query.fcgi?cmd=search&db=gene&term=Ank2) | ankyrin 2, brain |
| 0.00494 | 0.1595038 | 210.5027 | 490.5223 | 0.43 | [1447830_s_at](https://www.affymetrix.com/LinkServlet?probeset=1447830_s_at) | [Rgs2](http://www.ncbi.nlm.nih.gov/entrez/query.fcgi?cmd=search&db=gene&term=Rgs2) | regulator of G-protein signaling 2 |
| 0.00629 | 0.1595038 | 105.9028 | 236.154 | 0.45 | [1428948_at](https://www.affymetrix.com/LinkServlet?probeset=1428948_at) | [5730414M22Rik](http://www.ncbi.nlm.nih.gov/entrez/query.fcgi?cmd=search&db=gene&term=5730414M22Rik) | RIKEN cDNA 5730414M22 gene |
| 0.00456 | 0.1595038 | 58.15856 | 124.9872 | 0.47 | [1420994_at](https://www.affymetrix.com/LinkServlet?probeset=1420994_at) | [B3gnt5](http://www.ncbi.nlm.nih.gov/entrez/query.fcgi?cmd=search&db=gene&term=B3gnt5) | UDP-GlcNAc:betaGal beta-1,3-N-acetylglucosaminyltransferase 5 |
| 0.01075 | 0.1595038 | 163.849 | 348.0996 | 0.47 | [1420514_at](https://www.affymetrix.com/LinkServlet?probeset=1420514_at) | [Tmem47](http://www.ncbi.nlm.nih.gov/entrez/query.fcgi?cmd=search&db=gene&term=Tmem47) | transmembrane protein 47 |
| 0.00572 | 0.1595038 | 347.59 | 735.4929 | 0.47 | [1419248_at](https://www.affymetrix.com/LinkServlet?probeset=1419248_at) | [Rgs2](http://www.ncbi.nlm.nih.gov/entrez/query.fcgi?cmd=search&db=gene&term=Rgs2) | regulator of G-protein signaling 2 |
| 0.00481 | 0.1595038 | 627.4577 | 1312.292 | 0.48 | [1455900_x_at](https://www.affymetrix.com/LinkServlet?probeset=1455900_x_at) | [Tgm2](http://www.ncbi.nlm.nih.gov/entrez/query.fcgi?cmd=search&db=gene&term=Tgm2) | transglutaminase 2, C polypeptide |
| 0.02099 | 0.1638291 | 174.7134 | 355.9213 | 0.49 | [1429506_at](https://www.affymetrix.com/LinkServlet?probeset=1429506_at) | [Nkd1](http://www.ncbi.nlm.nih.gov/entrez/query.fcgi?cmd=search&db=gene&term=Nkd1) | naked cuticle 1 homolog (Drosophila) |
| 0.00065 | 0.1539827 | 17.37056 | 35.19989 | 0.49 | [1458345_s_at](https://www.affymetrix.com/LinkServlet?probeset=1458345_s_at) | [Colec11](http://www.ncbi.nlm.nih.gov/entrez/query.fcgi?cmd=search&db=gene&term=Colec11) | collectin sub-family member 11 |
| 0.00473 | 0.1595038 | 88.71397 | 179.2957 | 0.49 | [1433783_at](https://www.affymetrix.com/LinkServlet?probeset=1433783_at) | [Ldb3](http://www.ncbi.nlm.nih.gov/entrez/query.fcgi?cmd=search&db=gene&term=Ldb3) | LIM domain binding 3 |
| 0.00146 | 0.1539827 | 37.22087 | 75.05295 | 0.50 | [1446127_at](https://www.affymetrix.com/LinkServlet?probeset=1446127_at) | [Zeb1](http://www.ncbi.nlm.nih.gov/entrez/query.fcgi?cmd=search&db=gene&term=Zeb1) | zinc finger E-box binding homeobox 1 |
| 0.00387 | 0.1595038 | 231.2416 | 462.2831 | 0.50 | [1428739_at](https://www.affymetrix.com/LinkServlet?probeset=1428739_at) | [2310040A07Rik](http://www.ncbi.nlm.nih.gov/entrez/query.fcgi?cmd=search&db=gene&term=2310040A07Rik) | RIKEN cDNA 2310040A07 gene |
| 0.03516 | 0.1773902 | 48.51211 | 96.84425 | 0.50 | [1440884_s_at](https://www.affymetrix.com/LinkServlet?probeset=1440884_s_at) | [A530047J11Rik](http://www.ncbi.nlm.nih.gov/entrez/query.fcgi?cmd=search&db=gene&term=A530047J11Rik) | RIKEN cDNA A530047J11 gene |
| 6.24E-05 | 0.1020615 | 34.47411 | 68.69629 | 0.50 | [1426561_a_at](https://www.affymetrix.com/LinkServlet?probeset=1426561_a_at) | [Npnt](http://www.ncbi.nlm.nih.gov/entrez/query.fcgi?cmd=search&db=gene&term=Npnt) | nephronectin |
| 0.01096 | 0.1595038 | 60.63977 | 120.4877 | 0.50 | [1455298_at](https://www.affymetrix.com/LinkServlet?probeset=1455298_at) | [NA](http://www.ncbi.nlm.nih.gov/entrez/query.fcgi?cmd=search&db=gene&term=NA) | NA |
| 0.01864 | 0.1616151 | 90.05085 | 178.7914 | 0.50 | [1424797_a_at](https://www.affymetrix.com/LinkServlet?probeset=1424797_a_at) | [Pitx2](http://www.ncbi.nlm.nih.gov/entrez/query.fcgi?cmd=search&db=gene&term=Pitx2) | paired-like homeodomain transcription factor 2 |
