# Supplementary material for: Zinc deficiency activates S100A8 inflammation in the absence of COX-2 and promotes murine oral-esophageal tumor progression
Source: Int J Cancer. 2010 Sep 20;129(2):331–45. doi: 10.1002/ijc.25688 (PMC3015018; doi:10.1002/ijc.25688)
Supplement: Supplementary file 6 [file ijc0129-0331-SD6.doc]

**Supporting Information Table 5.** Gene expression profile analysis of ZS:*Cox-2^-/-^* *vs* ZS:WT mouse forestomach

**Description of the problem:**

Number of classes: 2

Number of genes used for random variance estimation: 45101

Number of genes that passed filtering criteria: 7259

Type of univariate test used: Two-sample T-test (with random variance model)

Column of the Experiment Descriptors sheet that defines class variable : ZS-- vs ZS++ (ZS:*Cox-2^-/-^* versus ZS:WT)

Univariate test random variance model parameters: a= 1.36024, b= 19.06294, Kolmogorov-Smirnov statistic= 0.01423

ZD = zinc-deficient; ZS = zinc-sufficient

Nominal significance level of each univariate test: 0.05

**Summary of Results**:

**Number of genes significant at 0.05 level of the univariate test: 479**

**Number of genes significant at 0.05 level and with a cut-off point of 2-fold or more difference: 17**

**Genes which discriminate among classes**:

Table - Sorted by p-value of the univariate test and a cut-off point of 2-fold or more difference

Class 1: ZS--; Class 2: ZS++.

Up-regulated genes are in purple, down-regulated genes are in blue (**11** up-regulated and **6** down-regulated)

| **p-value** | **FDR** | **ZS--** | **ZS++** | **Fold-change** | **Probe set** | **Gene symbol** | **Description** |
| --- | --- | --- | --- | --- | --- | --- | --- |
| 0.0075289 | 0.7375301 | 412.2909076 | 136.7875285 | 3.0 | 1453279_x_at | Krt76 | keratin 76 |
| 0.0061829 | 0.7375301 | 90.0115587 | 32.5066866 | 2.8 | 1457843_at | Lypd6 | LY6/PLAUR domain containing 6 |
| 0.0272493 | 0.7375301 | 154.0499954 | 63.8770649 | 2.4 | 1435621_at | Far2 | fatty acyl CoA reductase 2 |
| 0.0116122 | 0.7375301 | 267.2154929 | 122.4638541 | 2.2 | 1451551_at | Krt84 | keratin 84 |
| 0.0298578 | 0.7375301 | 71.5636814 | 33.253908 | 2.2 | 1454254_s_at | 1600029D21Rik | RIKEN cDNA 1600029D21 gene |
| 0.00603 | 0.7375301 | 173.1771643 | 81.4006771 | 2.1 | 1445332_at | NA | NA |
| 1.80E-05 | 0.130662 | 22.0414956 | 10.4764348 | 2.1 | 1436291_a_at | Dpys | dihydropyrimidinase |
| 0.0188612 | 0.7375301 | 121.1354961 | 58.899246 | 2.1 | 1458680_at | NA | NA |
| 0.0290923 | 0.7375301 | 90.6551198 | 44.8465619 | 2.0 | 1431253_s_at | Tbc1d9 | TBC1 domain family, member 9 |
| 0.0015094 | 0.7375301 | 38.5171612 | 19.5642637 | 2.0 | 1419560_at | Lipc | lipase, hepatic |
| 0.0081856 | 0.7375301 | 218.0095621 | 110.9780053 | 2.0 | 1455679_at | NA | NA |
| 0.0322393 | 0.7375301 | 126.8977642 | 413.3064799 | 0.31 | 1417732_at | Anxa8 | annexin A8 |
| 0.0176545 | 0.7375301 | 42.544807 | 126.5207527 | 0.34 | 1452426_x_at | NA | NA |
| 0.0007404 | 0.7375301 | 9.3215903 | 26.7160213 | 0.35 | 1443639_at | Apcdd1 | adenomatosis polyposis coli down-regulated 1 |
| 0.0297497 | 0.7375301 | 20.0936253 | 42.2499984 | 0.48 | 1448789_at | Aldh1a3 | aldehyde dehydrogenase family 1, subfamily A3 |
| 0.0181709 | 0.7375301 | 31.9471555 | 63.8004776 | 0.50 | 1441531_at | LOC100043487 | hypothetical protein LOC100043487 |
| 0.0196067 | 0.7375301 | 25.1137992 | 49.8945802 | 0.50 | 1450723_at | Isl1 | ISL1 transcription factor, LIM/homeodomain |
