# Supplementary material for: Zinc deficiency activates S100A8 inflammation in the absence of COX-2 and promotes murine oral-esophageal tumor progression
Source: Int J Cancer. 2010 Sep 20;129(2):331–45. doi: 10.1002/ijc.25688 (PMC3015018; doi:10.1002/ijc.25688)
Supplement: Supplementary file 7 [file ijc0129-0331-SD7.doc]

Supporting Information Table 6. Real-time qRT-PCR validation of array data

| Gene symbol | Fold-change | |
| --- | --- | --- |
|  | Microarray data | qRT-PCR  Mean ± SEM; n= 6 samples |
|  | **ZD:Cox-2^-/-^ vs ZS:Cox-2^-/-^ mouse forestomach** | |
| *S100a8*  *S100a9*  *Sprr2h*  *Sprr2f*  *Spp1*  *Mcpt1*  *Dmbt1*  *Krt16*  *Krt18*  *Krt19*  *Anxa10*  *Cyp1b1* | 24  2.2  64  49  7.9  22  27  47  7.9  8.9  8.2  0.2  (from Table 1) | 46 ± 15  5.4 ± 1.6  91 ± 25  43 ± 15  29 ± 18  36 ± 13  83 ±3 5  50 ±1 2  7.7 ± 1.7  9.7 ± 3.5  12 ± 3.7  0.3 ± 0.1 |
|  | **ZD:WT vs ZS:WT mouse forestomach** | |
|  |  |  |
| *S100a8* | 4.2 | 6.9 ± 2.8 |
| *S100a9* | 2.4 | 2.4 ± 0.7 |
| *Sprr2h* | 8.4 | 22 ± 12 |
| *Sprr2f* | 9.1 | 14 ± 8.1 |
| *Dmbt1* | 4.5 | 19 ± 8.5 |
| *Krt19* | 3.7 | 8.7 ± 3.8 |
|  | (from Supporting InformationTable 3) |  |
|  | **ZD:*Cox-2^-/-^ vs* ZD:WT mouse forestomach** | |
| *S100a8* | 7.5 | 11 ± 4.4 |
| *Mcpt1* | 6.8 | 19 ± 7.3 |
| *Dmbt1* | 8.7 | 8.3 ± 3.4 |
| *Krt16* | 9.1 | 13 ± 5.3 |
| *Krt18* | 4.8 | 4.4 ± 1.8 |
| *Krt19* | 6.0 | 6.7 ± 2.7 |
| *Anxa10* | 4.2 | 4.6 ± 1.6 |
|  | (from Supporting Information Table 4) |  |
|  | **ZS:*Cox-2^-/-^ vs* ZS:WT mouse forestomach** | |
| *Far2* | 2.4 | 1.4 ± 0.2 |
| *Dpys* | 2.1 | 10 ± 1.4 |
| *Anxa8* | 0.30 | 0.2 ± 0.1 |
|  | (from Supporting Information Table 5) |  |
