# Supplementary material for: Zinc deficiency activates S100A8 inflammation in the absence of COX-2 and promotes murine oral-esophageal tumor progression
Source: Int J Cancer. 2010 Sep 20;129(2):331–45. doi: 10.1002/ijc.25688 (PMC3015018; doi:10.1002/ijc.25688)
Supplement: Supplementary file 8 [file ijc0129-0331-SD8.doc]

**Supporting Information Table 7**. Identification of significantly overrepresented biological processes by DAVID bioinformatics in

**a**) ZD:*Cox-2*^-/-^ *vs* ZS:*Cox-2*^-/-^, **b**) ZD:WT *vs* ZS:WT, **c**) ZD:*Cox-2*^-/-^ *vs* ZD:WT, **d**) ZS:*Cox-2*^-/-^ *vs* ZS:WT mouse forestomachs

| **Functional Group 1** | **Median p-value p-value: 4.06E-5** | ***p-value*** | ***Genes*** |
| --- | --- | --- | --- |
|  | Cell Communication | 1.39E-009 | Krt16, Krt19, Krt20, Krt8, Krt6b, Krt18, Dsc2, Krt76, Spp1, Gjb1, Krt6a, Krt7, Chad, Krt17, |
|  | cytoskeleton | 1.21E-006 | Krt19, Kif17, Vil1, Ndn, Myh1, Ank2, Krt8, Sprr2h, Krt18, Add2, Krt76, Sprr2k, Krt6a, Krt7, Krt16, Mlph, Krt20,  Myo1a, Stom, Muc1, Clic5, Sprr2f, Sprr2d, Krt6b, Sprr1b, Avil, Dsc2, Lrrc26, Krt17, |
|  | intermediate filament | 5.50E-006 | Krt16, Krt18, Krt19, Krt20, Krt76, Krt6a, Krt7, Krt8, Krt6b, Krt17, |
|  | morphogenesis of an epithelium | 0.01 | Krt6a, Aldh1a3, Dmbt1, Car9, Krt6b, Krt17, |
| **Functional Group 2** | **Median p-value: 0.005** | ***p-value*** | ***Genes*** |
|  | epidermal cell differentiation | 3.66E-009 | Sprr1b, Sprr2h, Keap1, Sprr2k, Krt6a, Sprr2f, Sprr2d, Krt6b, Krt17, |
|  | cornified envelope | 4.84E-005 | Sprr1b, Sprr2h, Sprr2k, Sprr2f, Sprr2d, |
|  | tissue development | 0.01 | Sprr1b, Sprr2h, Keap1, Sprr2k, Spp1, Krt6a, Sprr2f, Sprr2d, Krt6b, Krt17, |
|  | multi-organism process | 0.01 | Sprr2h, Defb4, Tgtp, Sprr2k, Sprr2f, Sprr2d, Defb3, Krt8, |
| **Functional Group 3** | **Median p-value: 0.009** | ***p-value*** | ***Genes*** |
|  | response to external stimulus | 3.98E-004 | Fabp4, F5, Cxcl17, Reg3g, Chi3l3, S100a9, Sftpd, Fcer1a, Spp1, S100a8, Hbegf, Cfi, F3, Ghrl, Chi3l4, Anxa8, |
|  | response to stimulus | 5.40E-004 | Oasl1, F5, Cxcl17, Ahrr, Pmaip1, Reg3g, Runx1, Defb3, S100a9, Krt8, Sftpd, Fcer1a, Defb4, Penk1, S100a8,  Igh-6, Ppbp, Cfi, Ifi205, Anxa8, Fabp4, Tcrb-J, Krt20, Akr1c12, Tgtp, Chi3l3, Avil, Spp1, Igj, Hbegf, Gbp2, Ern2,  F3, Ifi202b, Ghrl, Chi3l4, |
|  | defense response | 0.02 | Sftpd, Fabp4, Tcrb-J, Fcer1a, Defb4, Penk1, Spp1, Reg3g, Defb3, Cfi, Chi3l3, Chi3l4, |
|  | response to stress | 0.02 | Fabp4, F5, Krt20, Pmaip1, Reg3g, Runx1, Chi3l3, Avil, Fcer1a, Penk1, Spp1, Hbegf, Ern2, Cfi, F3, Chi3l4, Anxa8, |
| **Functional Group 4** | **Median p-value: 0.02** | ***p-value*** | ***Genes*** |
|  | anatomical structure morphogenesis | 0.01 | Cxcl17, Slit3, Ndn, Runx1, Dmbt1, Krt8, Gata6, Sprr2h, Sprr2k, Krt6a, Zfpm2, Epgn, Myo1a, Sprr2d, Sprr2f, Clic5,  Krt6b, Sprr1b, Keap1, Alcam, Hbegf, Pitx2, Aldh1a3, Car9, Ghrl, Krt17, |
|  | organ development | 0.01 | Krt19, Cxcl17, Slit3, Myh1, Runx1, Gata6, Sftpd, Sprr2h, Sprr2k, Add2, Pkp2, Krt6a, Zfpm2, Epgn, Sprr2d, Sprr2f,  Clic5, Krt6b, Sprr1b, Keap1, Sim2, Spp1, Hbegf, Pitx2, Aldh1a3, Krt17, |
|  | multicellular organismal process | 0.02 | Krt19, F5, Slit3, Cxcl17, Ndn, Runx1, Myh1, Dmbt1, Krt8, Gata6, Sftpd, Sprr2h, Fcer1a, Penk1, Add2, Sprr2k,  Igh-6, Pkp2, Krt6a, Pcdh21, Cfi, Zfpm2, Eya2, Anxa8, Fabp4, Epgn, Myo1a, Gabrp, Nkd1, Clic5, Sprr2f, Sprr2d,  Krt6b, Sprr1b, Keap1, Alcam, Sim2, Spp1, Hbegf, Aldh1a3, Pitx2, F3, Ghrl, Krt17, |

**a) ZD:*Cox-2*^-/-^ vs ZS:*Cox-2*^-/-^** (overrepresented processes among 6007 genes differentially expressed at *P*=0.05 level)

**b) ZD:WT *vs* ZS:WT** (overrepresented processes among 463 genes differentially expressed at *P*=0.05 level)

| **Functional Group 1** | **Median: 3.72E-03** | **p-value** | **Genes** |
| --- | --- | --- | --- |
|  | signal | 1.0E-004 | Ctse, Tff2, Slit3, U46068, Muc1, Gabrp, Ces3, Dmbt1, Defb3, Agr2, Crisp1, Pigr, Penk1, Spp1, Lpl, Nrp1, Cfi, Ear5 |
|  | Secreted | 3.7E-003 | Pigr, Tff2, Penk1, Spp1, U46068, Slit3, Cfi, Defb3, Dmbt1, Agr2 |
|  | disulfide bond | 8.4E-003 | Ctse, Tff2, Slit3, Gabrp, Ces3, Defb3, S100a9, Pigr, Penk1, Lpl, Nrp1, Cfi, Ear5 |
|  | glycoprotein | 1.6E-002 | Abca9, Ctse, Slit3, U46068, Muc1, Gabrp, Ces3, Dmbt1, Crisp1, Pigr, Spp1, Lpl, Nrp1, Cfi, Ear5 |
| **Functional Group 2** | **Median: 1.65E-02** | **p-value** | **Genes** |
|  | keratinization | 0.002 | Sprr2h, Krt6a, Sprr2f |
|  | structural molecule activity | 0.003 | Sprr2h, Ctse, Krt19, F5, Krt6a, Sprr2f, Krt8 |
|  | anatomical structure morphogenesis | 0.030 | Sprr2h, Slit3, Krt6a, Nrp1, Sprr2f, Aldh1a2, Dmbt1, Krt8 |
|  | tissue development | 0.045 | Sprr2h, Spp1, Krt6a, Sprr2f |
| **Functional Group 3** | **Median: 1.81E-03** | **p-value** | **Genes** |
|  | structural molecule activity | 0.003 | Sprr2h, Ctse, Krt19, F5, Krt6a, Sprr2f, Krt8 |
|  | cytoskeleton | 0.004 | Sprr2h, Clasp1, Krt19, Krt6a, Muc1, Sprr2f, Myh1, Cys1, Krt8 |
|  | cytoskeleton organization and biogenesis | 0.009 | Clasp1, Krt19, Krt6a, Myh1, S100a9, Krt8 |
|  | Cell Communication | 0.010 | Krt19, Spp1, Krt6a, Krt8 |

**c) ZD:*Cox-2^-/-^* *vs* ZD:WT** (overrepresented processes among 1860 genes differentially expressed at *P*=0.05 level)

| **Functional Group 1** | **Median: 1.27E-04** | **p-value** | **Genes** |
| --- | --- | --- | --- |
|  | region of interest:Coil 2 | 5.73E-008 | Krt16, Krt18, Krt19, Krt6a, Krt8, Krt17 |
|  | cytoskeleton | 3.12E-005 | Krt16, Mlph, Krt19, Krt20, Vil1, Muc1, Ank2, Ldb3, Krt8, Avil, Krt18, Lrrc26, Krt6a, Krt17 |
|  | structural molecule activity | 2.23E-004 | Krt16, Cldn2, Krt18, Krt19, Krt20, Krt6a, Cldn7, Krt8, Cldn18, Krt17 |
|  | cytoskeleton organization and biogenesis | 3.28E-004 | Krt16, Avil, Krt18, Krt19, Krt20, Vil1, Krt6a, Krt8, Krt17 |
|  | cell differentiation | 0.01 | Mlph, Krt19, Krt20, Nkd1, Dmbt1, Ank2, Krt8, Krt18, Spp1, Npnt, Krt6a, Aldh1a3, Zfpm2, Krt17 |
| **Functional Group 2** | **Median: 0.01** | **p-value** | **Genes** |
|  | Z disc / contractile fiber part | 0.01 | Krt19, Ldb3, Krt8 |
| **Functional Group 3** | **Median: 0.01** | **p-value** | **Genes** |
|  | extracellular region | 0.01 | Epgn, Mcpt2, Muc1, Dmbt1, Cldn7, Tgm2, Fmod, Cldn18, Sftpd, Cldn2, Aqp5, Hgfac, Areg, Spp1, Npnt, Igh-6, Mcpt1, Cfi, Colec11 |
| **Functional Group 4** | **Median: 0.04** | **p-value** | **Genes** |
|  | chemotaxis | 0.04 | Sftpd, Spp1, S100a8, Cxcl17 |

**d) ZS:*Cox-2 ^-/-^* *vs* ZS:WT** (overrepresented processes among 479 genes differentially expressed at *P*=0.05 level)

| **Functional Group 1** | **Median: 0.005** | **p-value** | **Genes** |
| --- | --- | --- | --- |
|  | mitosis | 0.00 | Nusap1, Bub1b, Ccna2, Cenpf, Cdca3, Ccnb1-rs1, Nuf2, Birc5, Plk1, Ube2c, Ccnb2, Cdc25c, Aspm, Cdc20 |
|  | cell cycle | 0.00 | Nusap1, Bub1b, Ccna2, Cdca3, Cdc73, Txnip, Suv39h2, Nuf2, Birc5, Appl1, Hrasls3, Ube2c, Ccnb2, Cdc25c, S100a6, Aspm, Cdc20 |
|  | cell division | 0.00 | Nusap1, Bub1b, Ccna2, Cdca3, Ccnb1-rs1, Nuf2, Birc5, Ube2c, Ccnb2, Cdc25c, Aspm, Kif20a, Cdc20 |
|  | cell cycle process | 0.01 | Nusap1, Ccnjl, Bub1b, Ccna2, Cenpf, Cdca3, Cdc73, Ccnb1-rs1, Suv39h2, Nuf2, Birc5, Plk1, Hrasls3, Igf1r, Ube2c, Etv6, Ccnb2, Aspm, Cdc25c, S100a6, Cdc20 |
|  | Cell cycle | 0.03 | Plk1, Bub1b, Ccna2, Smad4, Ccnb2, Cdc25c, Cdc20 |
|  | regulation of progression through cell cycle | 0.04 | Nusap1, Ccnjl, Bub1b, Ccna2, Cenpf, Cdc73, Birc5, Plk1, Hrasls3, Igf1r, Ube2c, Etv6, Ccnb2, S100a6 |
| **Functional Group 2** | **Median: 0.01** | **p-value** | **Genes** |
|  | microtubule cytoskeleton | 0.00 | Nusap1, Kif2c, Kif22, Polb, Tpx2, Ckap2, Cenpf, Kif23, Birc5, Kif16b, Plk1, Tuba8, Cenpe, Pea15a, Tnrc6a, Ccnb2, Aspm, Kif20a, Kif1b |
|  | Kinesin, motor region | 0.00 | Kif23, Kif22, Kif2c, Kif16b, Cenpe, Kif1b, Kif20a |
|  | microtubule-based process | 0.00 | Nusap1, Kif2c, Kif22, Tpx2, Nuf2, Kif23, Birc5, Tuba8, Cenpe, Kif16b, Tnrc6a, Kif20a, Kif1b |
|  | cytoskeletal part | 0.00 | Nusap1, Kif2c, Kif22, Polb, Tpx2, Ckap2, Cenpf, Kif23, Birc5, Dlg4, Kif16b, Cenpe, Krt76, Plk1, Tuba8, Pea15a, Krt23, Tnrc6a, Aspm, Krt84, Kif20a, Kif1b |
|  | cytoskeleton | 0.01 | Kif2c, Tpx2, Kif23, Birc5, Dlg4, Sprr2k, Tuba8, Krt76, Cenpe, Kif16b, Krt23, Kif1b, Kif20a, Frmd4b, Nusap1, Kif22, Polb, Ptpn14, Muc1, Ckap2, Clic5, Cenpf, Plk1, Pea15a, Tnrc6a, Tgm3, Ccnb2, Aspm, Krt84 |
